# Global, regional, and national time trends in incidence for migraine, from 1990 to 2019: an age-period-cohort analysis for the GBD 2019

**DOI:** 10.1186/s10194-023-01619-9

**Published:** 2023-07-01

**Authors:** Luying Fan, Yuhang Wu, Jiehua Wei, Fan Xia, Yufeng Cai, Senmao Zhang, Junxiang Miao, Yunzhe Zhou, Chu Liu, Wei Yan, Dan Liu, Lizhang Chen, Tingting Wang

**Affiliations:** 1 Department of Epidemiology and Health Statistics, Xiangya School of Public Health, Central South University, 110 Xiangya Road, Changsha, 410078 Hunan China; 2 Department of Biomedical Informatics, School of Life Sciences, Central South University, Changsha, China; 3 Jiangxi Center for Disease Control and Prevention Institute of Chronic Non-Communicable Diseases, Nanchang, China; 4 Prehospital Emergency Department of Xiangtan Central Hospital, Xiangtan, China; 5 Hunan Provincial Key Laboratory of Clinical Epidemiology, Changsha, Hunan 410078 China; 6 NHC Key Laboratory of Birth Defect for Research and Prevention, Hunan Provincial Maternal and Child Health Care Hospital, Changsha, China

## Abstract

**Background:**

The majority of epidemiological studies on migraine have been conducted in a specific country or region, and there is a lack of globally comparable data. We aim to report the latest information on global migraine incidence overview trends from 1990 to 2019.

**Methods:**

In this study, the available data were obtained from the Global Burden of Disease 2019. We present temporal trends in migraine for the world and its 204 countries and territories over the past 30 years. Meanwhile, an age-period-cohort model be used to estimate net drifts (overall annual percentage change), local drifts (annual percentage change in each age group), longitudinal age curves (expected longitudinal age-specific rate), and period (cohort) relative risks.

**Results:**

In 2019, the global incidence of migraine increased to 87.6 million (95% UI: 76.6, 98.7), with an increase of 40.1% compared to 1990. India, China, United States of America, and Indonesia had the highest number of incidences, accounting for 43.6% of incidences globally. Females experienced a higher incidence than males, the highest incidence rate was observed in the 10–14 age group. However, there was a gradual transition in the age distribution of incidence from teenagers to middle-aged populations. The net drift of incidence rate ranged from 3.45% (95% CI: 2.38, 4.54) in high-middle Socio-demographic Index (SDI) regions to -4.02% (95% CI: -4.79, -3.18) in low SDI regions, 9 of 204 countries showed increasing trends (net drifts and its 95% CI were > 0) in incidence rate. The age-period-cohort analysis results showed that the relative risk of incidence rate generally showed unfavorable trends over time and in successively birth cohorts among high-, high-middle-, and middle SDI regions, but low-middle- and low-SDI regions keep stable.

**Conclusions:**

Migraine is still an important contributor to the global burden of neurological disorders worldwide. Temporal trends in migraine incidence are not commensurate with socioeconomic development and vary widely across countries. Both sexes and all age groups should get healthcare to address the growing migraine population, especially adolescents and females.

**Supplementary Information:**

The online version contains supplementary material available at 10.1186/s10194-023-01619-9.

## Introduction

Migraine is a disabling neurological disorder, with recurrent and often debilitating headaches accompanied by neurological symptoms (including nausea, vomiting, and sensory hypersensitivity), affecting an estimated 12% of the population [1, 2]. Experiencing a migraine attack is classified within the highest WHO disability class [3], it results in 45.1 million years lived with disability (YLDs), accounting for 5.6% of the global disease burden and more than all other neurological disorders combined [4, 5]. What’s worse, the disease affects individuals during the most formative and productive periods of their lives (e.g., education completed, families formed, children raised, careers built). In addition, migraine imposes an additional economic burden on patients and their families. The individual direct and indirect costs in the United States alone are estimated at nearly $9000 annually for those with migraine, which is higher than demographically similar people without migraine [6].

Despite these facts, migraine remains one of the most underfunded and under-recognized medical conditions worldwide [7]. There were large differences among inter-country in the migraine burden, and the burden increased significantly from 1990 to 2019. To date, the vast majority of previous studies have only conducted country- or region-specific analyses. Moreover, the results of some studies using descriptive analysis may also be subject to some limitations because traditional methods cannot eliminate the confounding effects of age, period, and cohort. Therefore, an in-depth analysis of the effects of age, period, and birth cohort is needed, and the Global Burden of Disease Study (GBD) database provides the possibility to do so.

This study visualized global migraine incidence data by age, sex, year, and region for the period 1990–2019 from the GBD 2019 to describe the latest epidemiology of migraine globally, updating the results of the previous global burden of migraine studies. Age-period-cohort (APC) model was then used to further examine the effects of age, period, and birth cohort effects on changes in migraine incidence over the previous three decades. Therefore, the results of this study on the spatial–temporal trends in migraine incidence can be helpful in identifying the underlying factors contributing to these variations and can illustrate the shifting disease patterns, providing guidance for the development of prevention strategies and management measures to reduce the burden of the migraine.

## Method

### Data source

Data used in this study were obtained from the Global Health Data Exchange GBD Results Tool (http://ghdx.healthdata.org/gbd-results-tool). In GBD 2019, migraine was defined as a disabling primary headache disorder, typically characterized by recurrent moderate or severe unilateral pulsatile headaches. In the International Classification of Diseases versions 9 and 10, it is denoted by the codes 346 through 346.93 and G43-G43.919, respectively [8]. We obtained the incidence number, all-age incidence rate and age-standardized incidence rate of migraine by sex (female, male and both), location (204 countries and territories), age (5–84 years old), year (from 1990 to 2019), and Social-demographic index (SDI) from GBD 2019. The definition of incidence was the number of new cases of a given cause during a given period in a specified population. In the GBD tool, it was expressed as the number of new cases in a year divided by the mid-year population size. The SDI was calculated from lag distributed income per capita, total fertility rate under the age of 25, and mean education for those aged 15 and older to show the location's development status. The 204 countries and territories were categorized into five quintiles: high SDI (> 0.81), high-middle SDI (0.70–0.81), middle SDI (0.61–0.69), low-middle SDI (0.46–0.60), and low SDI (< 0.46). All estimates were reported in 95% uncertainty intervals (UIs), which were obtained by repeatedly sampling the sample 1000 times, whose upper and lower bounds were derived based on the 2.5th and 97.5th percentiles of the uncertainty distribution [9]. Details of the methodological information and modelling strategies in GBD 2019 have been published elsewhere [9, 10]. The relevant data were anonymous and publicly available, and a waiver of informed consent was reviewed and approved by the University of Washington Institutional Review Board.

### Statistics analysis

#### Analysis of overall temporal trends in migraine incidence

This study reported global migraine incidence and its spatial and temporal trends from 1990 to 2019. Temporal trends in incidence over the study period were assessed by all-age incidence rate, age-standardized incidence rate, and the relative change of incidence in percentage between 1990 and 2019. The age distribution of the global population from the GBD 2019 study was utilized to standardize incidence rates per 100,000 person years [11]. Besides, we examined the age distribution of incidences by arranging a number of incidences into eight age strata (5–9, 10–14, 15–19, 20–24, 25–29, 30–39, 40–49, 50–84 years) and calculating the proportions of incidences from each age stratum.

#### Age-period-cohort modelling analysis of incidence data

The APC model is a statistical method used to extract and reveal possible information about illness patterns as well as to assess the contributions of age, period, and cohort effects on the outcomes. In this study, we mainly focused on the following estimable functions. Net drift reflects the overall annual percentage change. Local drifts reflect annual percentage changes for each age group. longitudinal age curve indicates the fitted longitudinal age-specific rates in the reference cohort adjusted for period deviations. Period relative risk (RR) indicates the period relative risk adjusted for age and nonlinear cohort effects in each period relative to the reference one; cohort RR indicates the cohort relative risk adjusted for age and nonlinear period effects in each cohort relative to the reference one. When the RR value is more than 1, it suggests that the factor increases the risk of migraine incidence. When the RR value is less than 1, it suggests that the factor decreases the risk of migraine incidence. To address the identification problem caused by linear relationships between age, period, and cohort, the intrinsic estimator (IE) method associated with the APC model was used in our study, thus overcoming the drawback of model parameters being unpredictable. More methodological information is described in previous literature [12].

GBD 2019 incidence estimates for migraine and population data of each country/region were used as data inputs for the APC model with IE method. In this model, it is required that the age and period intervals must all be equal, so we divided the population aged 5–84 years into 16 age groups (5–9, 10–14, …, 80–84) with a group distance of 5 years. The groups under 5 years old and over 85 years old were excluded from this study due to the absence or rarity of migraine events. We arranged GBD data into a single unit framework by selecting the incidence and population counts from the mid-year of six time point values (1992, 1997, …, 2017) instead of the average of the 5-year periods to represent the specific period. The input data included 16 age groups and 21 partially overlapping ten-year birth cohorts, as referenced by the mid-year of birth, from 1906 to 1914 (the 1910 cohort) to 2006 to 2014 (the 2010 cohort). The lexis diagram of GBD data for the APC model was shown in Additional Table 1. We used the Wald chi-squared test to test the significance of the estimated parameters and functions. All statistical tests were two-tailed. The APC analysis for this study utilized the APC Web Tool (http://analysistools.nci.nih.gov/apc/) from the National Cancer Institute. All the graphics were produced with the R statistical program (version 4.0.3).

## Results

### Trends of incidence rate of migraine, 1990–2019

**Fig. 1:**
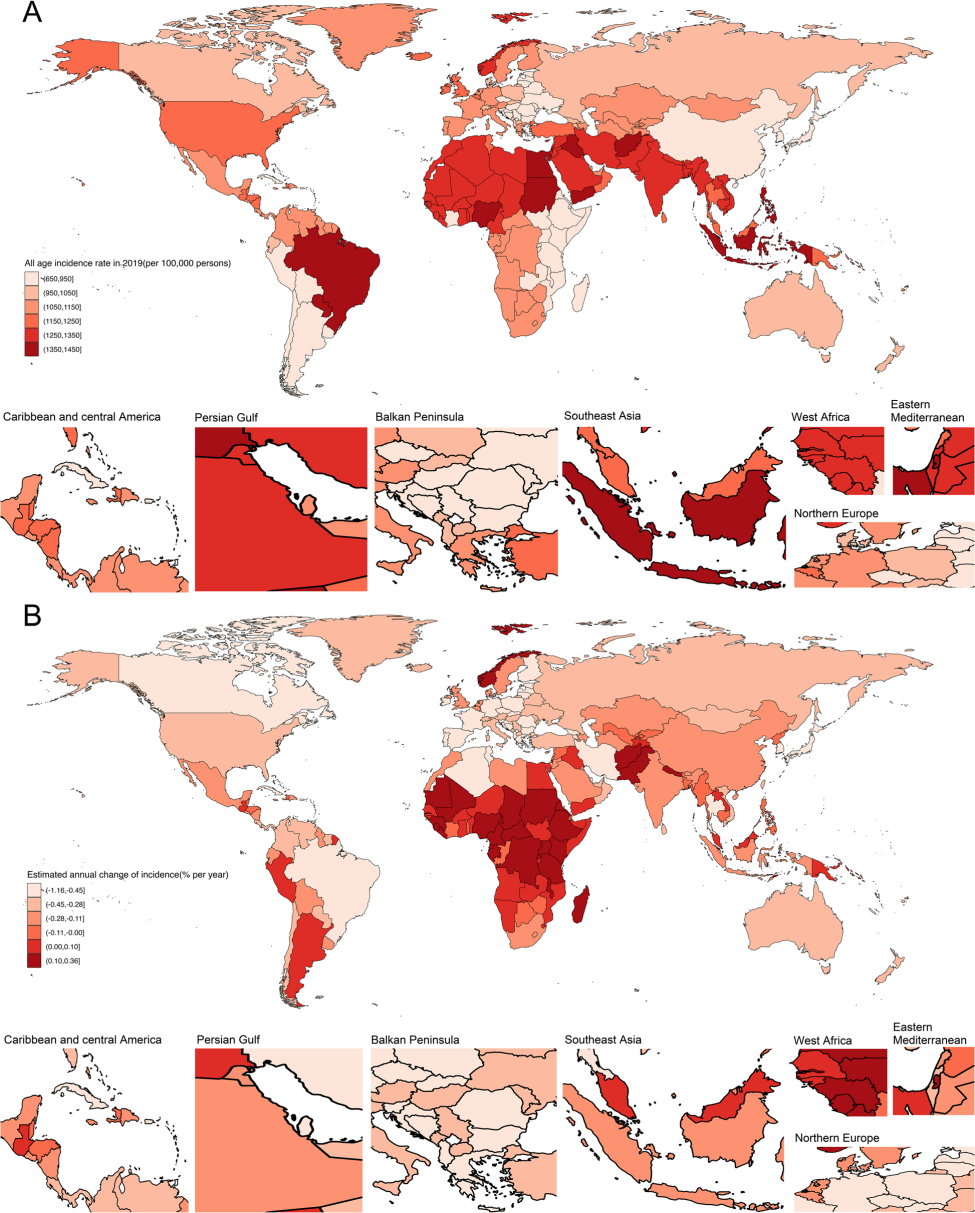
The all-age incidence rate and net drift in 204 countries and territories. **A** World map of all-age incidence rate for migraine in 2019. The global all-age incidence rate was 1132.79 (95% UI: 990.45, 1275.02) per 100,000 population. **B** World map of net drifts for migraine incidence rate. Net drift captures components of the trends attributable to calendar time and successive birth cohorts. The global net drift of migraine incidence rate was 0.089% (95 CI: -0.005, 0.183)

Table 1, Fig. 1, Additional Fig. 1 and Additional Table 2 show the population, total number of incidences, all-age incidence rate, age-standardized incidence rate, and net drift of incidence rate. In the past three decades, the number of migraine incidence increased from 62.6 million (95% UI: 54.5, 71.0) in 1990 to 87.6 million (95% UI: 76.6, 98.7) in 2019, with the age-standardized incidence rate was 1142.54 (95% UI: 995.9, 1289.44) per 100,000 population. Globally, the APC model estimated a net drift of migraine incidence rate was 0.089% (95% CI: -0.005, 0.183) from 1990 to 2019 (Table 1).

**Table 1:** Trends in migraine incidence rate across Socio-demographic Index quintiles, 1990âˆ’2019

	Global (*N *= 204)		High SDI (*N* = 41)		High-middle SDI (*N *= 41)		Middle SDI (*N* = 40)		Low-middle SDI (*N* = 41)		Low SDI (N = 41)	
	1990	2019	1990	2019	1990	2019	1990	2019	1990	2019	1990	2019
Population												
Number, n	5,349,847,687 (5,238,894,009, 5,459,638,741)	7,737,464,623 (7,482,639,908, 7,992,501,511)	8.22E + 08	1.01E + 09	1.15E + 09	1.43E + 09	1.72E + 09	2.4E + 09	1.13E + 09	1.76E + 09	5.28E + 08	1.13E + 09
Percentage of global, %	100	100	15.4	13.1	21.5	18.5	32.1	39.6	21.1	22.8	9.9	14.6
Incidences												
Number, n	62,585,283 (54,459,799, 70,979,260)	87,648,969 (76,635,688, 98,654,602)	9,488,136(8,323,560, 10,641,927)	10,451,773 (9,214,844, 11,732,312)	12,690,478 (11,074,182, 14,289,470)	14,746,658 (13,014,486, 16,553,543)	20,428,973 (17,767,242, 23,110,638)	27,654,960 (24,344,576, 31,015,012)	14,137,339 (12,145,938, 16,167,573)	21,915,270 (19,025,662, 24,837,027)	5,805,439 (4,904,231, 6,717,440)	12,829,042 (10,860,141, 14,818,974)
Percentage of global, %	100	100	15.2	11.9	20.3	16.8	32.6	31.6	22.6	25	9.3	14.6
Percent change of incidences 1990–2019, %	40.05 (36.84, 43.46)		10.16 (7.99, 12.7)		15.7 (11.78, 20.1)		32.74 (28.03, 37.89)		53.3 (47.9, 58.14)		112.51 (109.2, 115.85)	
All-age incidence rate												
Rate per 100,000	1169.85 (1017.97, 1326.75)	1132.79 (990.45, 1275.02)	1154.26 (1012.58, 1294.62)	1031.37 (909.31, 1157.74)	1103.11 (962.61, 1242.1)	1030.94 (909.85, 1157.26)	1189.98 (1034.93, 1346.18)	1153.94 (1015.81, 1294.14)	1251.48 (1075.2, 1431.2)	1242.37 (1078.56, 1408.01)	1099.22 (928.58, 1271.9)	1136.64 (962.2, 1312.95)
Percent change of rate 1990–2019, %	-3.17 (-5.38, -0.81)		-10.65 (-12.4, -8.59)		-6.95 (-10.1, -3.41)		-4.91 (-8.28, -1.23)		-1.83 (-5.29, 1.27)		-0.56 (-2.11, 1.00)	
Age-standardized incidence rate												
Rate per 100,000	1119.53 (977.26, 1262.34)	1142.54 (995.9, 1289.44)	1199.73 (1042.34, 1357.11)	1219.59 (1059.44, 1376.83)	1078.79 (942.74, 1215.21)	1121.24 (979.29, 1266.04)	1106.91 (969.11, 1242.76)	1162.4 (1018.18, 1305.51)	1172.49 (1024.75, 1322.31)	1177.03 (1023.69, 1328.1)	1051.73 (910.42, 1193.79)	1048.94 (909.02, 1191.58)
Percent change of rate 1990–2019, %	2.06 (1.13, 2.84)		1.66 (0.41, 2.72)		3.45 (2.38, 4.54)		3.04 (2.08, 3.99)		-0.71 (-2.04, 0.51)		-4.02 (-4.79, -3.18)	
APC model estimates												
Net drift of incidence rate, % per year	0.089 (-0.005, 0.183)		0.053 (-0.047, 0.154)		0.103 (0.01, 0.196)		0.15 (0.059, 0.242)		-0.003 (-0.095, 0.088)		-0.008 (-0.103, 0.087ï¼‰	

Notes: All-age incidence rate: crude incidence rate

Age-standardized incidence rate is computed by direct standardization with global standard population in GBD 2019

Net drifts are estimates derived from the age-period-cohort model and denotes overall annual percentage change in incidence rate, which captures the contribution of the effects from calendar time and successive birth cohorts

Parentheses for all GBD health estimate indicate 95% uncertainty intervals; parentheses for net drift indicate 95% confidence intervals

*SDI* Socio-demographic Index, *APC* Age-period-cohort

Regionally, the all-age incidence rate for migraine increased from 1030.94 (95% UI: 909.85, 1157.26) per 100,000 population in high-middle SDI regions to 1242.37 (95% UI: 1078.56, 1408.01) per 100,000 population in low-middle SDI regions, whereas the age-standardized incidence rate was highest in the high SDI regions (1219.59, 95 UI: 1059.44, 1376.83 per 100,000 population) and lowest in the low SDI regions (1048.94, 95% UI: 909.02, 1191.58 per 100,000 population). Similar patterns can be found in the net drift results estimated by the APC model (Table 1).

At the national level, India (17.9 million, 95% UI: 15.8, 20.1), China (12.9 million, 95% UI: 11.5, 14.5), United States of America (3.8 million 95% UI: 3.4, 4.2), Indonesia (3.5 million, 95% UI: 3.1, 4.0) were the top four in the number of migraine incidents, accounting for 43.6% of migraine incidents globally. In 2019, the countries with the highest and lowest all-age incidence rates were Paraguay (1435.68, 95% UI: 1211.11, 1694.58 per 100,000 people) and Japan (651.03, 95% UI: 575.26, 727.36 per 100,000 people). Around one-third of the nations had an increase in the incidence rate for all ages, with Equatorial Guinea seeing the biggest percentage increase. The highest age-standardized incidence rate was observed in Italy (1528.3, 95% UI: 1345.37, 1709.32 per 100,000 population) and the lowest age-standardized rates were identified in Ethiopia (692.59, 95% UI: 605.2, 776.68 per 100,000 population), with net drifts changed from 0.329% (95% CI: 0.217, 0.442) in Peru to -0.161% (95% CI: -0.256, -0.066) in Qatar (Fig. 1, Additional Fig. 1, Additional Table 2).

### Time trends in migraine incidence rate across different age groups

**Fig. 2:**
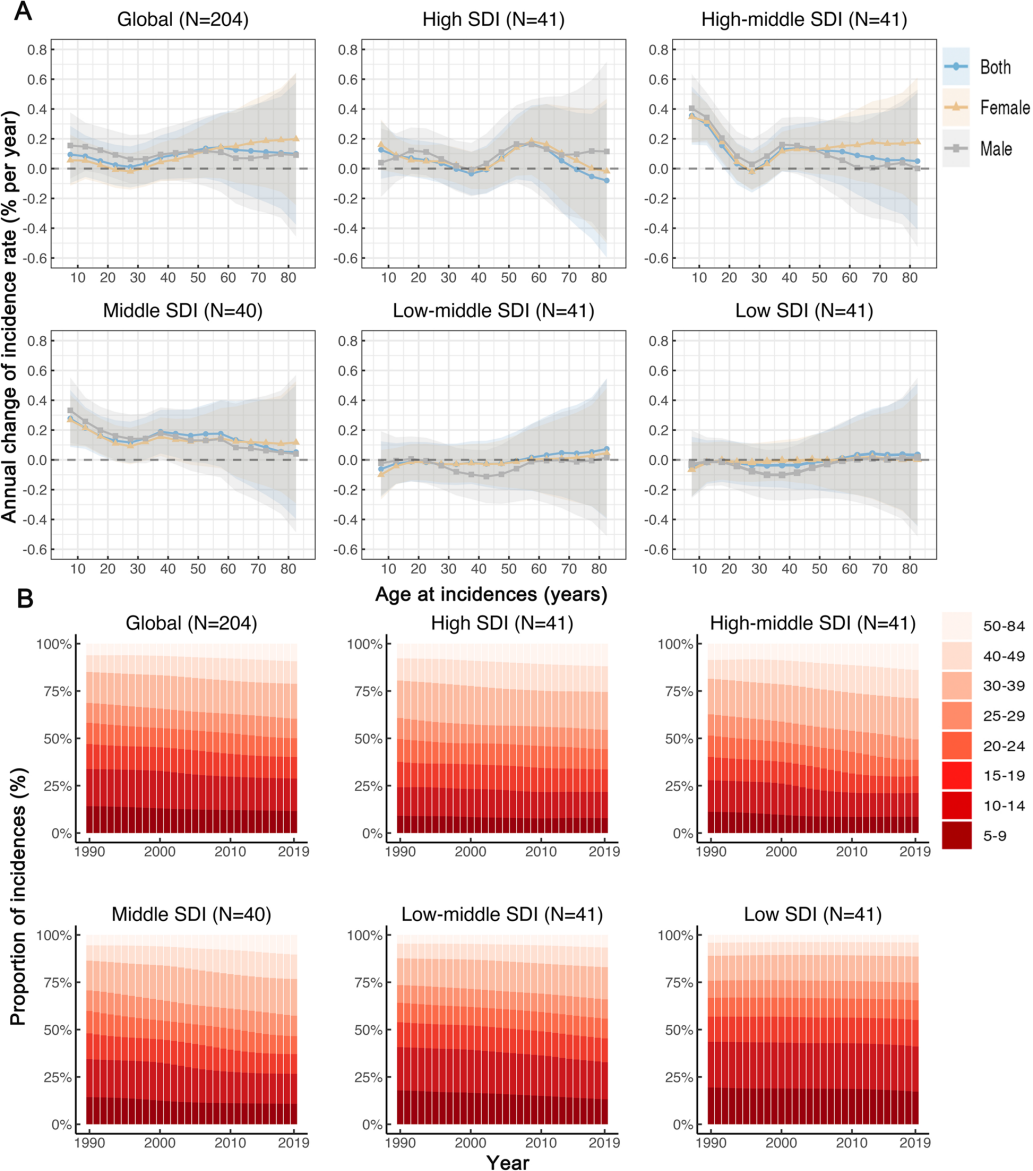
Local drifts of incidence rate and age distribution of incidences by SDI quintiles, 1990–2019. **A** Local drifts of migraine incidence rate (estimates from age-period-cohort models) for 16 age groups (5–9 to 80–84 years), 1990–2019. The dots and shaded areas indicate the annual percentage change of incidence rate (% per year) and the corresponding 95% CIs. **B** Temporal change in the relative proportion of migraine incidences across age groups (5–9, 10–14, 15–19, 20–24, 25–29, 30–39, 40–49, 50–84 years), 1990–2019. SDI: Socio-demographic Index

Figure 2A shows the annual percentage change in migraine incidence rate for each age group. Globally, values of local drift were mainly above 0 for most age groups, indicating that most age groups experienced an increase in the incidence rate of migraine. Similar patterns were observed in high, high-middle, and middle SDI regions, whereas the incidence rate of migraine remained nearly constant in low and low-middle SDI regions from 1990 to 2019. The local drift of incidence rate for each country is shown in Additional Fig. 2–6.

Figure 2B presents temporal changes in the age distribution of incidences. Globally, more than 40% of incidents occurred among teenagers under the age of 20 in 1990. From 1990 to 2019, there was a gradual increase in the number of migraine incidences in middle-age people. The same patterns were observed in SDI regions except for low SDI regions. The age distribution of incidences for each country is shown in Additional Fig. 7–11.

### Age, period and cohort effects on migraine incidence rate

**Fig. 3:**
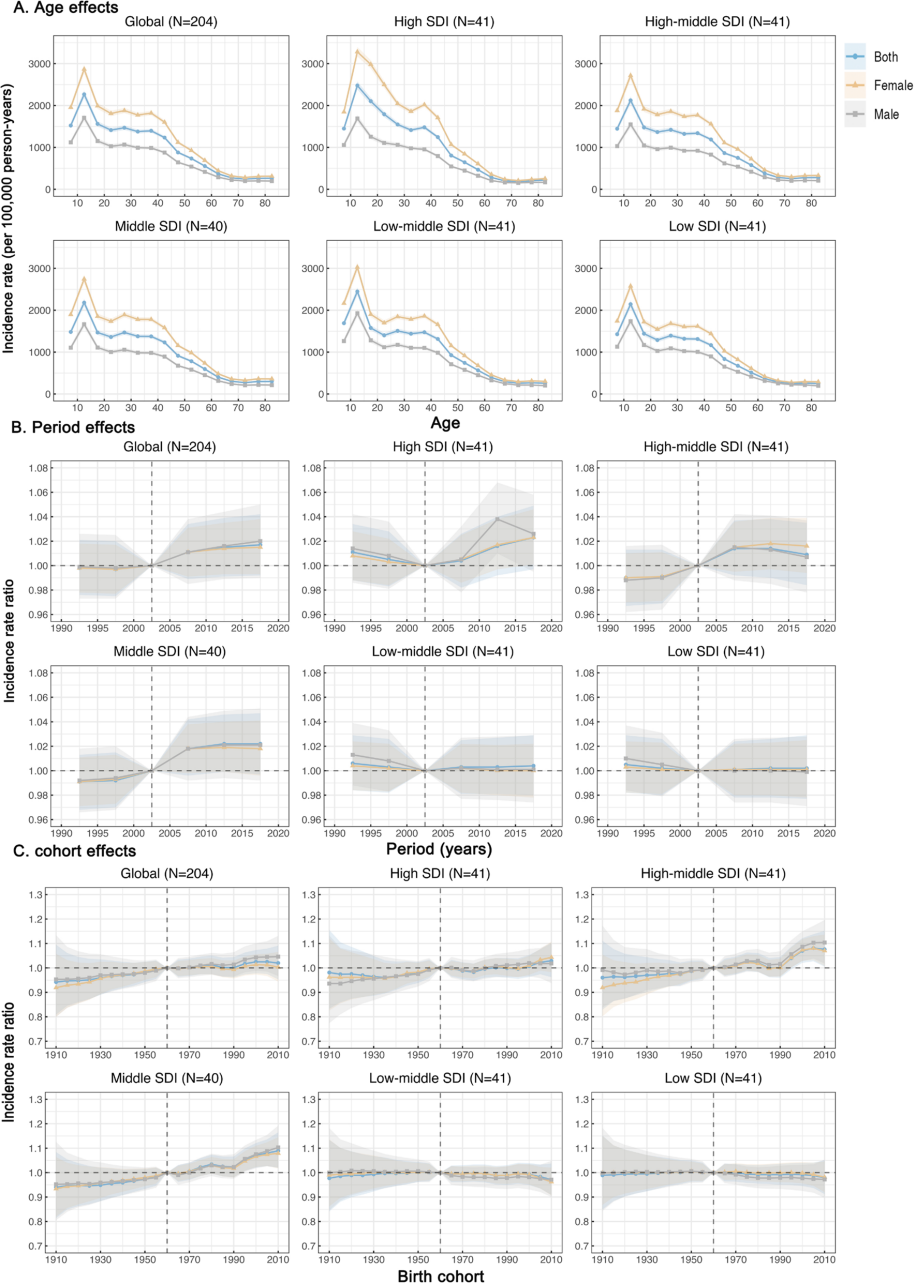
Age, period and cohort effects on migraine incidence rate by SDI quintiles. **A** Age effects are shown by the fitted longitudinal age curves of incidence rate (per 100,000 person-years) adjusted for period deviations. **B** Period effects are shown by the relative risk of incidence rate (incidence rate ratio) and computed as the ratio of age-specific rates from 1990–1994 to 2015–2019, with the referent cohort set at 2000–2004. **C** Cohort effects are shown by the relative risk of incidence rate and computed as the ratio of age-specific rates from the 1910 cohort to the 2010 cohort, with the referent cohort set at 1960. The dots and shaded areas denote incidence rates or rate ratios and their corresponding 95% CIs. *SDI* Socio-demographic Index

Figure 3 presents the estimates for age, period, and cohort effects by SDI quintile. Generally, similar patterns of age effects were observed across all SDI quintiles. The highest risk was observed in the 10–14 age group and the risk decreased with age, suggesting that worse survival occurred in adolescents (Fig. 3A). High-SDI regions displayed an overall higher incidence rate across all age groups as compared to other SDI regions. Sex difference in age effects was found globally.

Globally, period effects revealed an increased risk of incidence rate after 2005 (Fig. 3B). In middle, high-middle, and high SDI regions period effects had been escalating over time. Middle SDI regions showed a considerable increase, indicating that the occurrence of migraine had not been effectively controlled over time. However, period effects in low-middle and low SDI regions have remained essentially consistent over the previous three decades.

Globally, cohort effects showed a slight increase in the successive birth cohort (Fig. 3C). Similar patterns were observed in the high SDI and middle SDI regions. The risk of the cohort effect increased with the birth cohort in high-middle SDI regions, especially after 1990, while it remained constant in low-middle and low SDI regions. The age, period, and cohort effects on migraine incidence rate in each country are shown in Additional Fig. 12–26.

### Age-period-cohort effects in exemplary countries

To better characterize the significant changes in migraine incidence rate by age-period-cohort effects globally, we offered several representative countries across SDI quintiles. Additional Fig. 27A shows countries with favorable age-period-cohort effects, indicating a decreased risk for migraine incidence. Germany showed an overall decreasing trend of incidence rate with age, particularly at 65–84 years, more favorable period effects were observed since 2012. Republic of Korea stood out for its notable net drift and demonstrated an emerging transition in the age distribution of incidences. For the birth cohort effect, the risk of migraine incidence dropped dramatically. Turkey showed relatively modest local drifts in incidence rate across age groups, with a gradual reduction in the relative risk of incidence rate across periods and birth cohorts. Italy was a high-middle SDI country with the highest age-standardized rate, showing a decreasing trend of incidence rate in adults aged over 20, with significantly decreased risk over the periods. The incidence rate of migraine in Brazil showed a steep drop from 10–14 to 25–29 years, with the risk of period effects decreasing over time. Tuvalu, a low-middle SDI country, exhibited a decrease in the incidence rate of migraine in most age groups, with period effects dropping constantly.

Additional Fig. 27B presents countries with relatively unfavorable age-period-cohort effects on incidence rate, indicating an increased risk for migraine incidence. In Japan, the incidence rate increased considerably with age, and notable increases in risks were found in the period after 2005 and across all birth cohorts. Among high SDI regions, Singapore exhibits the worst trends in migraine incidence rates, and local drifts greater than 0% per year are observed in all age groups, despite their attenuation with age. Additionally, period and cohort risks deteriorated throughout the entire study. Similar to other middle SDI countries, China and Peru showed a transition in the age distribution of incidences, with significantly increased risks of period effects and cohort effects across the entire study. India is the country with the highest number of incidences, which has an increased incidence rate among people aged 55–84 years. The United Republic of Tanzania was the only low SDI country with a significant incidence rate increase for all age groups, with markedly increased risks in those born after 2000.

## Discussion

Controlling the incidence rate of migraine is the key way to make progress in reducing the burden of neurological diseases. Migraine is a chronic and often lifelong disease, and the high incidence of migraine contributes significantly to the burden of disability worldwide. The age-standardized incidence rates of migraine have largely remained unchanged, despite considerable advancements in many aspects over the past three decades. Socioeconomic development does not determine the extent of incidence from migraine, while the APC model reflects the substantial health disparities and potential priority setting of migraine incidence in the three dimensions of age, period and birth cohort in countries around the world. Females and young and middle-aged people, in particular, are the main affected groups for migraine attacks, highlighting the focus on achieving UN Sustainable Development Goal targets.

Here, for the first time, we use the APC framework to explore the secular trends in migraine incidence on a global scale from 1990 to 2019, and our findings support horizontal comparisons between different regions and countries. Before this study, some traditional epidemiological analyses provided systematic estimates of migraine burden at the global, regional, and national levels [5, 13-16]. The unique contributions of this study to this field include: i) we have updated and analyzed the disease incidence trends in detail, highlighting success points and potential key areas in a timely manner; ii) we have delved into the use of data to provide more accurate information and background in response to primary disease prevention strategies. Specifically, we can accurately distinguish and capture the independent effects of age, period, and cohort effects on disease incidence trends at the global, regional, and national levels. Another important piece of information is that the APC model, which examines both the overall incidence rate and age standardized incidence rate, has a significant advantage in quantifying the burden of disease.

Between 1990 and 2019, the total number of migraine incidences worldwide increased by 40.05%, with the increase being relatively greater in low-middle SDI and low SDI regions. The rapid increase in population is the main driving force, and the number of migraine attacks may continue to increase. Particularly, after eliminating the inconsistency in age composition, some information changed differently, and the age-standardized incidence rate of migraine is basically unchanged globally during these 30 years (just increased by 2.06%). The complete clinical experience accumulation of neurological healthcare workers and mature diagnosis and treatment technical system has achieved encouraging progress in diagnosis, intervention treatment and prognosis over the past three decades. However, the accompanying social factors, such as stress from various aspects (employment, education, and marriage), lifestyle (smoking, physical activity, obesity), environmental air pollution, etc., increased the risk of migraine [17-21] . These factors are closely associated with societal patterns, but it is independent of the level of social development, because the difference in age standardized incidence rate of migraine among SDI regions has not been observed in the current study, which indicates that the burden of migraine does not change significantly with socioeconomic development (Table 1). The potential association of the migraine burden with socioeconomic background remains unclear, and the results of available studies are inconsistent [13, 22-24]. One piece of information that cannot be ignored is the differences in the diagnosis and treatment of migraine in the SDI region, which may confound this association. In low-income countries, relative poverty is associated with poor access to health-care and rural housing [25]. Interestingly, our results further reveal that the relative risk of incidence rate generally showed unfavorable trends over time and in successively birth cohorts among high-, high-middle-, and middle SDI regions (Fig. 3). A plausible hypothesis is that some behaviors influenced by the local cultural background and the potential stigmatization of headache disorders in society indirectly weaken the ability to control the disease burden with continuous economic growth and social development [22].

Significant variations in migraine incidence rates exist between countries, thus highlighting the need for tailored prevention and therapeutic strategies based on the individual burden of the disease. A multitude of factors, including biological, psychological, and social, have been linked to the development of migraine [26]. However, recent findings have raised questions about the causality of these associations, with age, period, and cohort effects warranting further investigation. In light of these considerations, we have focused on the analysis of migraine incidence patterns in selected countries utilizing the APC framework. The incidence of migraine across all age groups in the Republic of Korea has seen a nearly 30% reduction, with successive birth cohorts showing even more significant improvements over the study period. These positive trends are likely the result of healthcare initiatives aimed at reducing migraine incidence rates. The Korean Headache Society (KHS), established in 1999, has launched several media campaigns aimed at raising awareness of the impact of migraine on patients' quality of life and advocating for effective treatment strategies [27]. The Brazilian Health System ensures that every citizen has access to medical care without incurring any costs. With over 250,000 community health agents and more than 35,000 organized groups of healthcare professionals, the system is well-equipped to provide education and effective treatment across all regions of Brazil [28]. Although notable progress has been made, there is still scope for further improvement as the country's age-standardized incidence rate of migraine remains higher than the global average. In Brazil, there is no nationwide systematic program specifically aimed at reducing the burden of migraine [29]. Similarly, while Government-funded Medicaid provides free health insurance to all in Turkey, it is under-resourced [29]. Italy has a national healthcare system that is regulated at its 21 regional levels, with over 80 specialized headache and migraine centers available to serve its population of 60 million [29].

China, with a population of 1.42 billion, is the most populous country in the world. Although the all-age incidence rate for migraine decreased by 4.79% during 1990 − 2019, it still accounted for the second largest absolute number of migraine attacks globally. In China, all children must receive China's compulsory education programs and take countless exams. Even as adults, individuals face pressure to enter higher education institutions and compete with their peers, exposure to adverse environmental and lifestyle factors during this period has increased the risk of migraine attacks [30]. Additionally, the geographical distribution of neurological facilities is uneven in China, the enormous disparity in medical resources and care among different provinces are likely to be further contributing factors [31]. Due to the widespread use of electronic products, physical inactivity, and aging, migraine attacks will also be more common in middle-aged and elderly populations [32] Singapore is a multi-ethnic, wealthy country in South-East Asia, with the age-standardized incidence rate for migraine increased by 16.04% during 1990 − 2019, which is much higher than other countries in the world. The major problem faced by primary care physicians in Singapore is that the diagnosis of migraine remains difficult and inconsistent, prophylactic treatment has also not been fully utilized [33]. For these countries, there is a pressing need to improve health services that educate primary care physicians and the public about strategies for effective headache diagnosis and treatment. In addition, Southeast Asian transboundary haze, which has affected Singapore over the last few decades, could have a significant negative impact on migraine incidences [34].

Japan, India, Peru and United Republic of Tanzania represent the impact of unfavorable age-period-cohort effects on incidence rate in countries with different socioeconomic development. Japan is a high-SDI country, with a low level of all-age incidence rate in the world but worsening trends over time and cohort. The impact of the stigmatizing attitudes on Japan is substantial. 72% of individuals did not seek medical consultation for migraine despite the fact that 63% of patients indicated that they were unable to cope with their illness, because they were concerned about the damage to their interpersonal relationships and feel guilty about burdening bosses and colleagues with migraine [35]. Despite superior diagnostic and treatment technologies in Japan, appropriate medical services have not yet been adequately utilized to improve the increasingly severe environment for migraine. Public education concerning migraine is one of the most urgent issues in Japan [36] Peru, a middle-SDI country, has been plagued by unfavorable cohort effects and an increase in period risks since the turn of the twenty-first century. Its decentralized health-care system is administered by public sector (Layered, bottom-up) and private sector (unstructured), with a lack of horizontal integration [29]. Furthermore, the management of migraine in Peru has been given low priority and insufficient investment [29]. India is the second most populous country in the world, with the highest number of migraine attacks. Emphasis needs to be placed on the identification and control of triggers for migraine specific to India, including those caused by the country's geographical location (heat and humidity), the fasting habits in different communities, the application of henna, and the stress of travel in crowded conditions [37]. Like elsewhere in the world, there are many additional barriers, such as inadequacies within the health-care system and neglect towards migraine, making migraine management much more difficult in India. This is almost helpless, because as long as other major health problems (such as tuberculosis, malaria, HIV, etc.) are not controlled, we cannot expect focus on an invisible misery like migraine [37]. The same may be true for United Republic of Tanzania.

The prominent position of headache disorders in special populations has received little attention in global health policy debates. Migraine has clinical features in people of different ages, especially in children, and children sometimes cannot describe what they are feeling, which makes migraine diagnosis in children even more challenging than in adults [38]. Furthermore, poor management and treatment during early childhood could potentially lead to an increase in migraine in adults [39]. It is paramount to identify and address migraine attacks in adolescence, in an effort to prevent escalation of symptoms in adulthood. In addition, the disorder tends to remit with older age, an onset of migraine after the age of 50 years should arouse suspicion of a secondary headache disorder [40]. The findings from structural and functional brain MRIs have confirmed sex-related differences in migraine attacks [41]. Several physiological and psychological differences are believed to play a key role in higher migraine incidence in females, such as fluctuations of sex hormones during menstruation, pregnancy, and menopause, genetic factors, exposure to environmental stressors, and response to stress and pain [41-43]. Our findings suggest that males and females in different periods of life may have different triggering factors for migraine, and future guidelines on personalized migraine management and treatment should be fully considered.

The present research findings present a thought-provoking question in this field: what is the optimal course of action? The migraine chronification is considered a threshold issue: certain predisposing factors, combined with frequent headache pain, lower the threshold of migraine attacks, thereby increasing the risk of migraine [44]. Despite noteworthy progress in comprehending migraine pathophysiology, there is still a lot of critical information that needs to be confirmed. Among these is identifying the true triggering factors for migraine attacks, the mechanism by which they precipitate attacks, and how personal they are [45]. We also need to pay attention to some comorbidities, such as epilepsy [46], depression and anxiety [47], stroke [48], myocardial infarction [49], as these comorbidities seem to have a bidirectional relationship with migraine [45]. On the other hand, it is evident that there is a need to further improve migraine management globally, and it is the best practice to shift our focus and resources toward a primary prevention strategy for migraine. Primary care is the first and principal setting in which improvements in migraine epidemic and disease burden should be made, and headache is the most common presenting neurological symptom in primary care [50]. A statistical report highlights the importance of raising awareness and eliminating stigma [51]. General practitioners and community doctors are the main providers of primary care [52], and it is widely recognized that education and training play a pivotal role in implementing and maintaining effective headache services and management [53]. In certain developing countries, poor public health education and inadequate education and training for healthcare professionals may exacerbate the challenges faced [54]. Furthermore, Preventive migraine therapy is used to reduce the frequency, severity, and duration of migraine attacks [55], but the potential adverse effects of overdose must be carefully considered. National governments may develop health policies tailored to their unique national conditions based on their individual requirements and resource availability to support the improvement of migraine burden. In all countries, the multiple causes of suboptimal care include inadequate awareness among health professionals and politicians [54]. It may be possible to improve migraine management by refining health care utilization and management, promoting education and training of health-care professionals, and increasing access to medication and reimbursement, which are critical aspects in the future.

Here, we present an example of in-depth analysis of disease trends using updated GBD data. APC model enables us to observe the shift in incidence risk for each country and captures significant trends in particular populations to provide targeted suggestions through time periods and birth cohorts analyses, which is more effective than conventional epidemiological metrics. Admittedly, several limitations should be acknowledged: I) many of the undeveloped or under developing countries do not have proper data on migraine, and incidence estimates driven by covariates for these countries still have wide uncertainty bounds, although the data gaps are constantly being filled by the GBD collaborators to make model estimates more robust, hence, some results obtained by incidence estimates need to be interpreted with caution; II) this study lacks more granular analysis to capture subnational differences, because there are still differences in health issues and access to health care providers and services at the subnational level, and evidence-based health decision making at the subnational level is crucial for every country; III) the uncertainty of data quality control (collection procedures, handing and coding) remains inevitable, although substantial efforts have been made in data standardization; IV) period and cohort effects are examined in this analysis based on the estimated cross-sectional data of GBD from 1990–2019, and future cohort studies in different countries are needed to determine location- and time‐specific relative risks to evaluate different risks in vulnerable populations.

In conclusion, migraine is an important contributor to the global burden of neurological disorders worldwide. The temporal trends of migraine incidence are not commensurate with socioeconomic development from 1990 to 2019. Unfavorable period and cohort effects reveal that migraine is neglected and that there are currently large gaps in the management of migraine in many countries of the world. Efforts should be made to establish a worldwide observatory of migraine targeting specific regions and countries, rather than being standardized globally. The scope of healthcare to improve the progression of migraine attacks can be extended to both sexes and all age groups, with particular attention to vulnerable populations, including young and middle-aged individuals and females.

## Supplementary Information

**Additional file 1.**

## Data Availability

The dataset supporting the conclusions of this article is available in the Global Burden of Disease Study (http://ghdx.healthdata.org/gbd-results-tool) repository.

## Abbreviations

*WHO*: World health organization
*YLDs*: Years lived with disability
*GBD*: Global Burden of Disease Study
*APC*: Age-period-cohort
*SDI*: Social-demographic index
*RR*: Relative risk
*KHS*: Korean Headache Society
*UN*: United nations
*HIV*: Human immunodeficiency virus
