# Supplementary material for: Global, regional, and national time trends in incidence for migraine, from 1990 to 2019: an age-period-cohort analysis for the GBD 2019
Source: J Headache Pain. 2023 Jul 1;24(1):79. doi: 10.1186/s10194-023-01619-9 (PMC10314517; doi:10.1186/s10194-023-01619-9)
Supplement: Supplementary file 1 — Additional file 1. [file 10194_2023_1619_MOESM1_ESM.pdf]

Additional file 1

Figure 1 The world map of age-standardized incidence rate for migraine, 1990-2019

Figure 2 The local drifts of migraine incidence rate in High SDI countries, 1990-2019

Figure 3 The local drifts of migraine incidence rate in High-middle SDI countries, 1990-2019

Figure 4 The local drifts of migraine incidence rate in Middle SDI countries, 1990-2019

Figure 5 The local drifts of migraine incidence rate in Low-middle SDI countries, 1990-2019

Figure 6 The local drifts of migraine incidence rate in Low SDI countries, 1990-2019

Figure 7 Age distribution of incidences from migraine in High SDI countries, 1990-2019

Figure 8 Age distribution of incidences from migraine in High-middle SDI countries, 1990-2019

Figure 9 Age distribution of incidences from migraine in Middle SDI countries, 1990-2019

Figure 10 Age distribution of incidences from migraine in Low-middle SDI countries, 1990-2019

Figure 11 Age distribution of incidences from migraine in Low SDI countries, 1990-2019

Figure 12 Age effects on migraine incidence rate in High SDI countries

Figure 13 Age effects on migraine incidence rate in High-middle SDI countries

Figure 14 Age effects on migraine incidence rate in Middle SDI countries

Figure 15 Age effects on migraine incidence rate in Low-middle SDI countries

Figure 16 Age effects on migraine incidence rate in Low SDI countries

Figure 17 Period effects on migraine incidence rate in High SDI countries

Figure 18 Period effects on migraine incidence rate in High-middle SDI countries

Figure 19 Period effects on migraine incidence rate in Middle SDI countries

Figure 20 Period effects on migraine incidence rate in Low-middle SDI countries

Figure 21 Period effects on migraine incidence rate in Low SDI countries

Figure 22 Cohort effects on migraine incidence rate in High SDI countries

Figure 23 Cohort effects on migraine incidence rate in High-middle SDI countries

Figure 24 Cohort effects on migraine incidence rate in Middle SDI countries

Figure 25 Cohort effects on migraine incidence rate in Low-middle SDI countries

Figure 26 Cohort effects on migraine incidence rate in Low SDI countries

Figure 27 Favorable (A) and unfavorable (B) age-period-cohort effects on exemplar countries across SDI quintiles

Table 1 The lexis diagram of GBD data for the APC model

Table 2 Time trends in migraine incidence rate for both sexes in 204 countries, 1990-2019

Figure 1 The world map of age-standardized incidence rate for migraine, 1990-2019

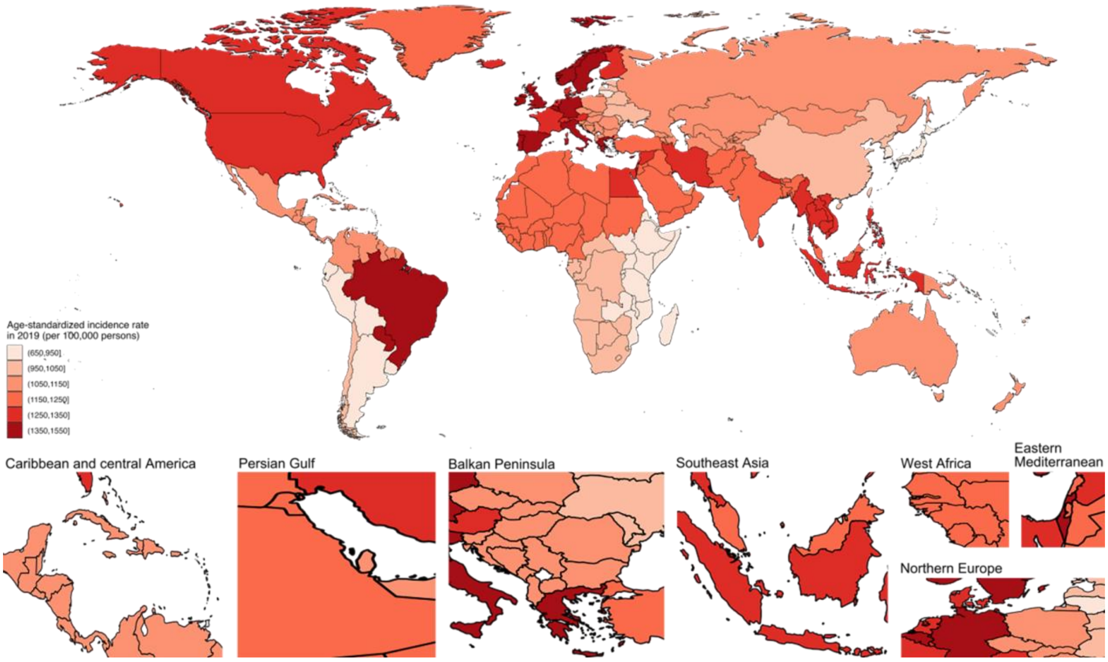

Note: In 2019, global age-standardized incidence rate was 1142.54 (95%UI: 995.9, 1289.44) per 100,000 population.

Figure 2 The local drifts of migraine incidence rate in High SDI countries, 1990-2019

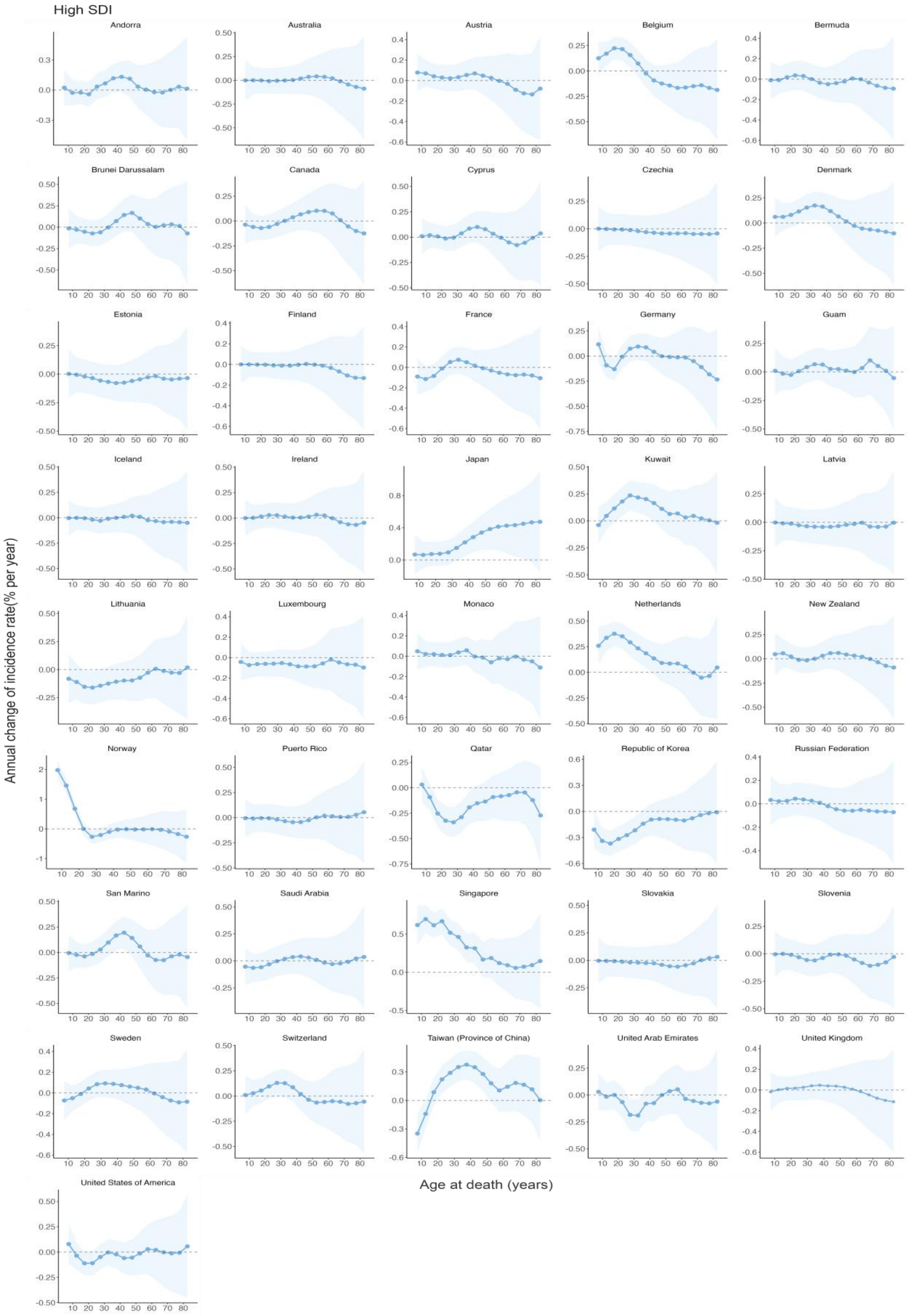

The dots and shaded areas indicate the values of local drift (annual percentage change in incidence rate) and its 95% CIs for migraine in 16 age groups (5-9 to 80-84 years). SDI=Socio-demographic Index.

Figure 3 The local drifts of migraine incidence rate in High-middle SDI countries, 1990-2019

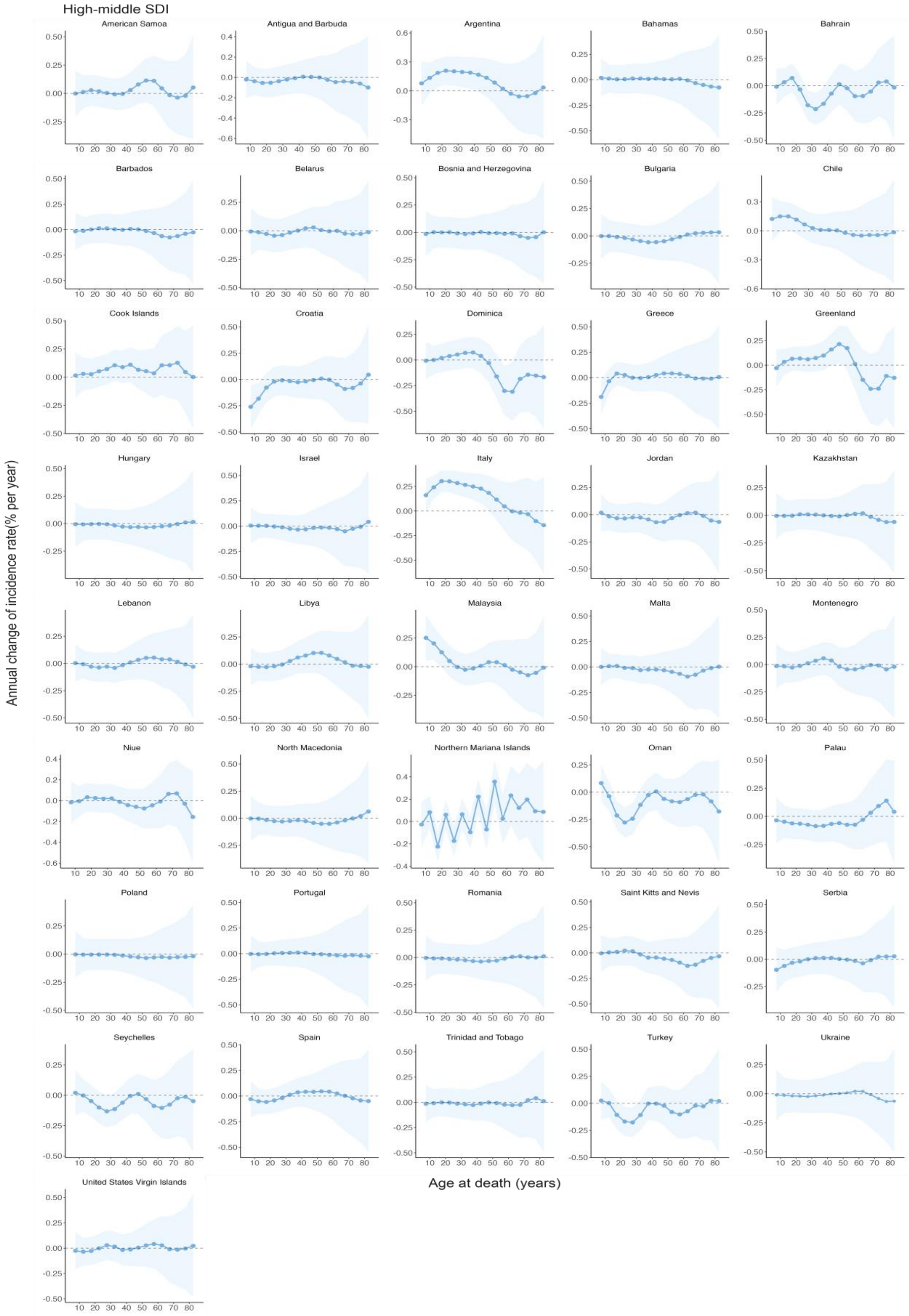

The dots and shaded areas indicate the values of local drift (annual percentage change in incidence rate) and its 95% CIs for migraine in 16 age groups (5-9 to 80-84 years). SDI=Socio-demographic Index.

Figure 4 The local drifts of migraine incidence rate in Middle SDI countries, 1990-2019

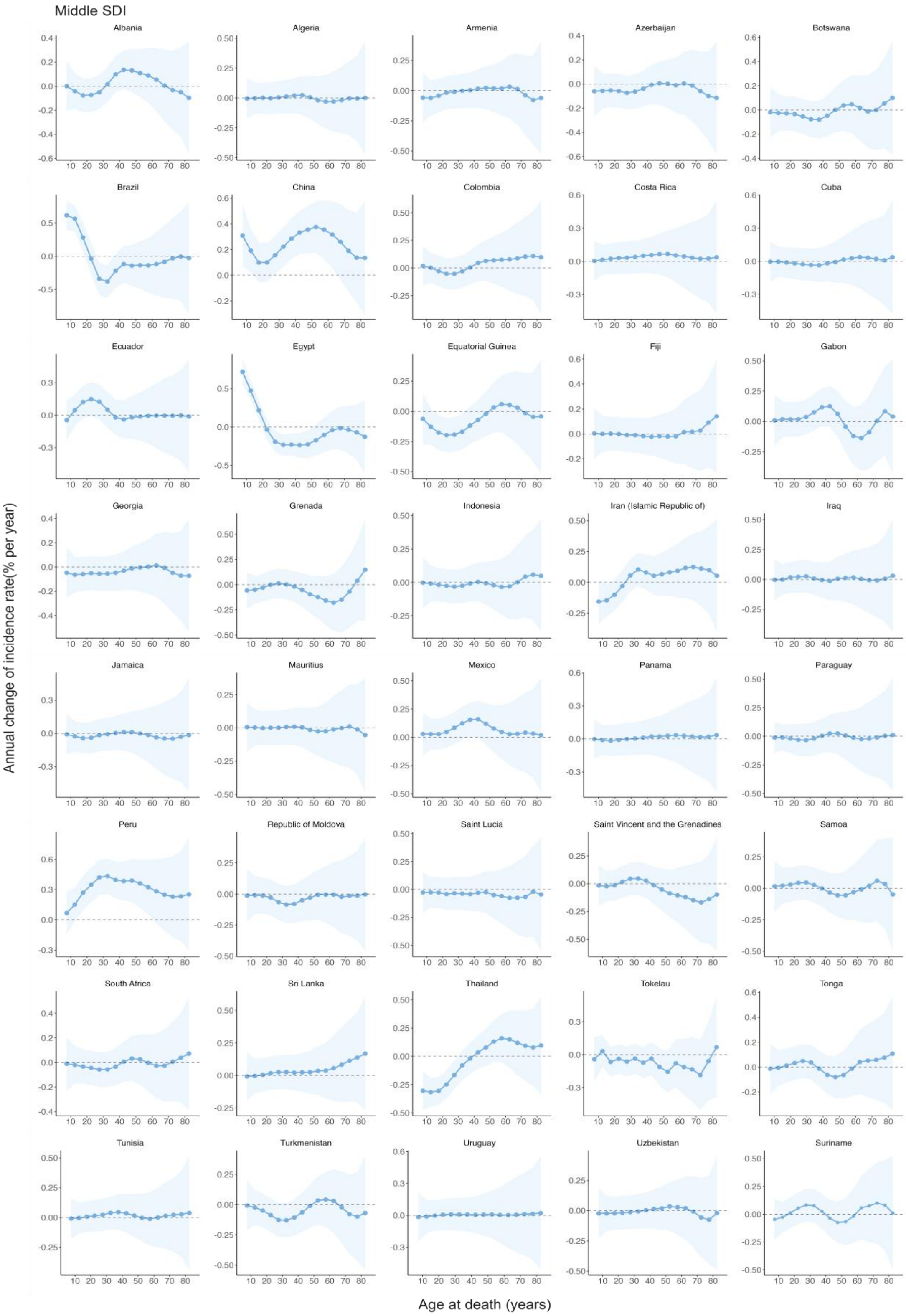

The dots and shaded areas indicate the values of local drift (annual percentage change in incidence rate) and its 95% CIs for migraine in 16 age groups (5-9 to 80-84 years). SDI=Socio-demographic Index.

Figure 5 The local drifts of migraine incidence rate in Low-middle SDI countries, 1990-2019

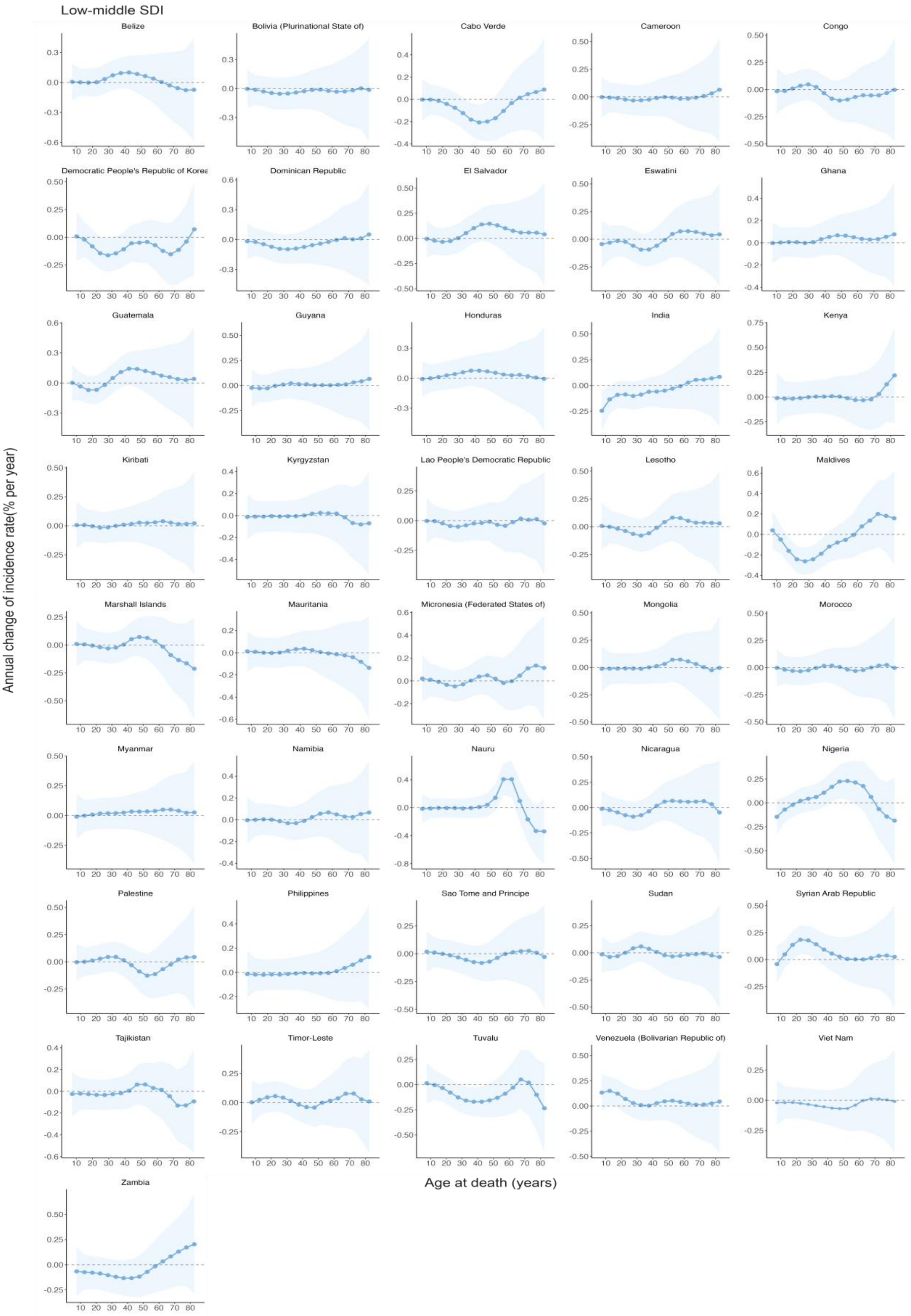

The dots and shaded areas indicate the values of local drift (annual percentage change in incidence rate) and its 95% CIs for migraine in 16 age groups (5-9 to 80-84 years). SDI=Socio-demographic Index.

Figure 6 The local drifts of migraine incidence rate in Low SDI countries, 1990-2019

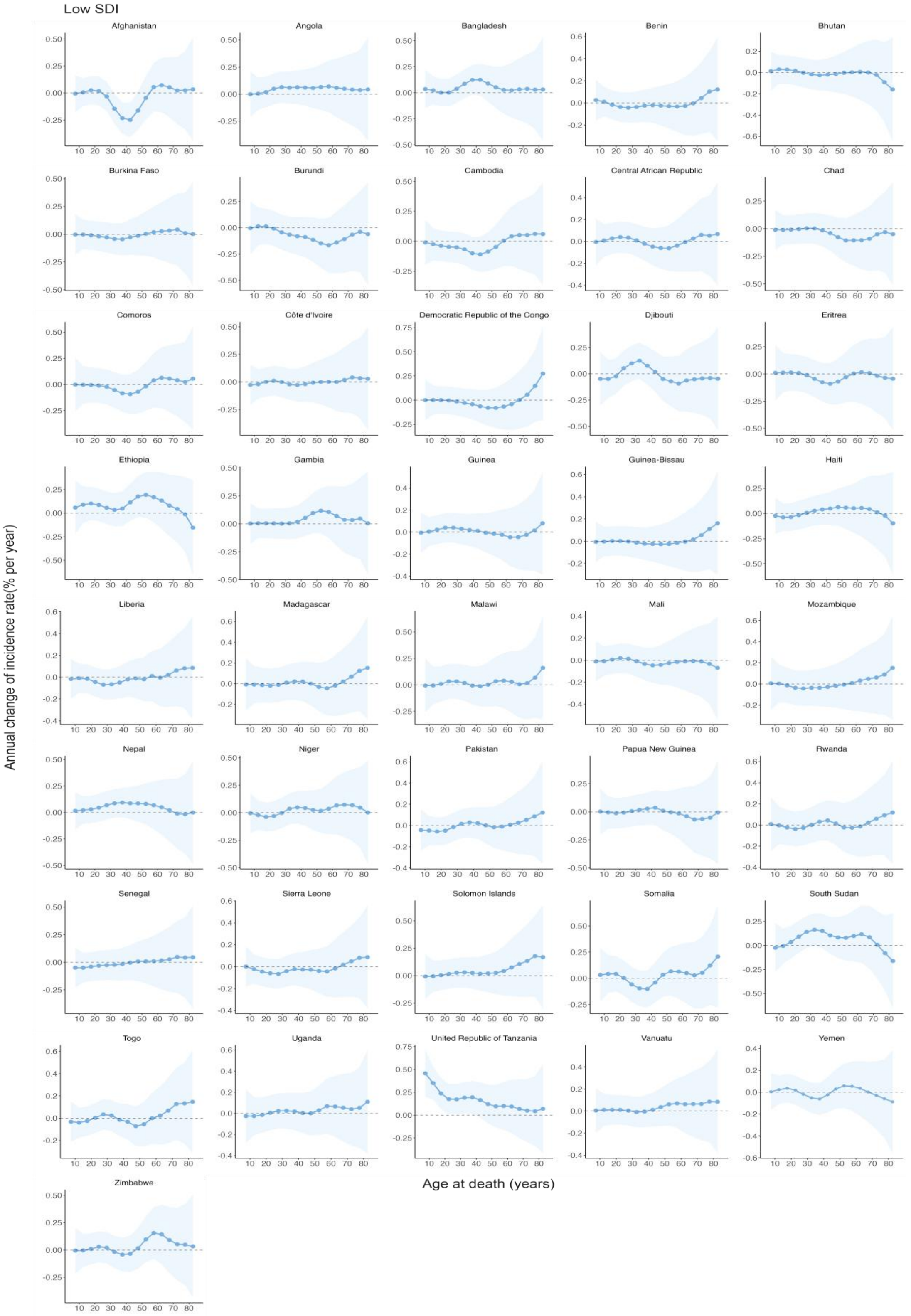

The dots and shaded areas indicate the values of local drift (annual percentage change in incidence rate) and its 95% CIs for migraine in 16 age groups (5-9 to 80-84 years). SDI=Socio-demographic Index.

Figure 7 Age distribution of incidences from migraine in High SDI countries, 1990-2019

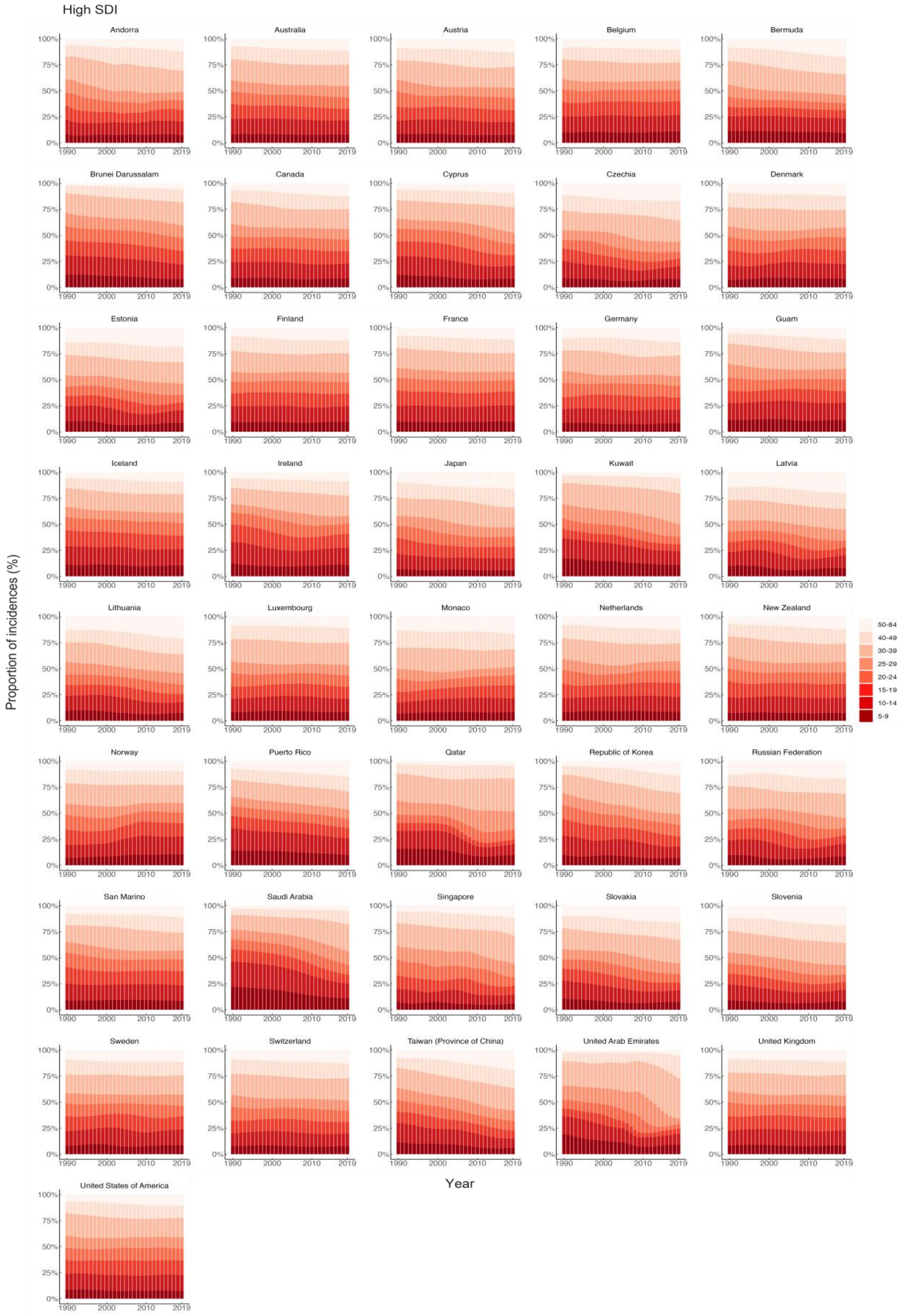

Age distribution of incidences is represented as temporal change in the relative proportion of incidences across age groups (5-9, 10-14, 15-19, 20-24, 25-29, 30-39, 40-49, 50-84 years) during 1990-2019. SDI=Socio-demographic Index.

Figure 8 Age distribution of incidences from migraine in High-middle SDI countries, 1990-2019

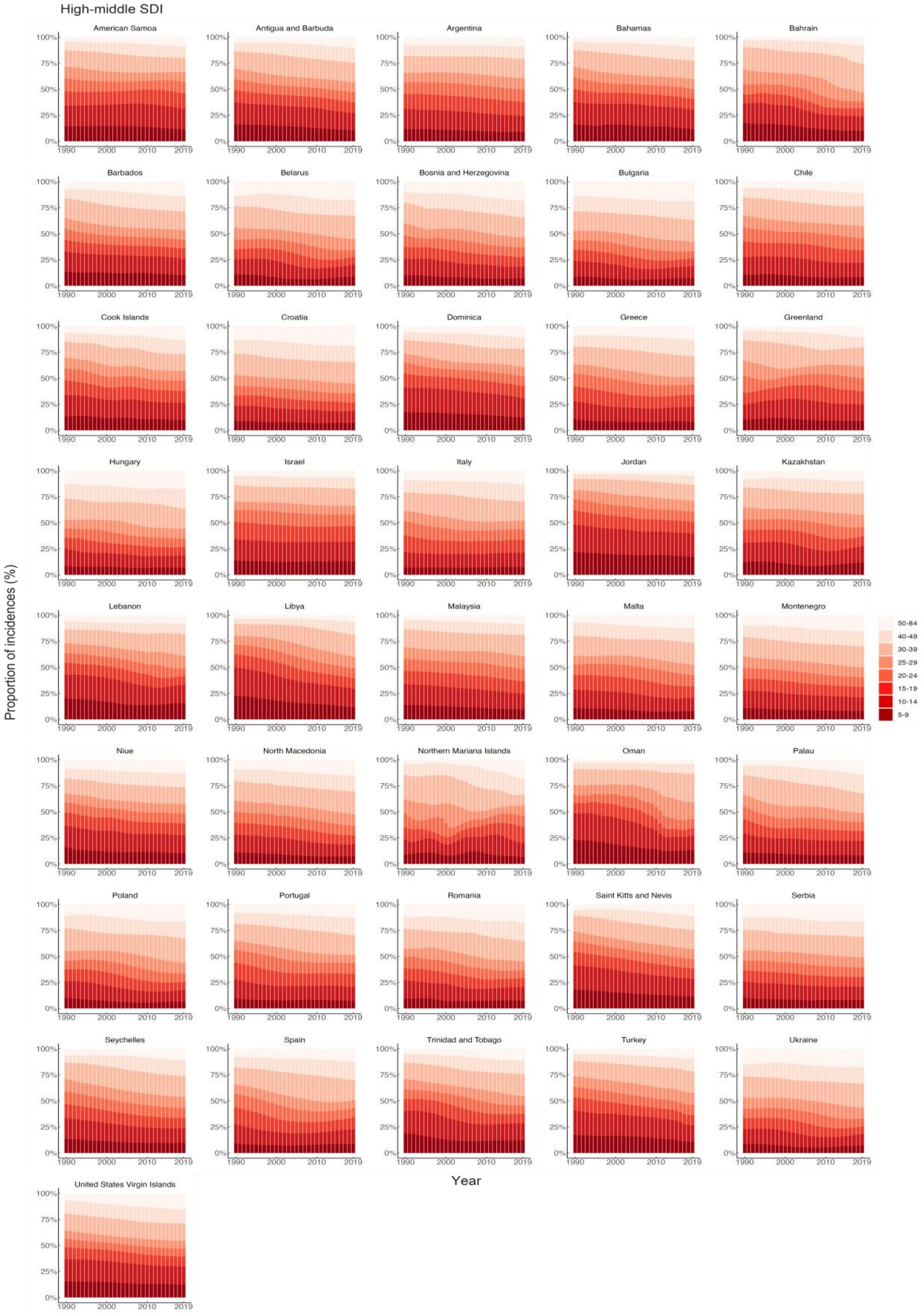

Age distribution of incidences is represented as temporal change in the relative proportion of incidences across age groups (5-9, 10-14, 15-19, 20-24, 25-29, 30-39, 40-49, 50-84 years) during 1990-2019. SDI=Socio-demographic Index.

Figure 9 Age distribution of incidences from migraine in Middle SDI countries, 1990-2019

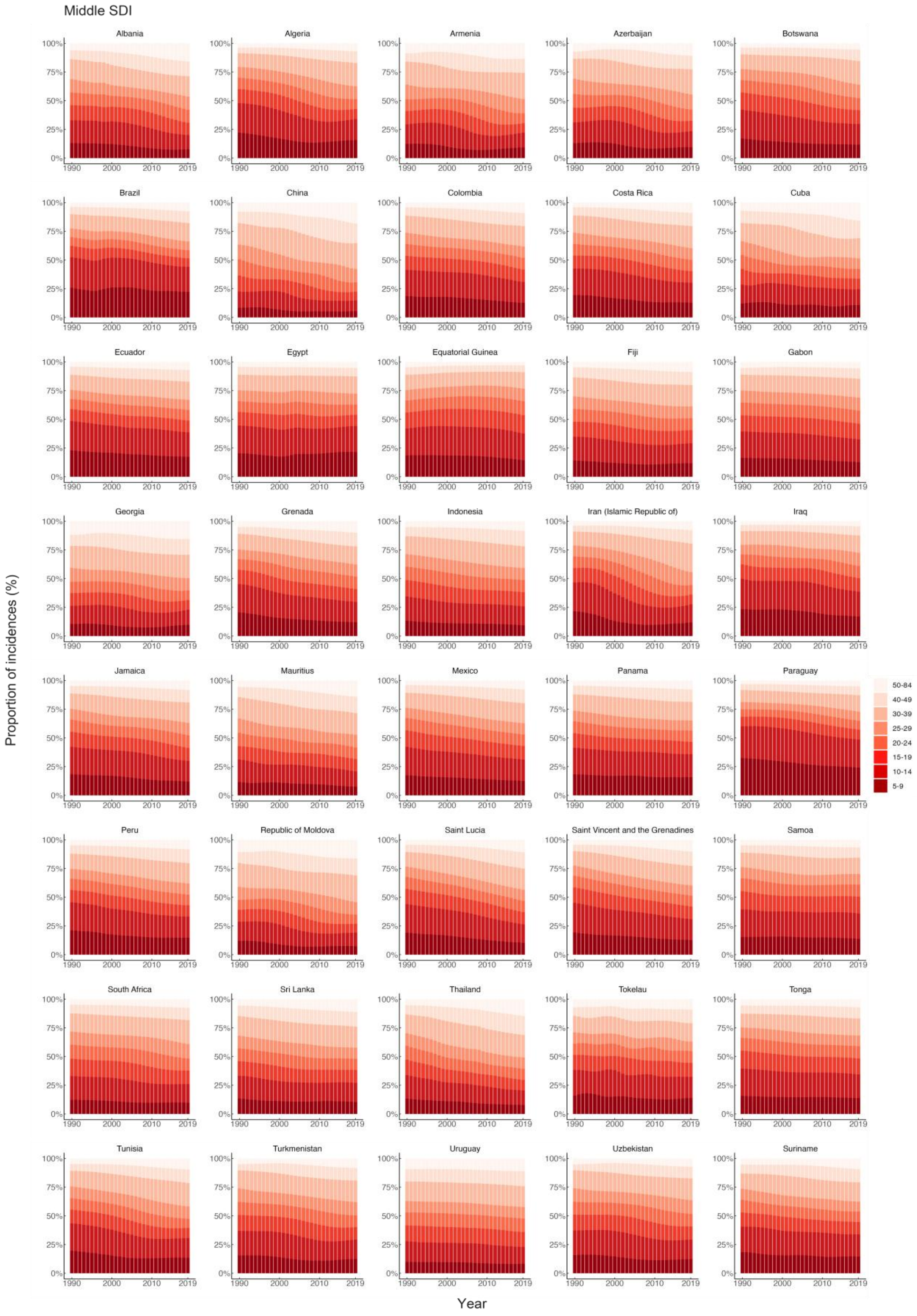

Age distribution of incidences is represented as temporal change in the relative proportion of incidences across age groups (5-9, 10-14, 15-19, 20-24, 25-29, 30-39, 40-49, 50-84 years) during 1990-2019. SDI=Socio-demographic Index.

Figure 10 Age distribution of incidences from migraine in Low-middle SDI countries, 1990-2019

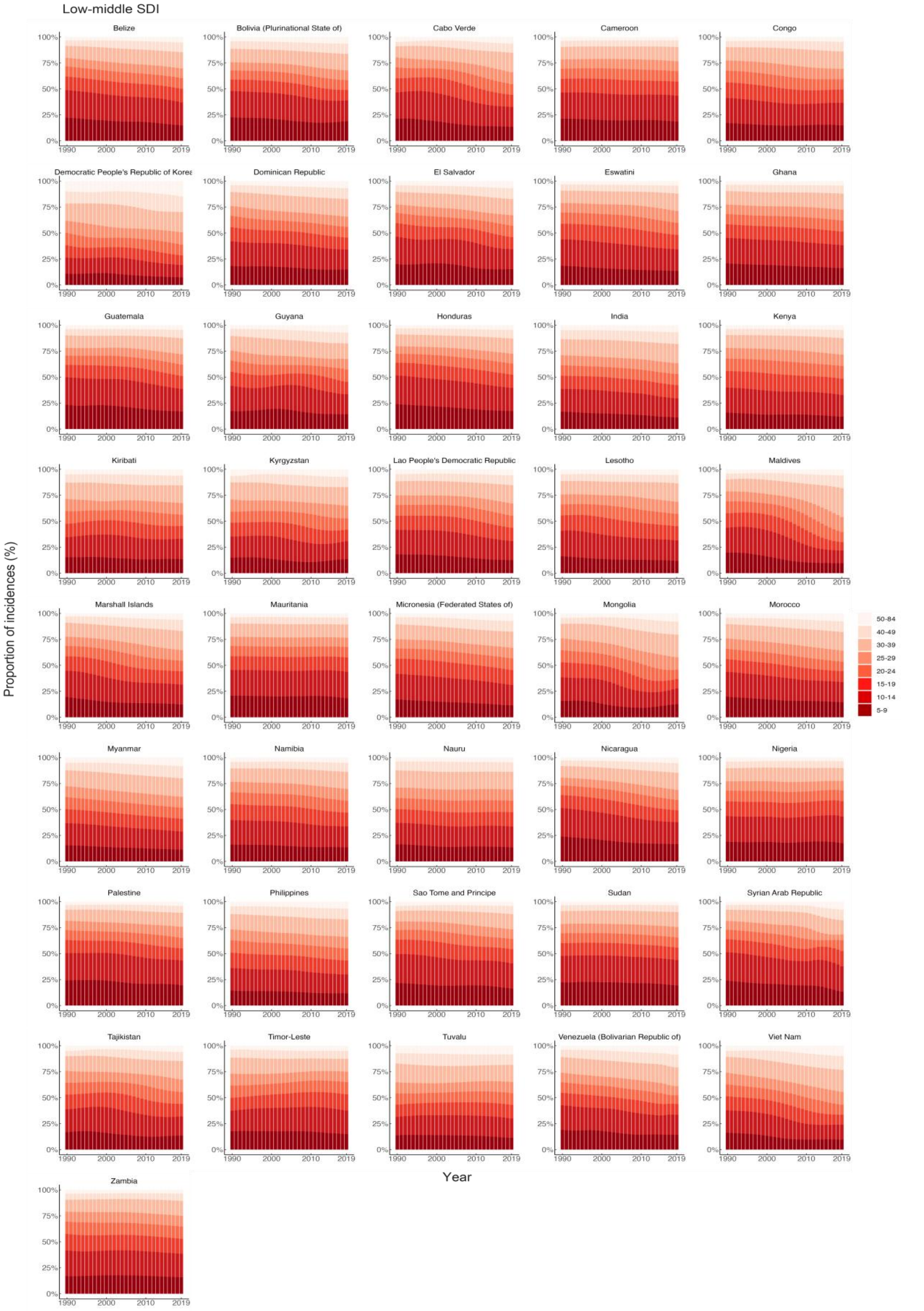

Age distribution of incidences is represented as temporal change in the relative proportion of incidences across age groups (5-9, 10-14, 15-19, 20-24, 25-29, 30-39, 40-49, 50-84 years) during 1990-2019. SDI=Socio-demographic Index.

Figure 11 Age distribution of incidences from migraine in Low SDI countries, 1990-2019

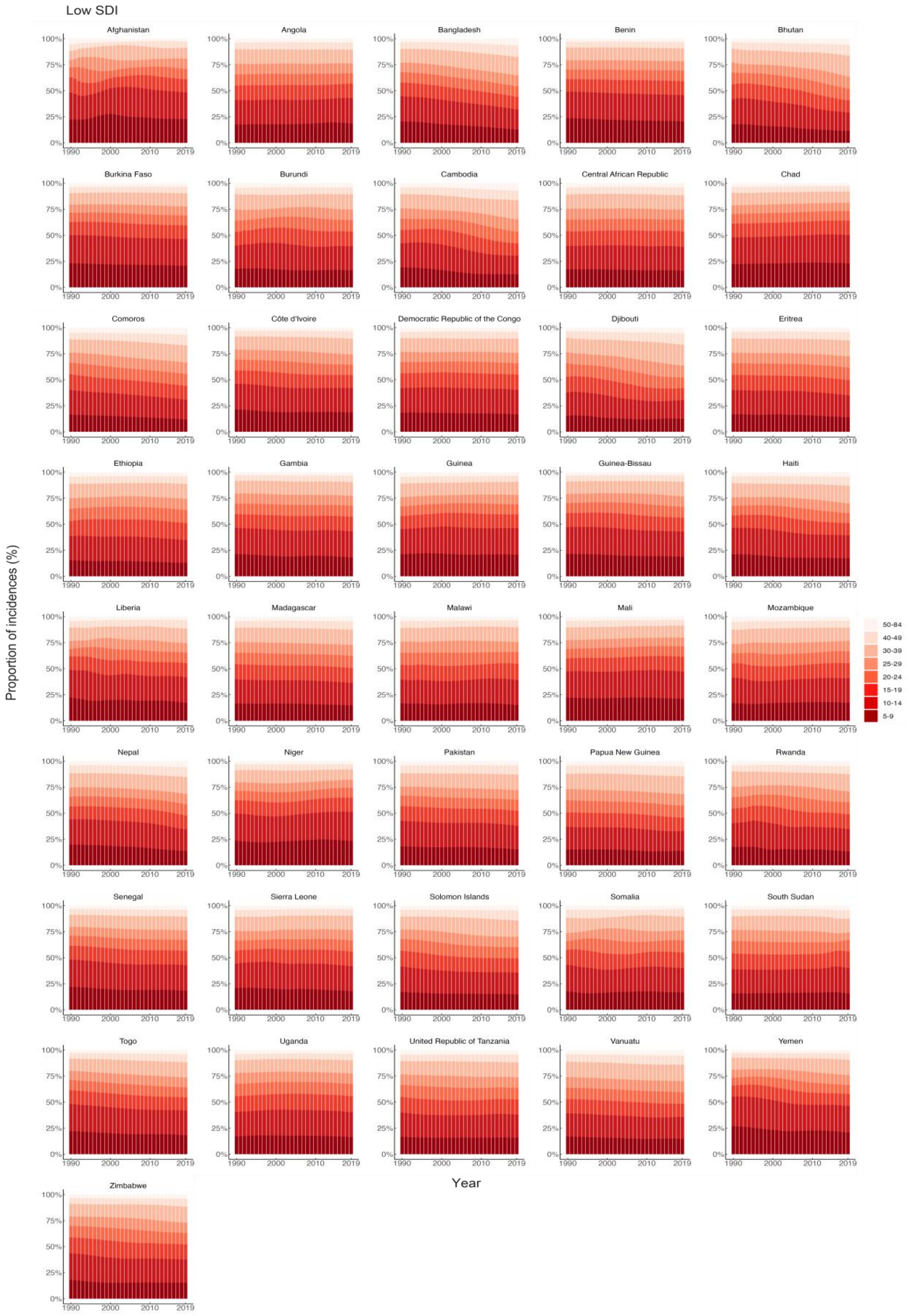

Age distribution of incidences is represented as temporal change in the relative proportion of incidences across age groups (5-9, 10-14, 15-19, 20-24, 25-29, 30-39, 40-49, 50-84 years) during 1990-2019. SDI=Socio-demographic Index.

Figure 12 Age effects on migraine incidence rate in High SDI countries

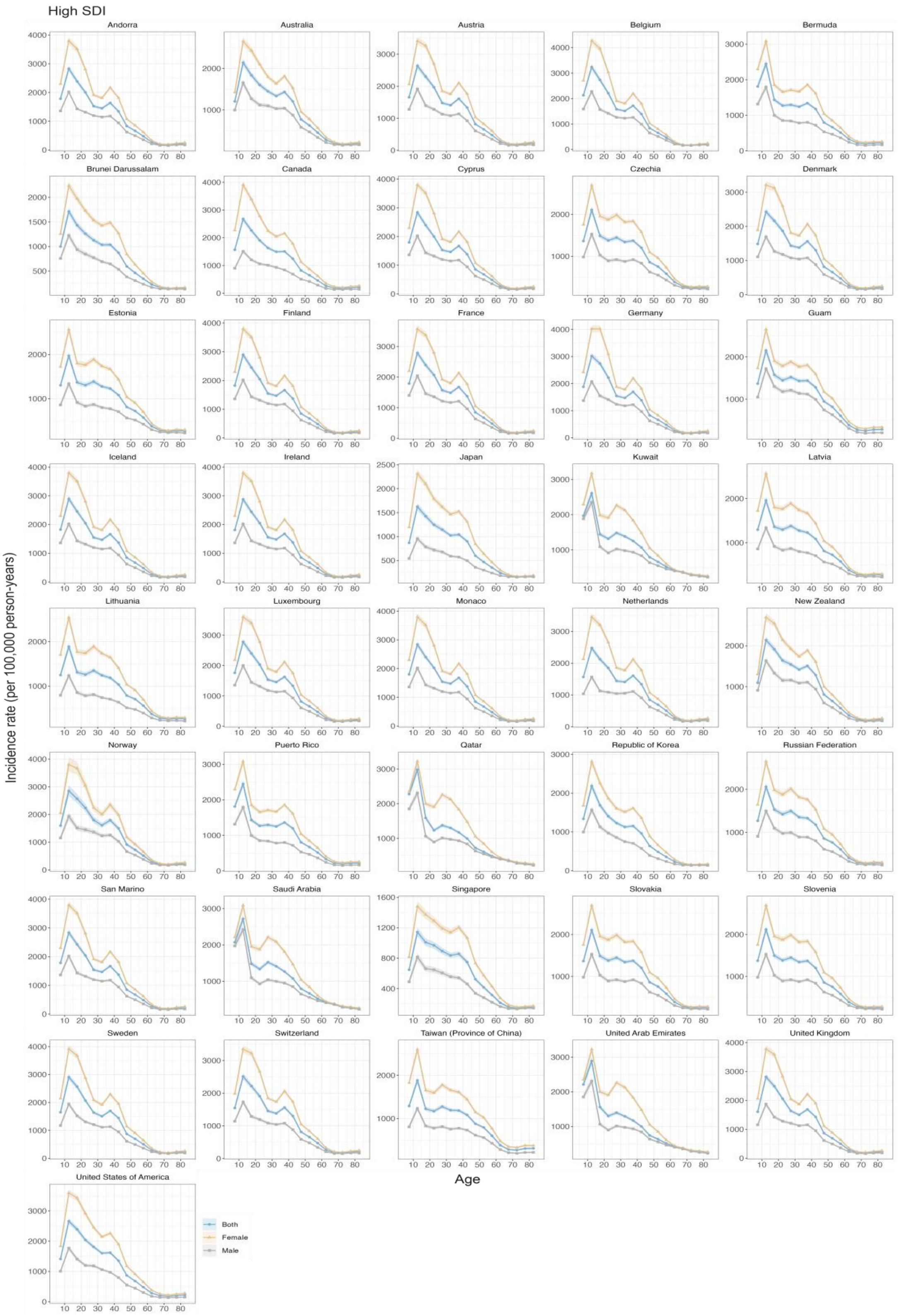

Age effects indicate age-associated natural history and are shown by the fitted longitudinal age curves of incidence rate (per 100000 person-years) adjusted for period deviations, with the dots and shaded areas denoting incidence rates with 95% CIs. SDI=Socio-demographic Index.

Figure 13 Age effects on migraine incidence rate in High-middle SDI countries

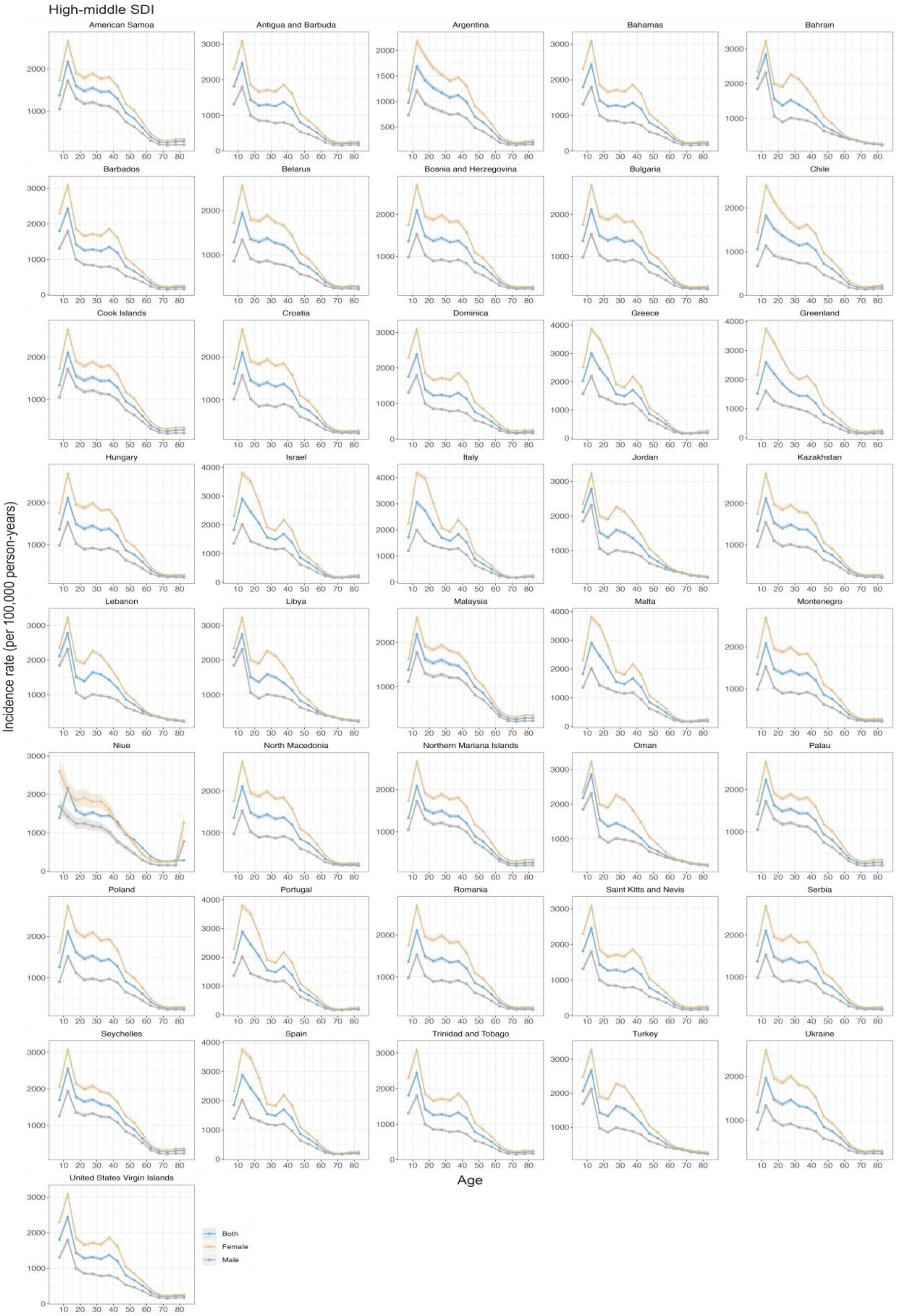

Age effects indicate age-associated natural history and are shown by the fitted longitudinal age curves of incidence rate (per 100000 person-years) adjusted for period deviations, with the dots and shaded areas denoting incidence rates with 95% CIs. SDI=Socio-demographic Index.

Figure 14 Age effects on migraine incidence rate in Middle SDI countries

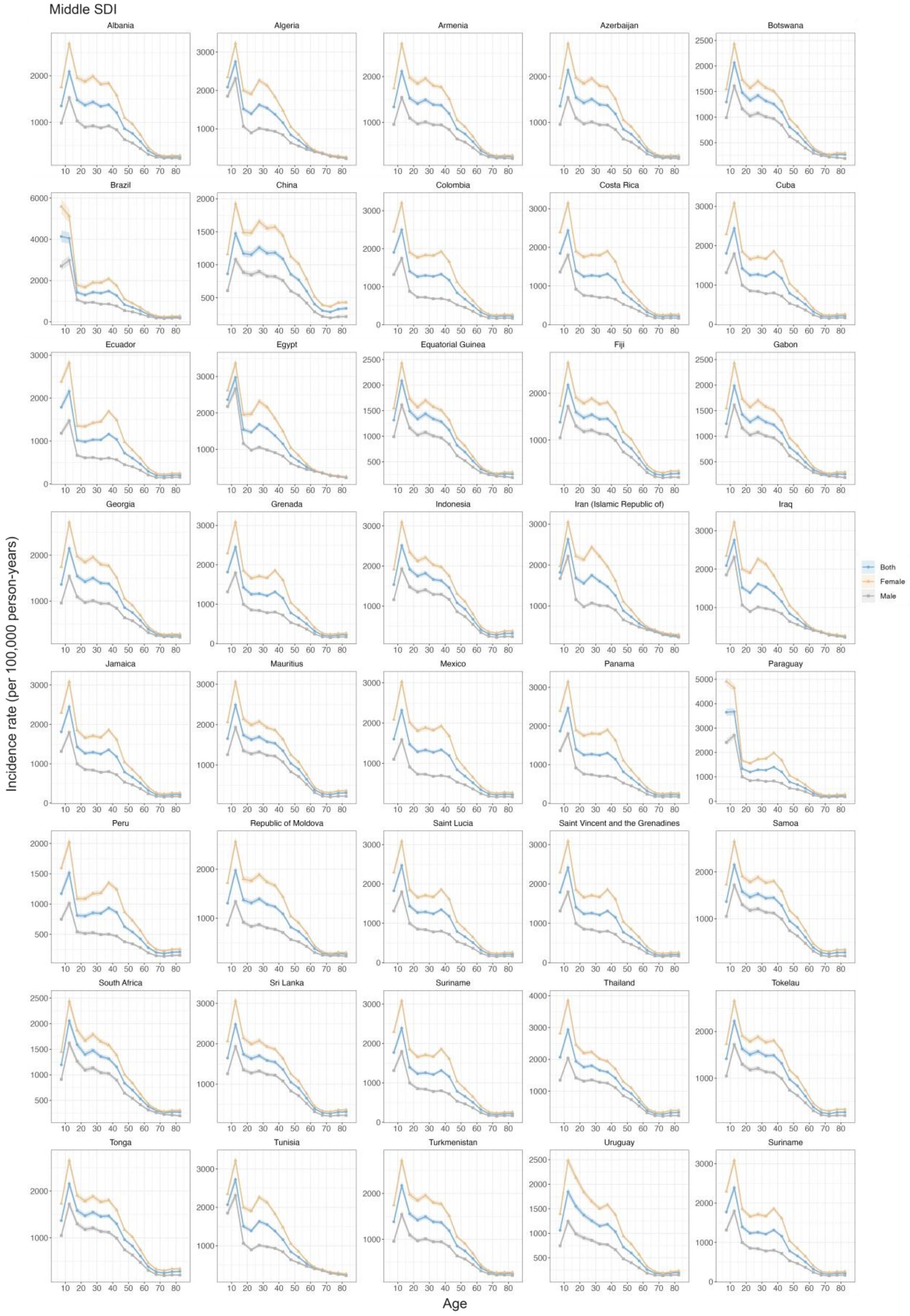

Age effects indicate age-associated natural history and are shown by the fitted longitudinal age curves of incidence rate (per 100000 person-years) adjusted for period deviations, with the dots and shaded areas denoting incidence rates with 95% CIs. SDI=Socio-demographic Index.

Figure 15 Age effects on migraine incidence rate in Low-middle SDI countries

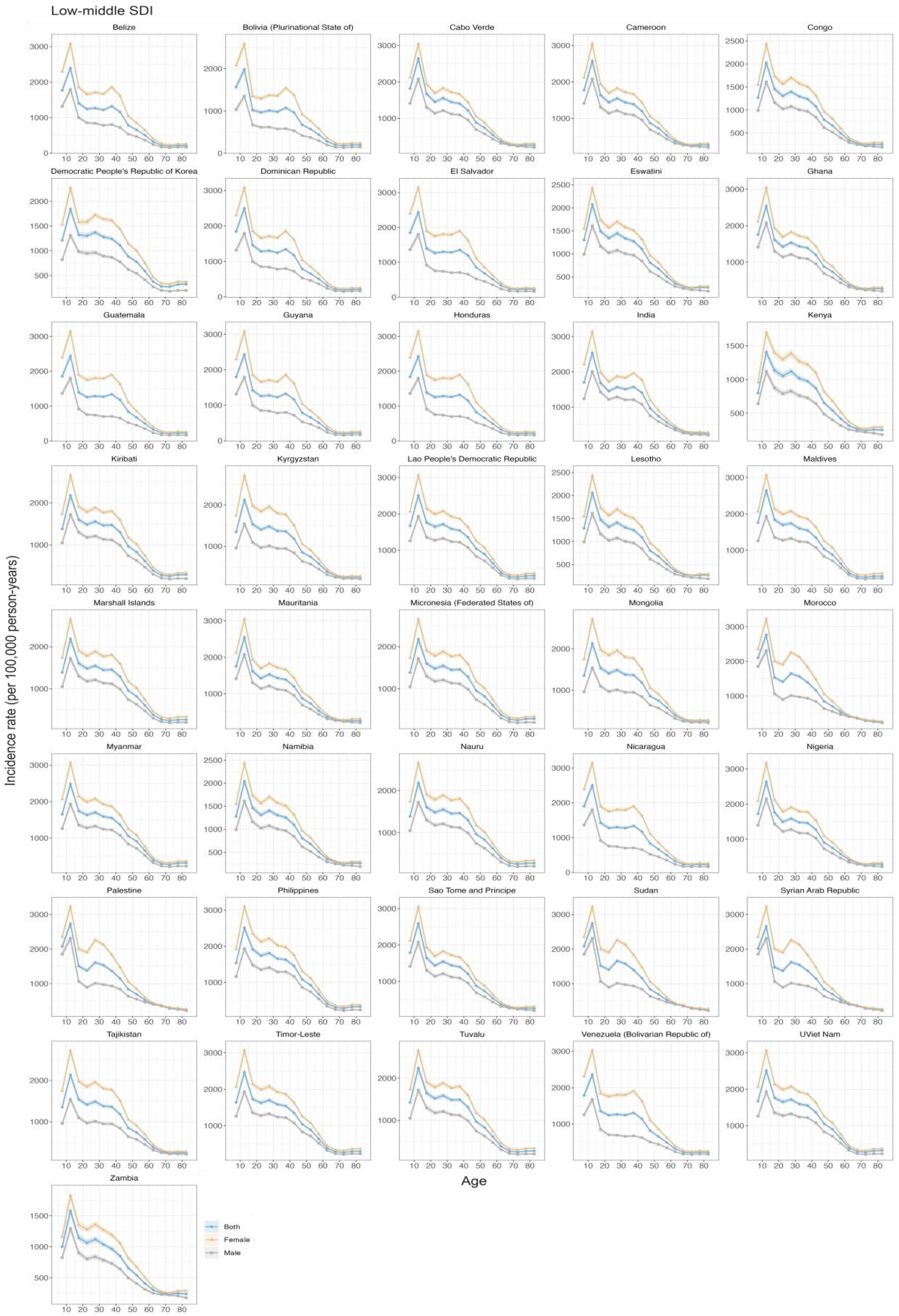

Age effects indicate age-associated natural history and are shown by the fitted longitudinal age curves of incidence rate (per 100000 person-years) adjusted for period deviations, with the dots and shaded areas denoting incidence rates with 95% CIs. SDI=Socio-demographic Index.

Figure 16 Age effects on migraine incidence rate in Low SDI countries

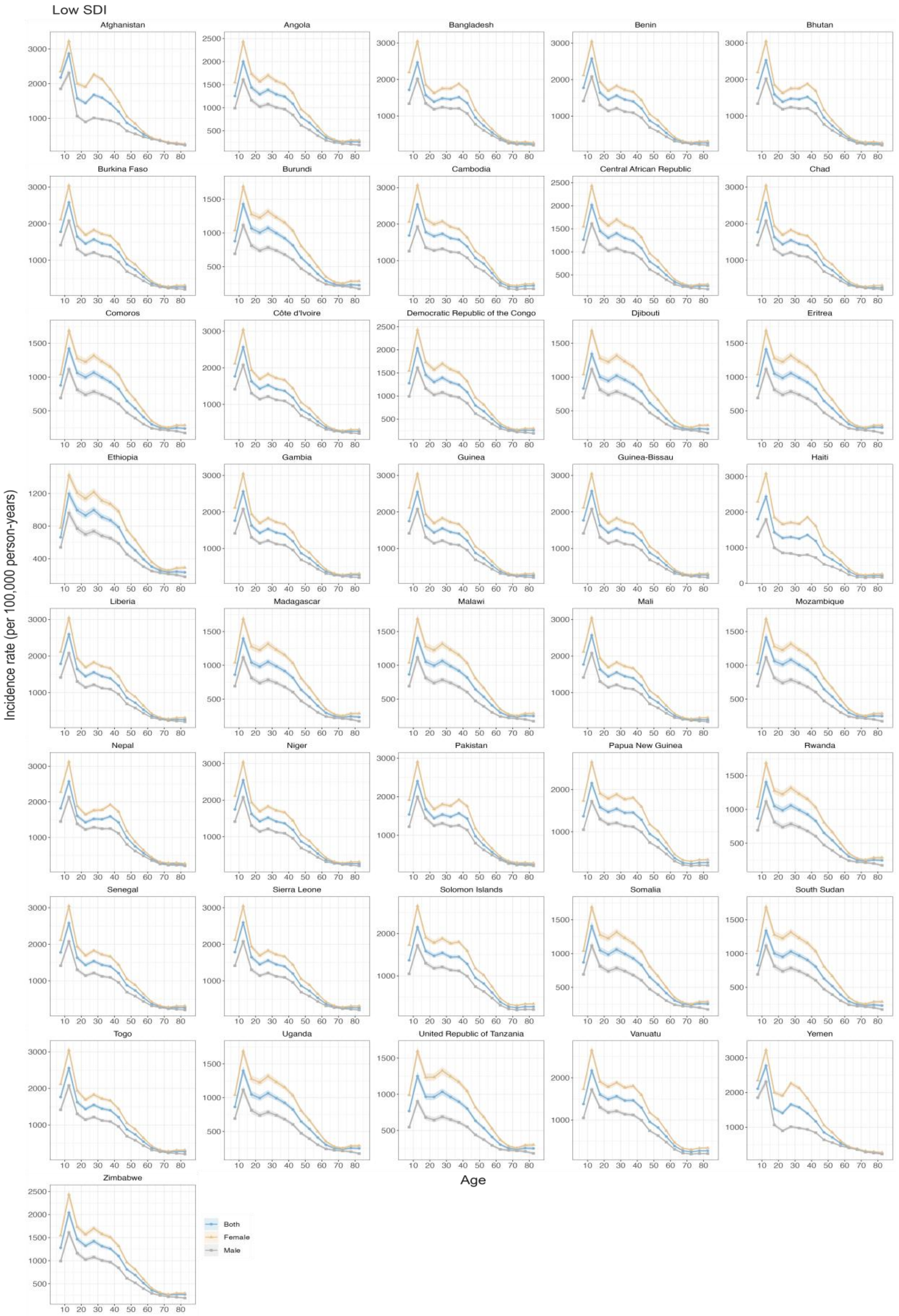

Age effects indicate age-associated natural history and are shown by the fitted longitudinal age curves of incidence rate (per 100000 person-years) adjusted for period deviations, with the dots and shaded areas denoting incidence rates with 95% CIs. SDI=Socio-demographic Index.

Figure 17 Period effects on migraine incidence rate in High SDI countries

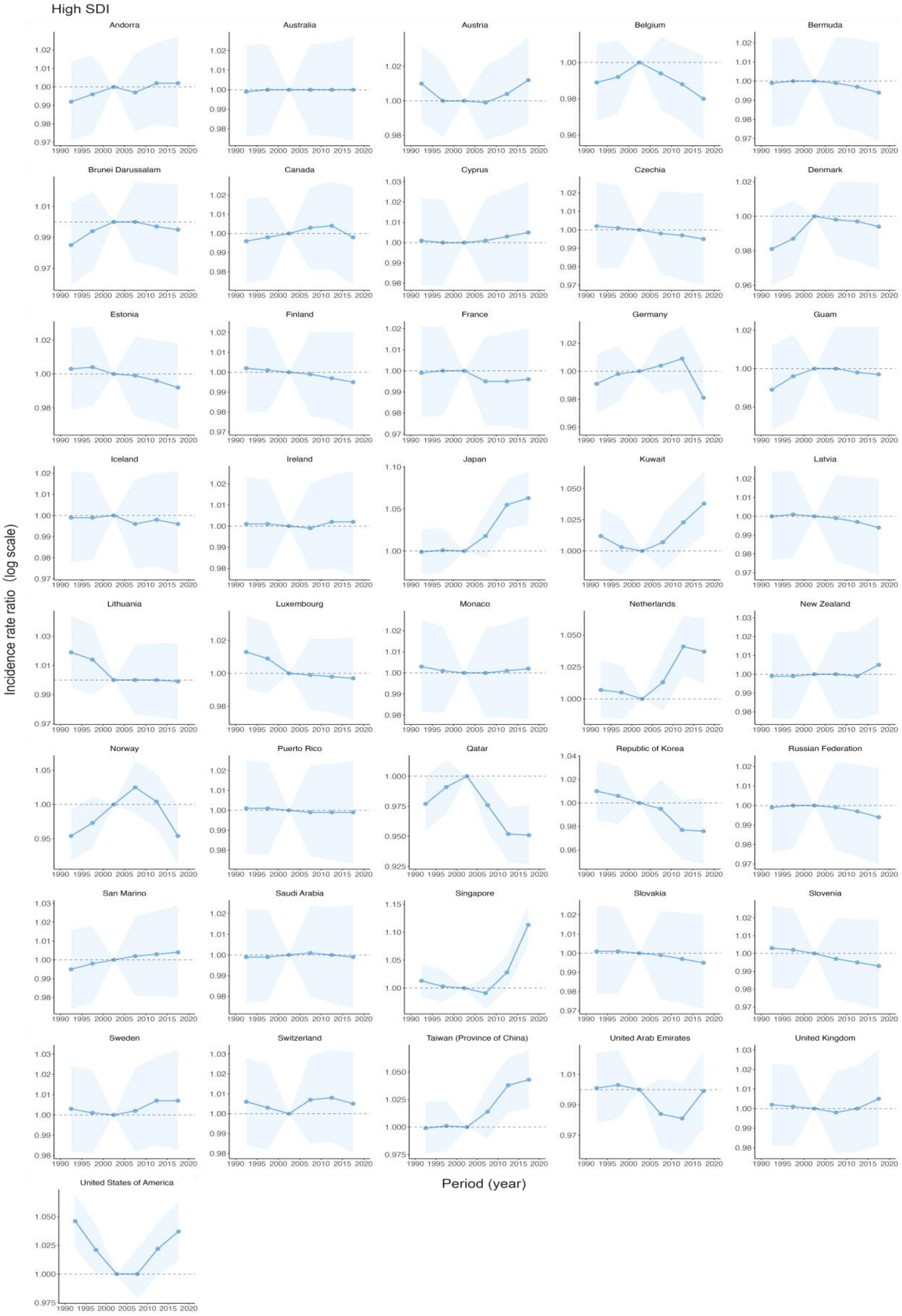

Period effects are shown by the relative risk of incidence rate (incidence rate ratio) for each period from 1990-1994 to 2015-2019, with the dots and shaded areas representing rate ratios and 95% CIs for a given period relative to the referent period (2000-2004). SDI=Socio-demographic Index.

Figure 18 Period effects on migraine incidence rate in High-middle SDI countries

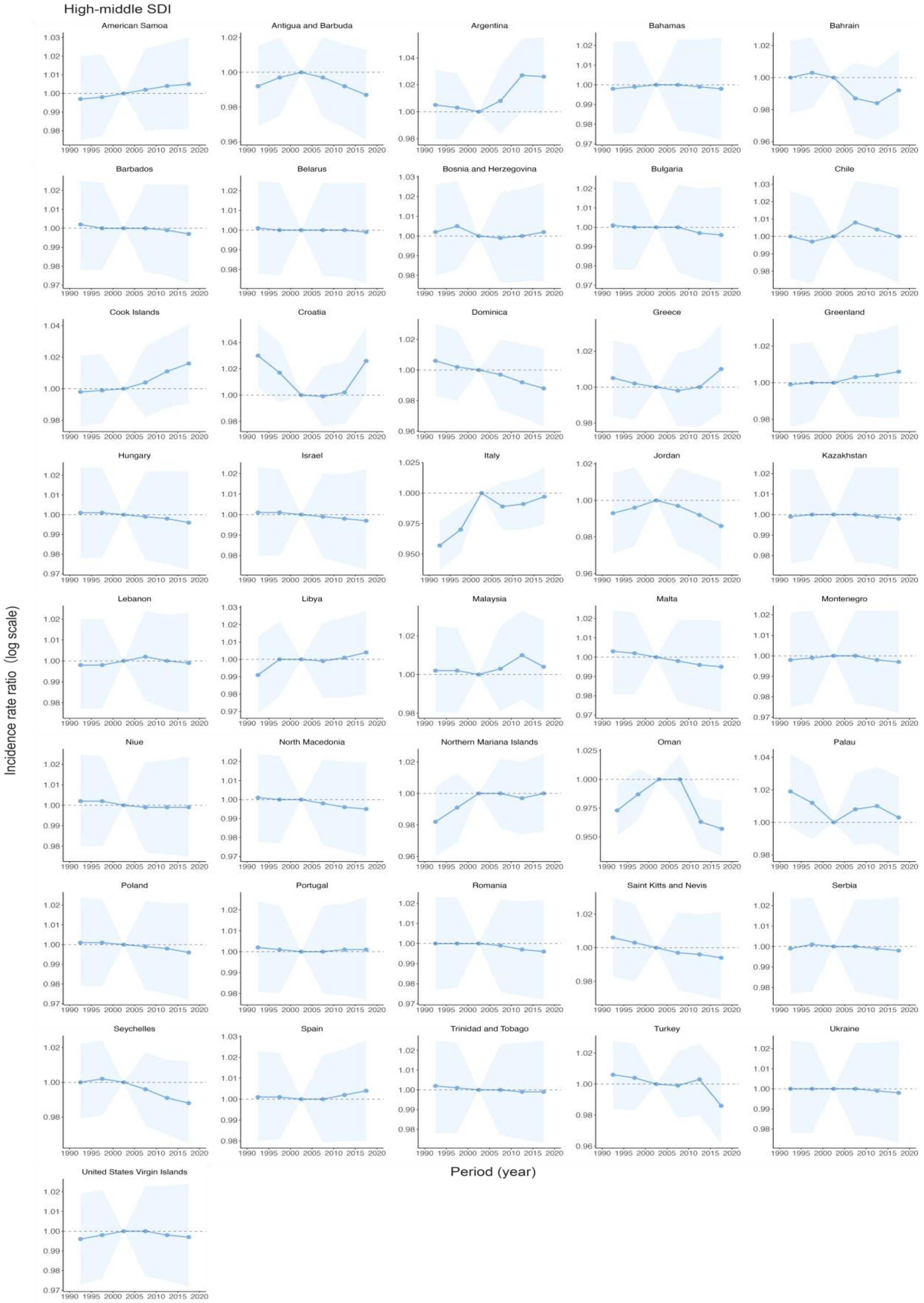

Period effects are shown by the relative risk of incidence rate (incidence rate ratio) for each period from 1990-1994 to 2015-2019, with the dots and shaded areas representing rate ratios and 95% CIs for a given period relative to the referent period (2000-2004). SDI=Socio-demographic Index.

Figure 19 Period effects on migraine incidence rate in Middle SDI countries

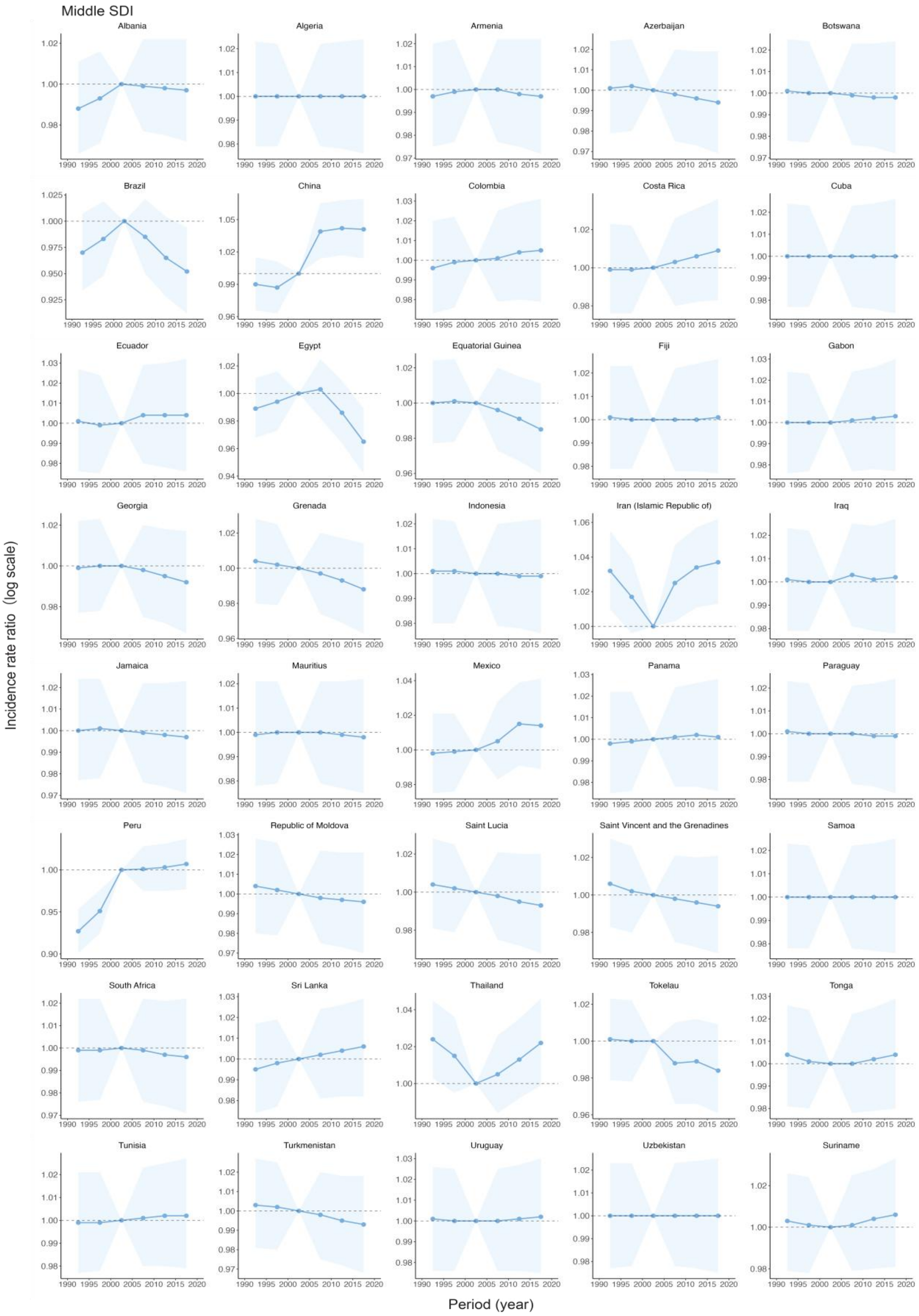

Period effects are shown by the relative risk of incidence rate (incidence rate ratio) for each period from 1990-1994 to 2015-2019, with the dots and shaded areas representing rate ratios and 95% CIs for a given period relative to the referent period (2000-2004). SDI=Socio-demographic Index.

Figure 20 Period effects on migraine incidence rate in Low-middle SDI countries

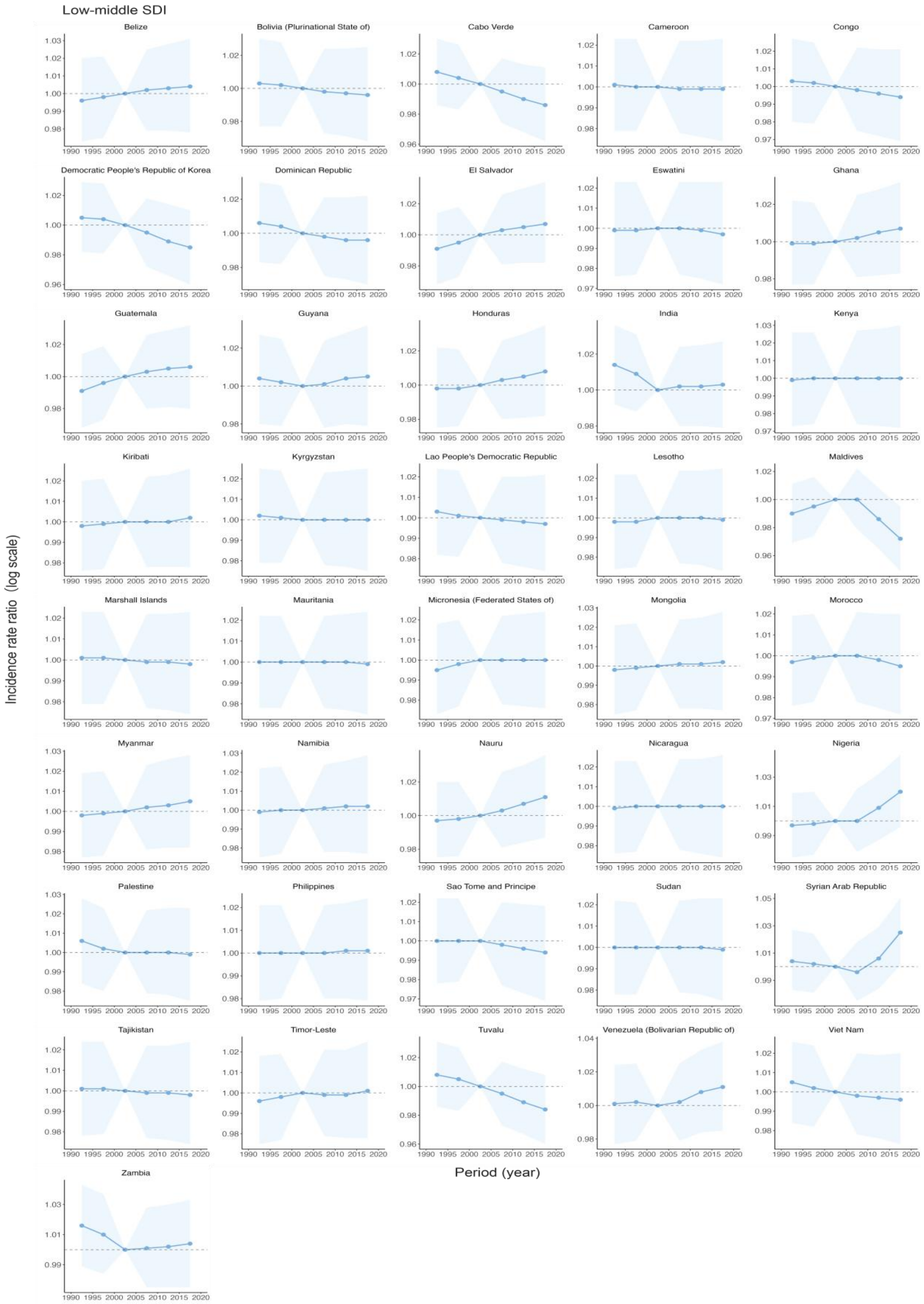

Period effects are shown by the relative risk of incidence rate (incidence rate ratio) for each period from 1990-1994 to 2015-2019, with the dots and shaded areas representing rate ratios and 95% CIs for a given period relative to the referent period (2000-2004). SDI=Socio-demographic Index.

Figure 21 Period effects on migraine incidence rate in Low SDI countries

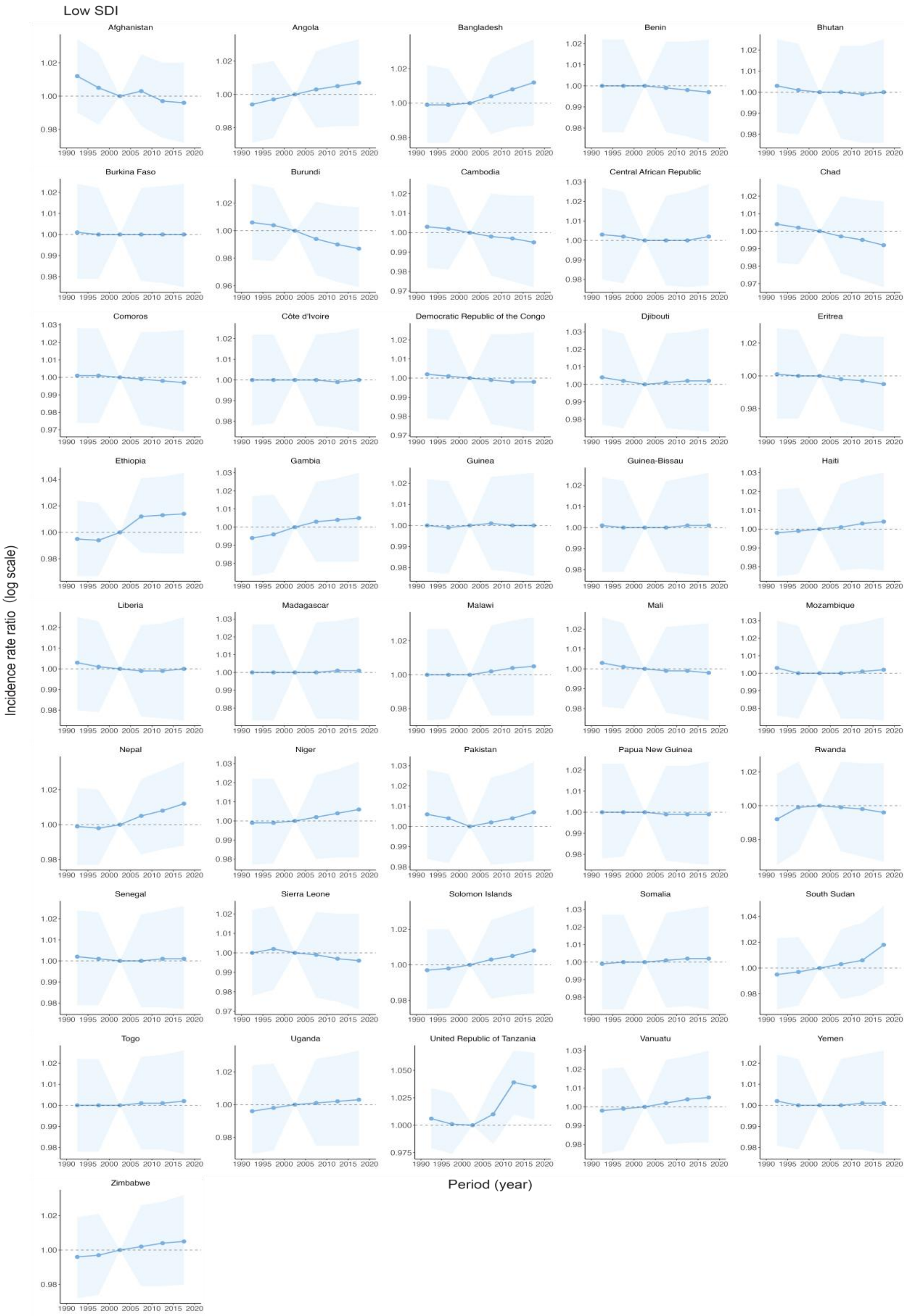

Period effects are shown by the relative risk of incidence rate (incidence rate ratio) for each period from 1990-1994 to 2015-2019, with the dots and shaded areas representing rate ratios and 95% CIs for a given period relative to the referent period (2000-2004). SDI=Socio-demographic Index.

Figure 22 Cohort effects on migraine incidence rate in High SDI countries

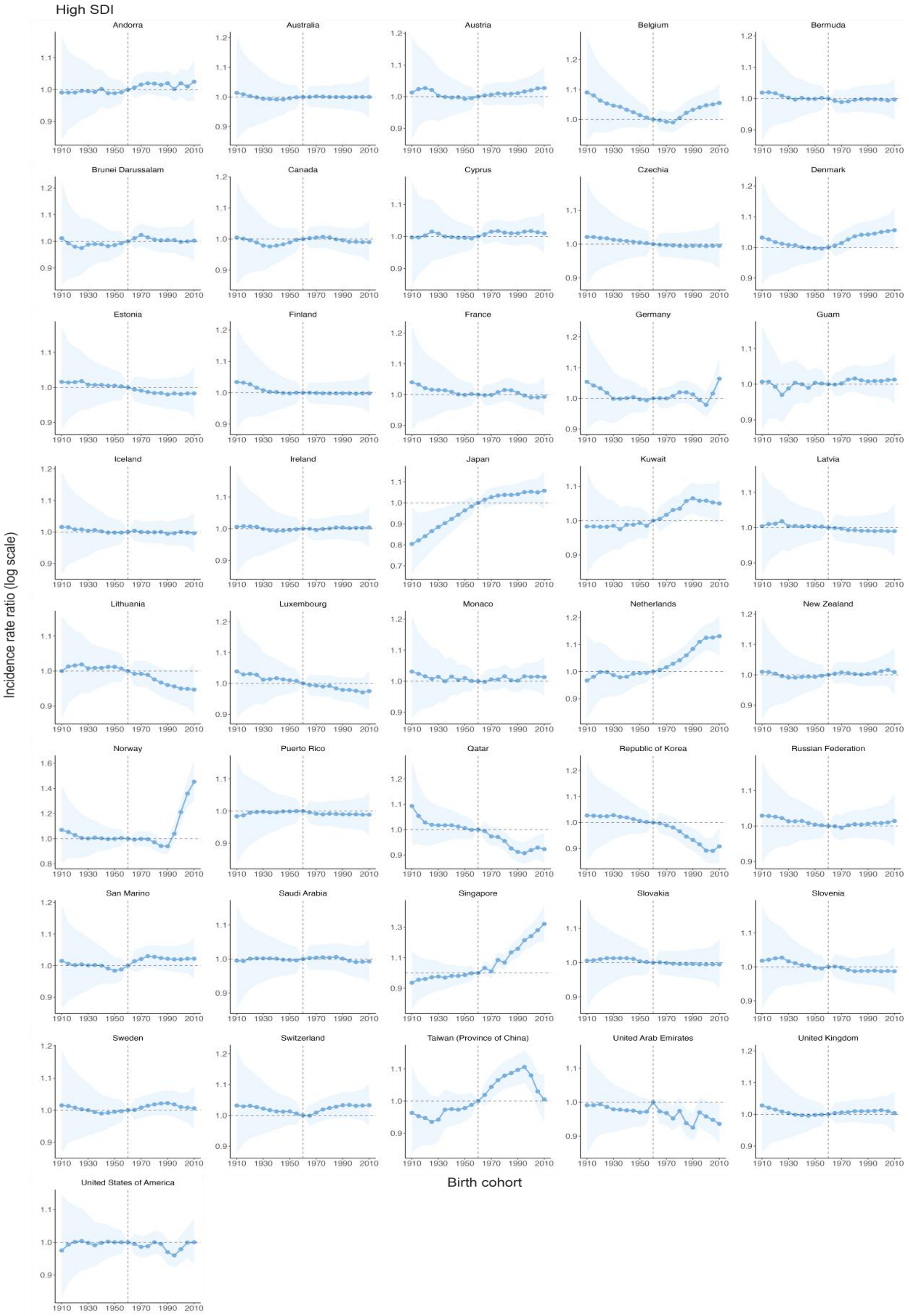

Cohort effects are shown by the relative risk of incidence rate (incidence rate ratio) for each birth cohort from 1910 to 2010, with the dots and shaded areas represent rate ratios and 95% CIs for a given cohort relative to the referent 1960 cohort. SDI=Socio-demographic Index.

Figure 23 Cohort effects on migraine incidence rate in High-middle SDI countries

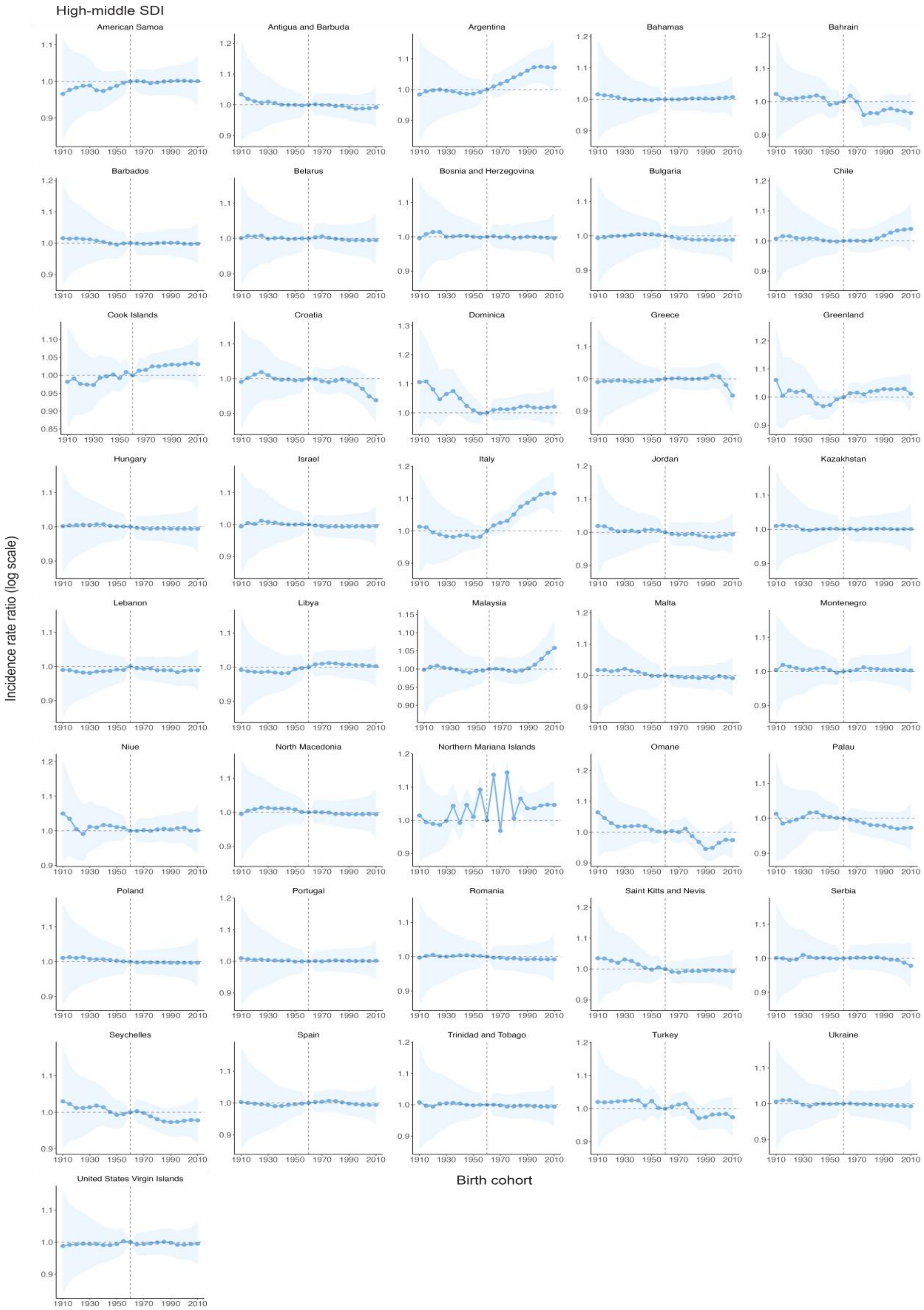

Cohort effects are shown by the relative risk of incidence rate (incidence rate ratio) for each birth cohort from 1910 to 2010, with the dots and shaded areas represent rate ratios and 95% CIs for a given cohort relative to the referent 1960 cohort. SDI=Socio-demographic Index.

Figure 24 Cohort effects on migraine incidence rate in Middle SDI countries

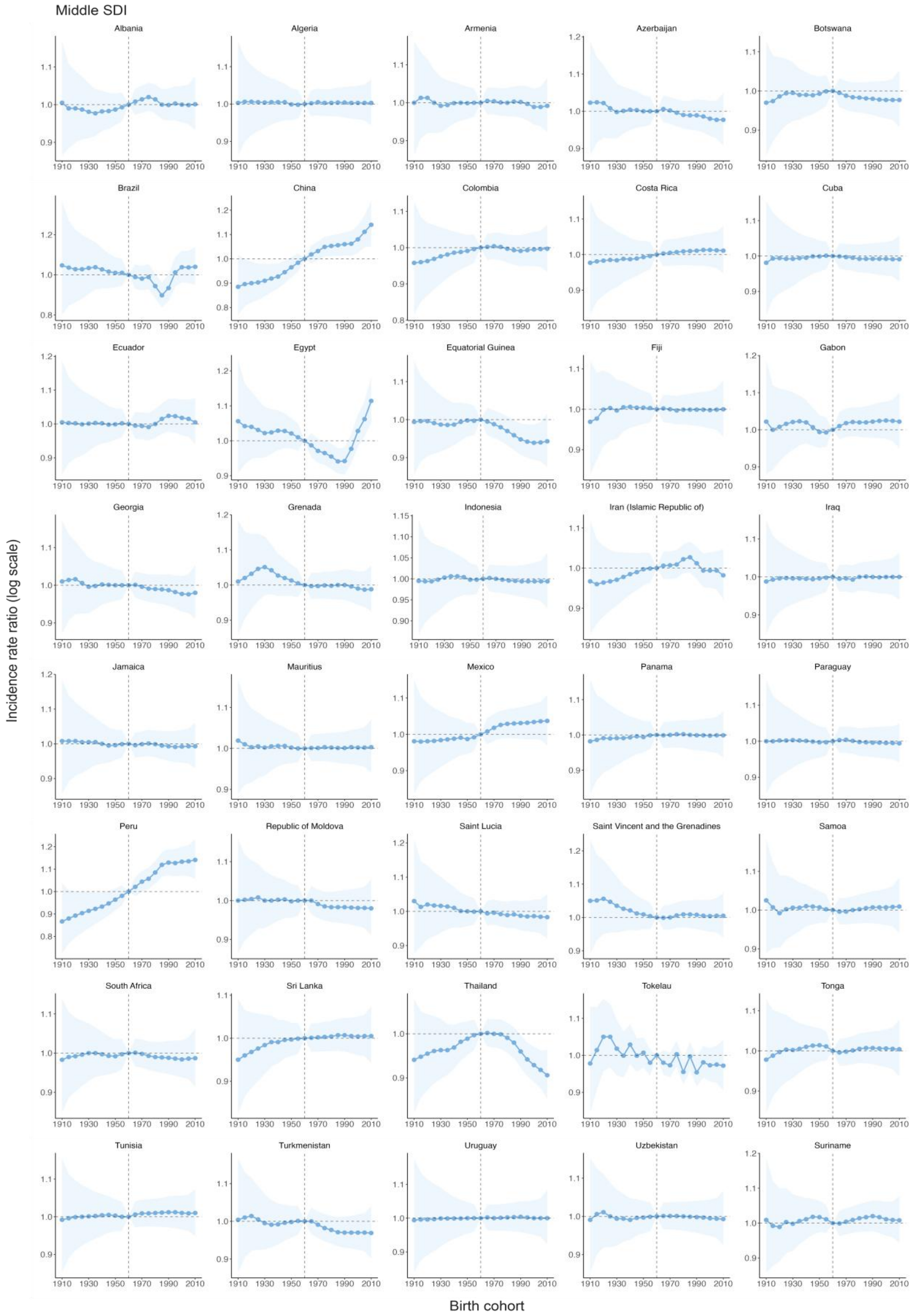

Cohort effects are shown by the relative risk of incidence rate (incidence rate ratio) for each birth cohort from 1910 to 2010, with the dots and shaded areas represent rate ratios and 95% CIs for a given cohort relative to the referent 1960 cohort. SDI=Socio-demographic Index.

Figure 25 Cohort effects on migraine incidence rate in Low-middle SDI countries

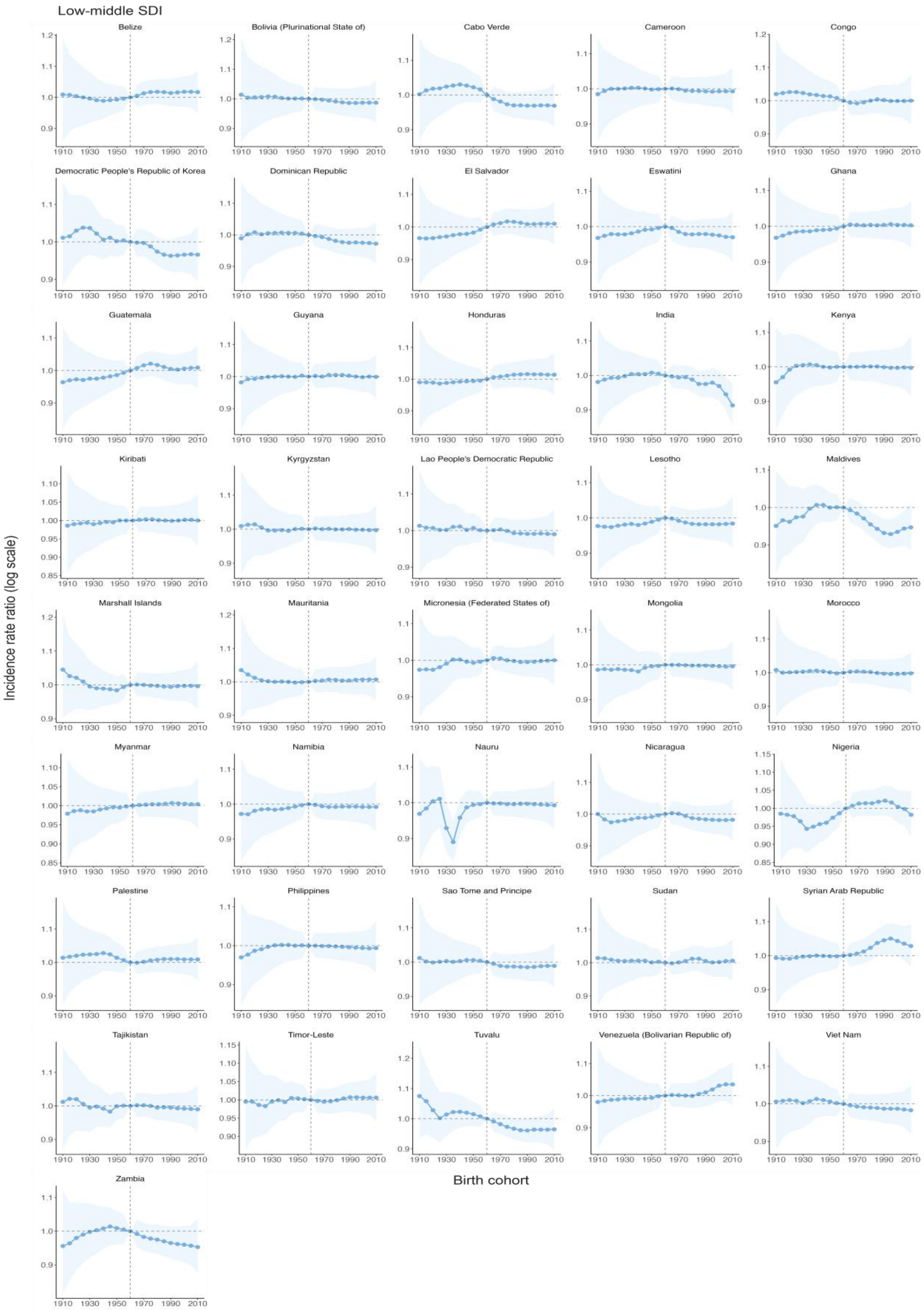

Cohort effects are shown by the relative risk of incidence rate (incidence rate ratio) for each birth cohort from 1910 to 2010, with the dots and shaded areas represent rate ratios and 95% CIs for a given cohort relative to the referent 1960 cohort. SDI=Socio-demographic Index.

Figure 26 Cohort effects on migraine incidence rate in Low SDI countries

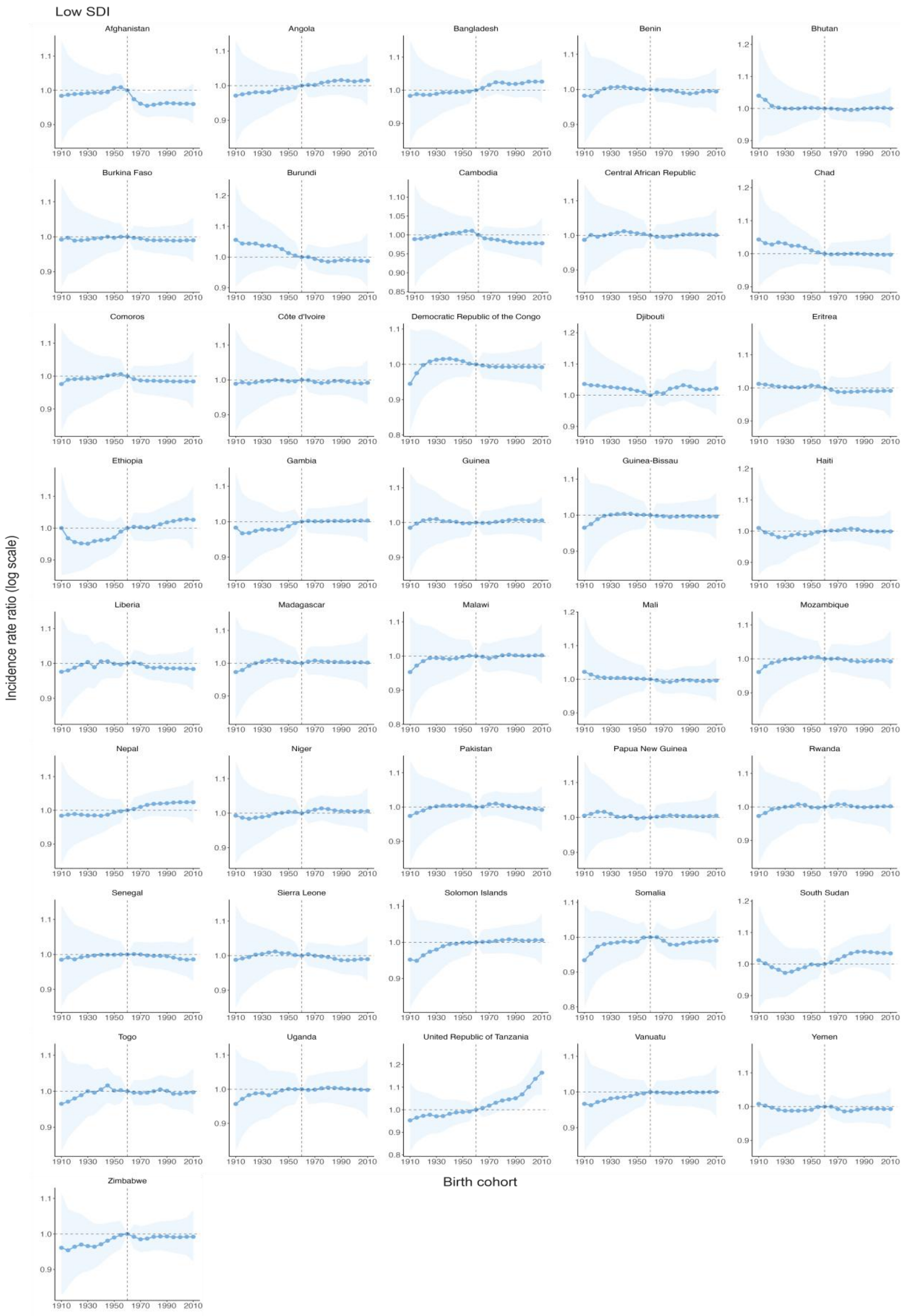

Cohort effects are shown by the relative risk of incidence rate (incidence rate ratio) for each birth cohort from 1910 to 2010, with the dots and shaded areas represent rate ratios and 95% CIs for a given cohort relative to the referent 1960 cohort. SDI=Socio-demographic Index

Figure 27 Favorable (A) and unfavorable (B) age-period-cohort effects on exemplary countries across SDI quintiles

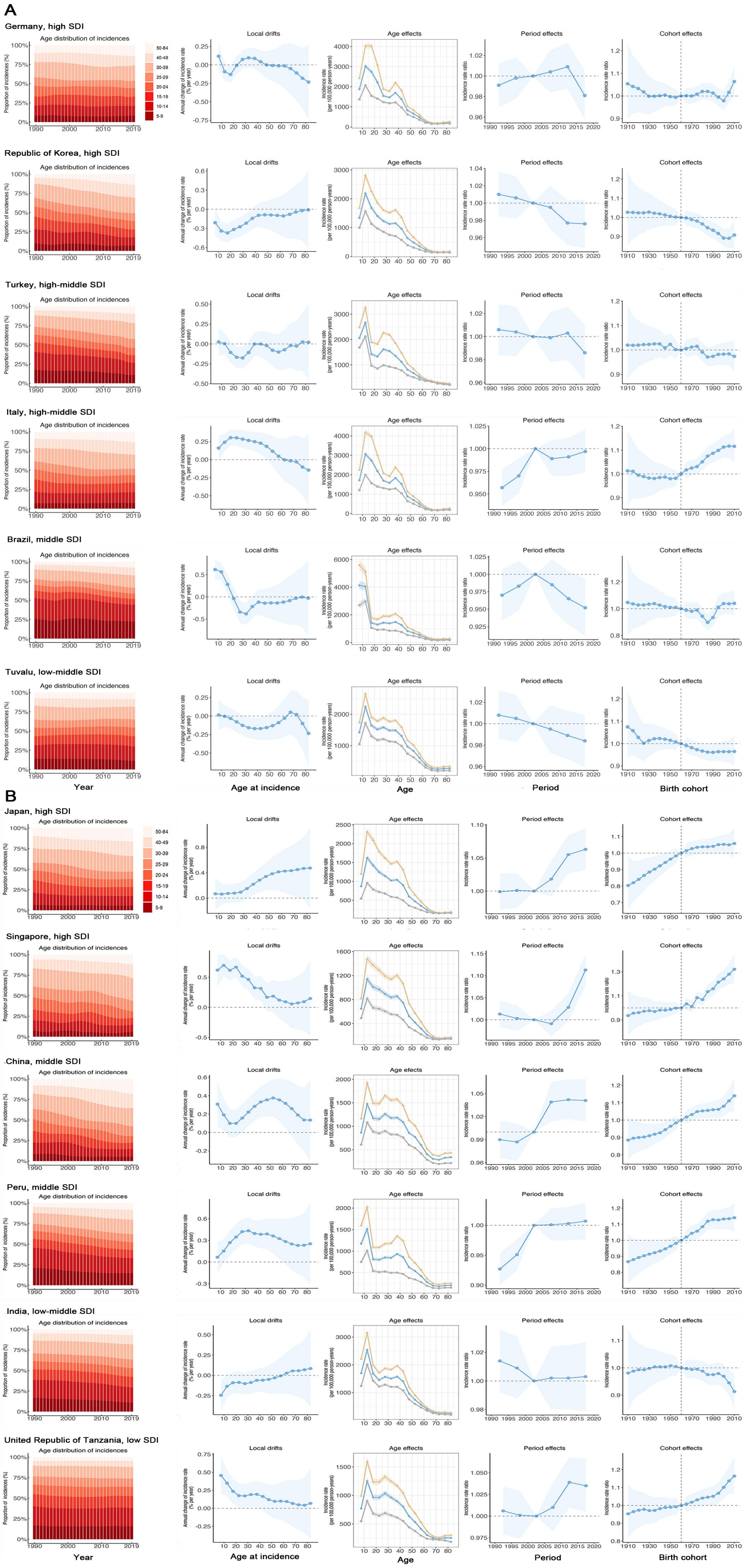

Age distribution of incidences shows the relative proportion of incidences from each age group during 1990-2019. Local drifts indicate the annual percentage change of incidence rate (% per year) across five-year age groups (from 5-9 to 80-84 years). Age effects are represented by the fitted longitudinal age curves of incidence rate (per 100,000 person-years) adjusted for period deviations. Period effects are represented by the relative risk of incidence rate (incidence rate ratio) and computed as the ratio of age-specific rates in each period compared to the referent 2000-2004 period. Cohort effects are represented by the relative risk of incidence rate (incidence rate ratio) and computed as the ratio of age-specific rates in each cohort compared to referent 1960 cohort. The shaded areas indicate the corresponding 95% CIs of each point estimate. SDI: Socio-demographic Index.

Table 1 The lexis diagram of GBD data for the APC model

	Age groups																
Period (median)	5-9	10-14	15-19	20-24	25-29	30-34	35-39	40-44	45-49	50-54	55-59	60-64	65-69	70-74	75-79	80-84	Birth cohort (median)
																X	1906-1914 (1910)
															X	X	1911-1919 (1915)
														X	X	X	1916-1924 (1920)
													X	X	X	X	1921-1929 (1925)
												X	X	X	X	X	1926-1934 (1930)
											X	X	X	X	X	X	1931-1939 (1935)
										X	X	X	X	X	X		1936-1944 (1940)
									X	X	X	X	X	X			1941-1949 (1945)
								X	X	X	X	X	X				1946-1954 (1950)
							X	X	X	X	X	X					1951-1959 (1955)
						X	X	X	X	X	X						1956-1964 (1960)
					X	X	X	X	X	X							1961-1969 (1965)
				X	X	X	X	X	X								1966-1974 (1970)
			X	X	X	X	X	X									1971-1979 (1975)
		X	X	X	X	X	X										1976-1984 (1980)
	X	X	X	X	X	X											1981-1989 (1985)
1990-1994 (1992)	X	X	X	X	X												1986-1994 (1990)
1995-1999 (1997)	X	X	X	X													1991-1999 (1995)
2000-2004 (2002)	X	X	X														1996-2004 (2000)
2005-2009 (2007)	X	X															2001-2009 (2005)
2010-2014 (2012)	X																2006-2014 (2010)
2015-2019 (2017)																	

Note: X denotes incidence rate data of each age group from the corresponding period. For instance, the mortality rate of age 5-9 years in 1992 is filled in the square with a bold X (see table), and this square belongs to the cohort 1981-1989 (1985).

Table 2 Time trends in migraine incidence rate for both sexes in 204 countries, 1990-2019

SDI		Population		Incidence		All-age incidence rate		Age-standardized incidence rate		Net drift of incidence rate from APC model, % per year
**Quintil**	**Country**	**Number in 2019**	**Percent change 1990-2019, %**	**Number in 2019**	**Percent change 1990-2019, %**	**Rate in 2019**	**Percent change 1990-2019, %**	**Rate in 2019**	**Percent change 1990-2019, %**	
High SDI	Andorra	55505 (48383, 62764)	14.61	933 (807, 1085)	28.12 (21.48, 35.46)	1123.74 (971.95, 1306.02)	-16.57 (-20.89, -11.79)	1345.46 (1148.65, 1551.91)	0.79 (0.61, 1.03)	0.037 (-0.062, 0.137)
High SDI	Australia	24568113 (22510058, 26779186)	45.72	236212 (202547, 270589)	30.01 (27.54, 32.8)	961.46 (824.43, 1101.38)	-10.78 (-12.48, -8.87)	1085.69 (917.45, 1248.01)	0.11 (0.06, 0.17)	0.004 (-0.099, 0.107)
High SDI	Austria	8916185 (8169639, 9666456)	14.77	93754 (80864, 107309)	1.27 (-1.4, 4.09)	1051.51 (906.93, 1203.53)	-11.76 (-14.09, -9.31)	1301.24 (1107.33, 1497.59)	-0.26 (-0.35, -0.19)	0.009 (-0.09, 0.109)
High SDI	Belgium	11419166 (10536911, 12317981)	14.43	136877 (120022, 155991)	5.99 (1.79, 10.26)	1198.66 (1051.06, 1366.05)	-7.38 (-11.04, -3.64)	1493.92 (1287.92, 1703.75)	2.83 (-0.86, 7.09)	-0.035 (-0.134, 0.063)
High SDI	Bermuda	64030 (58254, 69664)	7.7	563 (490, 647)	-10.66 (-14.24, -6.26)	879.3 (765.01, 1010.9)	-17.05 (-20.37, -12.96)	1087.39 (921.08, 1255.44)	-0.11 (-0.19, -0.02)	-0.018 (-0.117, 0.081)
High SDI	Brunei Darussalam	437119 (381966, 491691)	69.07	3932 (3323, 4563)	61.43 (54.57, 68.78)	899.63 (760.17, 1043.96)	-4.52 (-8.58, -0.17)	844.88 (710.4, 978.24)	0.14 (-0.06, 0.35)	0.033 (-0.083, 0.149)
High SDI	Canada	36519840 (33331463, 39599796)	33.98	380529 (327446, 435521)	13.88 (8.6, 19.09)	1041.98 (896.62, 1192.56)	-15 (-18.95, -11.11)	1252.15 (1056.68, 1439.9)	-0.82 (-4.96, 3.47)	0.018 (-0.083, 0.12)
High SDI	Cyprus	10643487 (9779074, 11500126)	3.36	97144 (84095, 110594)	-10.07 (-13.44, -6.73)	912.71 (790.11, 1039.07)	-13 (-16.26, -9.77)	1070.05 (916.53, 1223.84)	-0.39 (-0.47, -0.33)	0.018 (-0.081, 0.117)
High SDI	Czechia	26171532 (23573125, 28872979)	114.06	336102 (279179, 396490)	116.8 (111.91, 121.56)	1284.23 (1066.73, 1514.97)	1.28 (-1, 3.51)	1169.88 (998.1, 1355.47)	-0.16 (-0.27, -0.05)	-0.031 (-0.123, 0.062)
High SDI	Denmark	5802733 (5329985, 6262192)	12.8	60529 (51883, 69416)	4.68 (0.62, 8.91)	1043.11 (894.11, 1196.26)	-7.2 (-10.8, -3.45)	1251.85 (1058.75, 1436.99)	2.37 (-1.53, 6.34)	0.051 (-0.049, 0.152)
High SDI	Estonia	1312361 (1204372, 1415452)	-16.34	11277 (9771, 12877)	-24.72 (-26.48, -22.76)	859.28 (744.52, 981.2)	-10.01 (-12.12, -7.67)	995.03 (847.8, 1148.2)	-0.78 (-0.94, -0.63)	-0.046 (-0.14, 0.048)
High SDI	Finland	5534095 (5086541, 5991975)	10.46	59574 (51463, 68306)	-3.92 (-5.41, -2.32)	1076.49 (929.93, 1234.27)	-13.02 (-14.36, -11.57)	1348.59 (1150.38, 1555.71)	-0.17 (-0.2, -0.14)	-0.024 (-0.122, 0.075)
High SDI	France	66204315 (60093850, 72433669)	14.6	731883 (634332, 836344)	0.71 (-3.27, 4.68)	1105.49 (958.14, 1263.28)	-12.12 (-15.59, -8.65)	1333.37 (1138.2, 1526.8)	-0.86 (-4.4, 2.5)	-0.021 (-0.12, 0.077)
High SDI	Germany	84914056 (77688648, 92219489)	6.22	912665 (788353, 1046772)	-5.48 (-9.32, -0.44)	1074.81 (928.41, 1232.74)	-11.02 (-14.63, -6.27)	1411.51 (1213.35, 1626.29)	1.52 (-2.17, 6.49)	-0.005 (-0.104, 0.094)
High SDI	Guam	170628 (149082, 191543)	24.76	1872 (1597, 2154)	14.44 (11.2, 18.3)	1097.35 (935.84, 1262.29)	-8.28 (-10.87, -5.18)	1137.96 (967.04, 1309.37)	0.61 (0.45, 0.79)	0.027 (-0.064, 0.118)
High SDI	Iceland	344876 (316885, 373204)	35.79	4129 (3559, 4742)	20.45 (18.09, 23.12)	1197.2 (1031.9, 1374.94)	-11.3 (-13.04, -9.34)	1348.42 (1150.08, 1555.62)	-0.13 (-0.23, -0.05)	-0.013 (-0.112, 0.086)
High SDI	Ireland	4910357 (4483761, 5355859)	36.35	59797 (51579, 68933)	20 (15.73, 24.73)	1217.78 (1050.41, 1403.83)	-11.99 (-15.13, -8.53)	1356.11 (1159.1, 1563.93)	0.31 (0.23, 0.41)	0.003 (-0.096, 0.102)
High SDI	Japan	127788411 (115774123, 139878471)	1.53	831946 (735116, 929478)	-17.72 (-21.01, -14.15)	651.03 (575.26, 727.36)	-18.96 (-22.2, -15.44)	867.42 (753.89, 977.98)	2.89 (-0.57, 6.23)	0.28 (0.164, 0.397)
High SDI	Kuwait	4426561 (3926462, 4929515)	151.59	56016 (47909, 65273)	140.78 (123.86, 159.65)	1265.45 (1082.3, 1474.58)	-4.3 (-11.02, 3.21)	1223.61 (1039.83, 1415.39)	2.83 (-0.51, 6.59)	0.111 (0.018, 0.203)
High SDI	Latvia	1915292 (1760157, 2071422)	-27.96	16155 (14034, 18478)	-35.59 (-37.23, -33.71)	843.5 (732.71, 964.76)	-10.59 (-12.86, -7.98)	998.19 (850.38, 1152.15)	-0.59 (-0.68, -0.51)	-0.027 (-0.121, 0.067)
High SDI	Lithuania	2794223 (2574822, 3025990)	-23.94	22490 (19531, 25575)	-34.9 (-38.11, -31.16)	804.86 (698.96, 915.27)	-14.41 (-18.64, -9.5)	943.84 (804.33, 1080.57)	-2.79 (-6.76, 1.3)	-0.081 (-0.176, 0.014)
High SDI	Luxembourg	618550 (568118, 666377)	62.24	7040 (6122, 8078)	54.71 (51.88, 57.49)	1138.12 (989.71, 1305.94)	-4.64 (-6.39, -2.93)	1315.07 (1120.23, 1513.54)	-0.13 (-0.2, -0.05)	-0.065 (-0.165, 0.034)
High SDI	Monaco	37572 (34251, 40757)	23.43	366 (317, 421)	16.52 (13.29, 20.19)	974.02 (844.9, 1119.38)	-5.6 (-8.22, -2.62)	1356.96 (1159.83, 1564.13)	0.35 (0.15, 0.58)	-0.004 (-0.102, 0.095)
High SDI	Netherlands	17156788 (15675163, 18613311)	14.97	186362 (160990, 213622)	3.97 (-0.5, 8.75)	1086.23 (938.35, 1245.12)	-9.56 (-13.45, -5.4)	1332.75 (1128.46, 1536.61)	5.18 (0.03, 9.7)	0.151 (0.052, 0.25)
High SDI	New Zealand	4495667 (4005504, 4968118)	31.56	44331 (38919, 49392)	15.55 (13.51, 17.88)	986.08 (865.7, 1098.66)	-12.17 (-13.72, -10.4)	1126.16 (987.71, 1257.1)	0.33 (0.24, 0.43)	0.017 (-0.084, 0.117)
High SDI	Norway	5348847 (4936723, 5754787)	25.95	68369 (60773, 76138)	27.97 (23.3, 33.04)	1278.2 (1136.19, 1423.45)	1.61 (-2.1, 5.63)	1515.74 (1333.82, 1693.42)	12.15 (8.25, 16.3)	0.065 (-0.112, 0.243)
High SDI	Puerto Rico	3521431 (3106914, 3986032)	-2.54	32667 (28264, 37231)	-19.04 (-22.33, -15.29)	927.67 (802.62, 1057.26)	-16.92 (-20.31, -13.07)	1090.49 (923.58, 1258.93)	-0.32 (-0.38, -0.28)	-0.009 (-0.108, 0.09)
High SDI	Qatar	2864548 (2595390, 3123704)	543.53	32281 (27198, 37927)	494.08 (455.97, 527.07)	1126.93 (949.48, 1324.03)	-7.69 (-13.61, -2.56)	1106.23 (930.64, 1292.44)	-2.31 (-3.01, -1.63)	-0.161 (-0.256, -0.066)
High SDI	Republic of Korea	53398252 (48441017, 58407132)	20.44	408451 (352196, 469025)	-14.03 (-20.44, -6.83)	764.91 (659.56, 878.35)	-28.62 (-33.94, -22.64)	912.3 (780.55, 1049.65)	-5.56 (-10.63, -1.16)	-0.151 (-0.264, -0.039)
High SDI	Russian Federation	146717427 (128850161, 165171807)	-2.85	1416866 (1258455, 1582178)	-10.63 (-12.72, -8.22)	965.71 (857.74, 1078.38)	-8.01 (-10.16, -5.52)	1073.63 (941.88, 1202.44)	-0.1 (-0.13, -0.06)	-0.019 (-0.109, 0.072)
High SDI	San Marino	33100 (28891, 37191)	40.51	380 (329, 436)	25.7 (22.24, 29.6)	1147.2 (995.42, 1317.17)	-10.54 (-13.01, -7.77)	1366.18 (1169.4, 1574.5)	0.84 (0.5, 1.25)	0.038 (-0.06, 0.137)
High SDI	Saudi Arabia	35731972 (31175706, 40192039)	122.7	449325 (383954, 519673)	110.59 (89.88, 133.2)	1257.49 (1074.54, 1454.36)	-5.44 (-14.73, 4.72)	1179.24 (1004.51, 1362.18)	-0.65 (-3.9, 2.42)	-0.001 (-0.093, 0.092)
High SDI	Singapore	5667451 (5233147, 6058684)	85.99	40491 (34603, 46725)	76.17 (60.49, 91.13)	714.45 (610.56, 824.44)	-5.28 (-13.71, 2.77)	796.62 (670.74, 921.18)	16.04 (8.26, 24.54)	0.305 (0.184, 0.426)
High SDI	Slovakia	5437223 (4969934, 5923604)	2.92	51825 (45078, 59262)	-9.21 (-13.25, -5.07)	953.16 (829.06, 1089.94)	-11.78 (-15.71, -7.77)	1071.19 (917.64, 1224.99)	-0.42 (-0.51, -0.36)	-0.025 (-0.118, 0.067)
High SDI	Slovenia	2074271 (1914446, 2243231)	5.24	18539 (16168, 21175)	-11.1 (-14.29, -7.54)	893.78 (779.47, 1020.84)	-15.52 (-18.56, -12.14)	1067.08 (913.42, 1220.66)	-0.56 (-0.65, -0.47)	-0.043 (-0.136, 0.05)
High SDI	Sweden	10222546 (9312312, 11127453)	19.02	116580 (103596, 130185)	13.07 (9.62, 16.2)	1140.42 (1013.4, 1273.51)	-5 (-7.9, -2.37)	1384.69 (1218.43, 1547.07)	0.2 (-2.25, 2.76)	0.026 (-0.071, 0.123)
High SDI	Switzerland	8775204 (8021728, 9564584)	27.82	90575 (78489, 104340)	15.84 (11.39, 20.77)	1032.17 (894.44, 1189.03)	-9.37 (-12.85, -5.51)	1267.11 (1078.48, 1476.94)	1.21 (-2.25, 5.07)	0.01 (-0.09, 0.11)
High SDI	Taiwan (Province of China)	23620243 (21658624, 25443226)	15.79	219034 (189728, 251903)	-0.38 (-7.72, 7.73)	927.32 (803.24, 1066.47)	-13.96 (-20.3, -6.96)	1029.54 (869.27, 1179.43)	2.45 (-3.17, 8.84)	0.195 (0.102, 0.288)
High SDI	United Arab Emirates	9241704 (7764642, 10592699)	393.66	103202 (85999, 123478)	338.51 (291.07, 393.54)	1116.7 (930.55, 1336.1)	-11.17 (-20.78, -0.02)	1148.74 (968.65, 1333.53)	0.33 (-0.23, 0.96)	-0.053 (-0.148, 0.042)
High SDI	United Kingdom	67220447 (60468668, 73925429)	16.96	773281 (683092, 862506)	10.09 (7.3, 13.25)	1150.37 (1016.2, 1283.1)	-5.87 (-8.26, -3.17)	1362.19 (1191.21, 1526.62)	0.62 (-1.77, 3.26)	0.006 (-0.091, 0.103)
High SDI	United States of America	327978730 (285959303, 369324168)	29.33	3808099 (3369434, 4231452)	15.77 (12.25, 19.27)	1161.08 (1027.33, 1290.16)	-10.48 (-13.21, -7.78)	1322.21 (1165.7, 1473.82)	-1.69 (-4.37, 1.09)	-0.022 (-0.12, 0.076)
High-middle SDI	American Samoa	41847290 (36020518, 47461912)	65.52	657 (553, 760)	13.45 (10.2, 17.03)	1183.18 (997.16, 1369.85)	-1.01 (-3.85, 2.12)	1137.74 (967.34, 1308.86)	0.47 (0.34, 0.62)	0.032 (-0.058, 0.123)
High-middle SDI	Antigua and Barbuda	88489 (77626, 98864)	45.73	933 (803, 1069)	34.75 (28.48, 41.76)	1054.12 (907.6, 1208.14)	-7.53 (-11.83, -2.72)	1089.7 (922.93, 1258.5)	-0.46 (-0.53, -0.38)	-0.026 (-0.125, 0.073)
High-middle SDI	Argentina	45115284 (39507183, 51073381)	36.21	413398 (352573, 474498)	37.42 (30.94, 45.23)	916.31 (781.49, 1051.75)	0.89 (-3.87, 6.62)	931.16 (785.46, 1072.85)	3.28 (-1.13, 8.75)	0.103 (-0.003, 0.21)
High-middle SDI	Bahamas	376940 (330381, 424689)	46.98	4149 (3562, 4756)	34.01 (28.78, 40.24)	1100.79 (944.93, 1261.78)	-8.82 (-12.38, -4.59)	1093.07 (925.82, 1262.24)	0.19 (0.15, 0.24)	-0.002 (-0.1, 0.097)
High-middle SDI	Bahrain	1442691 (1254829, 1605903)	183.97	16696 (14309, 19280)	151.57 (132.14, 176.25)	1157.27 (991.84, 1336.39)	-11.41 (-18.25, -2.72)	1189.69 (1004.88, 1375.76)	0.4 (0.05, 0.83)	-0.066 (-0.159, 0.027)
High-middle SDI	Barbados	297771 (263601, 334647)	17.28	2851 (2468, 3262)	2.07 (-1.72, 6.7)	957.4 (828.89, 1095.32)	-12.97 (-16.2, -9.03)	1088.4 (921.89, 1256.73)	-0.22 (-0.27, -0.18)	-0.016 (-0.115, 0.083)
High-middle SDI	Belarus	9500785 (8345414, 10677716)	-9.26	84195 (73159, 96262)	-17.12 (-19.85, -14.2)	886.19 (770.03, 1013.2)	-8.66 (-11.67, -5.44)	999.65 (851.95, 1153.75)	-0.45 (-0.54, -0.38)	-0.009 (-0.102, 0.085)
High-middle SDI	Bosnia and Herzegovina	3299982 (2949576, 3649228)	-27.28	31011 (26936, 35590)	-38.13 (-40.62, -35.29)	939.72 (816.24, 1078.5)	-14.92 (-18.34, -11.02)	1073.76 (920.59, 1227.78)	0.34 (0.23, 0.45)	-0.011 (-0.104, 0.081)
High-middle SDI	Bulgaria	6934625 (6359976, 7553856)	-20.12	62011 (53856, 70545)	-29.39 (-31.62, -27.02)	894.22 (776.62, 1017.29)	-11.6 (-14.4, -8.64)	1069.14 (915.74, 1222.82)	-0.56 (-0.66, -0.47)	-0.02 (-0.112, 0.072)
High-middle SDI	Chile	18198359 (16753483, 19617223)	37.03	164709 (140691, 189664)	25.55 (20.1, 31.86)	905.08 (773.1, 1042.2)	-8.38 (-12.36, -3.78)	953.13 (810.61, 1105.39)	1.35 (-2.22, 5.07)	0.016 (-0.09, 0.121)

Table 2 (Continued)

SDI		Population		Incidence		All-age incidence rate		Age-standardized incidence rate		Net drift of incidence rate from APC model, % per year
Quintil	Country	Number in 2019	Percent change 1990-2019, %	Number in 2019	Percent change 1990-2019, %	Rate in 2019	Percent change 1990-2019, %	Rate in 2019	Percent change 1990-2019, %	
High-middle SDI	Cook Islands	17987 (16599, 19575)	-5.44	194 (167, 224)	-14.33 (-17.72, -10.48)	1078.89 (927.17, 1243.06)	-9.4 (-12.99, -5.34)	1148.32 (975.77, 1321.18)	1.52 (1.28, 1.77)	0.071 (-0.02, 0.162)
High-middle SDI	Croatia	11358510 (10094746, 12738845)	4.86	105079 (90448, 119879)	-11.03 (-15.06, -6.34)	925.11 (796.3, 1055.41)	-15.16 (-19, -10.68)	1078.66 (914.51, 1246.37)	-0.37 (-0.45, -0.3)	-0.037 (-0.13, 0.055)
High-middle SDI	Dominica	68681 (60139, 77089)	-7.19	708 (608, 809)	-14.65 (-18.45, -10.61)	1030.37 (884.62, 1177.91)	-8.04 (-12.13, -3.68)	1077.75 (913.53, 1245.52)	0.05 (-0.19, 0.26)	-0.071 (-0.17, 0.028)
High-middle SDI	Greece	10337172 (9070617, 11489319)	-0.5	111725 (96564, 128286)	-17.31 (-21.57, -12.94)	1080.81 (934.14, 1241.02)	-16.89 (-21.18, -12.5)	1394.18 (1180.03, 1592.38)	-0.49 (-4.29, 3.03)	0.009 (-0.089, 0.107)
High-middle SDI	Greenland	56188 (51528, 60808)	1.1	644 (555, 736)	-10.54 (-13.4, -7.14)	1145.29 (987.56, 1309.33)	-11.52 (-14.35, -8.15)	1249.48 (1062.44, 1426.64)	1.91 (1.49, 2.37)	0.03 (-0.072, 0.133)
High-middle SDI	Hungary	9674413 (8515522, 10788963)	-6.91	88897 (77170, 101688)	-17.28 (-20.2, -14.43)	918.89 (797.67, 1051.11)	-11.14 (-14.27, -8.08)	1072.81 (919.56, 1226.61)	-0.31 (-0.37, -0.26)	-0.018 (-0.11, 0.074)
High-middle SDI	Israel	9309583 (8164697, 10550907)	87.62	119463 (102102, 137934)	71.88 (69.37, 74.56)	1283.23 (1096.74, 1481.64)	-8.39 (-9.73, -6.96)	1352.28 (1154.54, 1559.68)	-0.23 (-0.31, -0.15)	-0.018 (-0.116, 0.081)
High-middle SDI	Italy	60313170 (55356080, 64983929)	6.19	693328 (617048, 775956)	-8.98 (-12.22, -5.65)	1149.55 (1023.07, 1286.54)	-14.29 (-17.34, -11.14)	1528.35 (1345.37, 1709.32)	4.14 (1.79, 6.72)	0.149 (0.053, 0.245)
High-middle SDI	Jordan	11636717 (10588262, 12678912)	208.41	155009 (130146, 180094)	199.11 (186.58, 212.33)	1332.07 (1118.41, 1547.63)	-3.01 (-7.08, 1.27)	1133.82 (964.25, 1305.46)	-0.61 (-0.8, -0.45)	-0.029 (-0.12, 0.062)
High-middle SDI	Kazakhstan	18392068 (16794137, 19921602)	12.37	197636 (168472, 226756)	7.4 (4.61, 10.41)	1074.57 (916, 1232.9)	-4.42 (-6.91, -1.74)	1083.5 (924.64, 1249.1)	-0.02 (-0.08, 0.04)	-0.004 (-0.097, 0.088)
High-middle SDI	Lebanon	5177069 (4455291, 5928542)	58.07	63410 (53956, 72932)	51.93 (44.81, 59.65)	1224.83 (1042.2, 1408.75)	-3.88 (-8.39, 1)	1235.06 (1046.86, 1423.53)	-0.36 (-0.54, -0.2)	0.006 (-0.084, 0.097)
High-middle SDI	Libya	6735543 (5705382, 7670829)	58.97	87010 (74572, 100028)	51.26 (37.78, 65.36)	1291.8 (1107.14, 1485.08)	-4.85 (-13.33, 4.02)	1227.79 (1041.41, 1416.58)	1.1 (0.8, 1.44)	0.037 (-0.054, 0.128)
High-middle SDI	Malaysia	31301402 (27339262, 35190542)	77.29	384092 (327678, 441173)	75.73 (67.22, 84.29)	1227.08 (1046.85, 1409.44)	-0.88 (-5.68, 3.95)	1176.49 (998.37, 1345.65)	1.6 (-1.81, 4.95)	0.018 (-0.072, 0.107)
High-middle SDI	Malta	439221 (389215, 489626)	18.49	4647 (4039, 5336)	-4.54 (-7.82, -1.37)	1057.97 (919.51, 1214.99)	-19.44 (-22.2, -16.76)	1346.56 (1148.7, 1553.27)	-0.33 (-0.43, -0.21)	-0.033 (-0.132, 0.065)
High-middle SDI	Montenegro	620340 (545881, 695553)	-0.86	6071 (5281, 6930)	-10.71 (-13.54, -7.68)	978.65 (851.26, 1117.09)	-9.94 (-12.79, -6.88)	1070.6 (917.85, 1224.12)	-0.07 (-0.16, 0.02)	-0.005 (-0.098, 0.087)
High-middle SDI	Niue	1672 (1472, 1877)	-28.15	18 (16, 21)	-31.43 (-34.53, -28.48)	1085.14 (930.12, 1251.88)	-4.57 (-8.88, -0.45)	1075.02 (917.4, 1239.42)	0.01 (-0.11, 0.14)	-0.014 (-0.104, 0.076)
High-middle SDI	North Macedonia	2152731 (1785469, 2527622)	6.81	21159 (18340, 24220)	-4.37 (-8.3, -0.31)	982.88 (851.95, 1125.09)	-10.47 (-14.15, -6.67)	1066.47 (913.08, 1220)	-0.5 (-0.57, -0.44)	-0.024 (-0.117, 0.068)
High-middle SDI	Northern Mariana Islands	42494 (37226, 47877)	-6.39	457 (388, 528)	-19.63 (-25.4, -13.36)	1075.58 (913.63, 1241.84)	-14.14 (-20.31, -7.44)	1130.25 (961.65, 1301.21)	0.05 (-0.42, 0.61)	0.063 (-0.028, 0.155)
High-middle SDI	Oman	4583999 (4209600, 4952285)	135.9	55853 (47175, 65125)	119.22 (97.67, 141.3)	1218.43 (1029.12, 1420.69)	-7.07 (-16.21, 2.29)	1153.51 (973.24, 1340.26)	-1.05 (-1.42, -0.68)	-0.09 (-0.183, 0.003)
High-middle SDI	Palau	18007 (16272, 19568)	16.9	194 (167, 224)	3.15 (-2.82, 10.24)	1077.37 (927.02, 1244.19)	-11.77 (-16.87, -5.7)	1112.59 (944.28, 1282.69)	-1.73 (-2.15, -1.37)	-0.043 (-0.134, 0.048)
High-middle SDI	Poland	38434445 (35378981, 41364935)	0.72	377465 (335795, 423558)	-10.92 (-13.8, -7.98)	982.1 (873.68, 1102.03)	-11.57 (-14.42, -8.65)	1108.24 (975.98, 1237.23)	-0.24 (-0.28, -0.21)	-0.019 (-0.109, 0.071)
High-middle SDI	Portugal	10651263 (9433153, 11909037)	5.07	113298 (97999, 130999)	-14.63 (-18.05, -10.73)	1063.71 (920.07, 1229.89)	-18.75 (-22, -15.04)	1357.12 (1159.79, 1565.05)	-0.08 (-0.12, -0.04)	-0.004 (-0.102, 0.095)
High-middle SDI	Romania	19237066 (17030070, 21542480)	-17.78	177855 (154576, 202643)	-27.98 (-30.86, -24.83)	924.54 (803.53, 1053.4)	-12.41 (-15.91, -8.58)	1070.58 (917.32, 1224.24)	-0.4 (-0.46, -0.35)	-0.016 (-0.108, 0.077)
High-middle SDI	Saint Kitts and Nevis	59508 (48010, 70882)	43.9	622 (537, 715)	30.62 (22.74, 39.42)	1045.83 (901.66, 1201.18)	-9.23 (-14.71, -3.12)	1084.29 (918.82, 1252.31)	-0.37 (-0.5, -0.25)	-0.048 (-0.147, 0.051)
High-middle SDI	Serbia	8746785 (7829777, 9730592)	-6.92	82700 (72042, 94056)	-15.97 (-17.82, -13.96)	945.49 (823.64, 1075.32)	-9.72 (-11.7, -7.56)	1067.58 (915.62, 1220.59)	-0.78 (-0.98, -0.6)	-0.006 (-0.099, 0.086)
High-middle SDI	Seychelles	102145 (89532, 114508)	39.87	1241 (1066, 1423)	27.99 (21.11, 35.84)	1215.26 (1043.96, 1393.54)	-8.49 (-13.41, -2.88)	1242.89 (1056.34, 1427.45)	-1.04 (-1.26, -0.82)	-0.057 (-0.145, 0.03)
High-middle SDI	Spain	46021218 (42087988, 49981539)	18.67	500845 (434641, 581516)	-2.03 (-6.93, 3.28)	1088.29 (944.44, 1263.58)	-17.44 (-21.57, -12.97)	1361.55 (1152.57, 1569.45)	-0.11 (-0.15, -0.07)	0.008 (-0.09, 0.106)
High-middle SDI	Trinidad and Tobago	1387457 (1226317, 1549441)	15.33	14008 (12116, 16004)	-0.72 (-6.02, 5.29)	1009.6 (873.25, 1153.47)	-13.92 (-18.51, -8.7)	1080.99 (916.22, 1248.82)	-0.29 (-0.32, -0.26)	-0.011 (-0.11, 0.089)
High-middle SDI	Turkey	81359693 (71366134, 91236684)	36.12	961458 (826618, 1099316)	23.69 (15.54, 32.74)	1181.74 (1016, 1351.18)	-9.12 (-15.12, -2.48)	1200.24 (1023.9, 1384.35)	0.26 (-3.03, 4.43)	-0.061 (-0.152, 0.031)
High-middle SDI	Ukraine	44042431 (35745535, 52268016)	-16.37	408622 (364761, 457119)	-21.33 (-23.36, -19.31)	927.79 (828.2, 1037.91)	-5.93 (-8.36, -3.51)	1032.62 (909.01, 1157.48)	-0.38 (-0.42, -0.34)	-0.009 (-0.1, 0.083)
High-middle SDI	United States Virgin Islands	103985 (91185, 116694)	-1.91	968 (837, 1106)	-19.68 (-22.05, -16.81)	931.29 (804.74, 1063.45)	-18.11 (-20.53, -15.19)	1094.45 (927.39, 1262.77)	-0.2 (-0.26, -0.14)	0.005 (-0.094, 0.103)
Low SDI	Afghanistan	83064 (76169, 89694)	53.56	524948 (430066, 623815)	255.03 (247.58, 264.19)	1371.43 (1123.55, 1629.72)	5.91 (3.69, 8.64)	1228.99 (1042.76, 1417.99)	-2.22 (-2.68, -1.79)	-0.056 (-0.146, 0.035)
Low SDI	Angola	30138521 (27054840, 33116641)	192.09	329629 (270341, 388778)	203.62 (200.61, 207.06)	1093.71 (896.99, 1289.97)	3.95 (2.92, 5.13)	1018.17 (866.2, 1172.98)	1.05 (0.9, 1.23)	0.052 (-0.044, 0.148)
Low SDI	Bangladesh	159259850 (141199841, 177852818)	46.04	2032486 (1725406, 2347612)	43.91 (35.07, 52.16)	1276.21 (1083.39, 1474.08)	-1.46 (-7.51, 4.19)	1207.38 (1028.34, 1390.85)	0.99 (0.82, 1.19)	0.055 (-0.037, 0.147)
Low SDI	Benin	12665751 (11316886, 13983186)	161.01	163568 (135083, 194388)	168.57 (162.46, 174.02)	1291.42 (1066.52, 1534.75)	2.9 (0.56, 4.98)	1183.4 (1010.76, 1371.15)	-0.43 (-0.66, -0.22)	-0.013 (-0.105, 0.08)
Low SDI	Bhutan	754250 (696960, 815433)	23.21	9574 (8123, 11076)	19.93 (12.88, 26.83)	1269.37 (1076.96, 1468.47)	-2.66 (-8.38, 2.94)	1194.46 (1015.79, 1375.15)	0.44 (0.32, 0.55)	-0.012 (-0.104, 0.08)
Low SDI	Burkina Faso	22691773 (19383320, 26173262)	137.42	291571 (240510, 346170)	141.57 (136.46, 146.23)	1284.92 (1059.9, 1525.53)	1.75 (-0.41, 3.71)	1186.7 (1013.95, 1374.24)	-0.45 (-0.59, -0.32)	-0.006 (-0.099, 0.086)
Low SDI	Burundi	11934361 (10304612, 13532061)	114.23	93972 (77090, 110244)	121.16 (118.96, 123.31)	787.41 (645.95, 923.75)	3.24 (2.21, 4.24)	749.42 (634.15, 862.48)	-0.7 (-0.95, -0.51)	-0.082 (-0.188, 0.025)
Low SDI	Cambodia	16603118 (14206180, 18867660)	60.02	220050 (186345, 255147)	59.13 (50.68, 66.75)	1325.36 (1122.35, 1536.74)	-0.56 (-5.84, 4.21)	1260.16 (1071.71, 1445.88)	-1.29 (-1.53, -1.09)	-0.032 (-0.119, 0.055)
Low SDI	Central African Republic	5299863 (4459289, 6192557)	93.14	57939 (48134, 67649)	100.49 (98.13, 102.64)	1093.22 (908.21, 1276.43)	3.81 (2.58, 4.92)	1015.18 (863.7, 1168.83)	0.09 (-0.02, 0.2)	-0.005 (-0.101, 0.091)
Low SDI	Chad	16398860 (14327391, 18680096)	172.32	209915 (171330, 251673)	182.46 (179.63, 185.05)	1280.06 (1044.77, 1534.7)	3.73 (2.69, 4.68)	1183 (1009.5, 1371.02)	-0.5 (-0.6, -0.4)	-0.048 (-0.14, 0.045)
Low SDI	Comoros	714351 (593291, 837233)	53.38	5665 (4749, 6522)	56.81 (49.91, 63.42)	792.97 (664.77, 912.93)	2.24 (-2.26, 6.55)	747.38 (631.85, 858.56)	-0.61 (-0.75, -0.48)	-0.015 (-0.121, 0.091)
Low SDI	Côte d'Ivoire	4247902 (3748360, 4764237)	-13.32	38600 (33638, 44088)	-23.9 (-26.62, -20.89)	908.68 (791.88, 1037.87)	-12.21 (-15.35, -8.73)	1065.27 (908.98, 1223.15)	-0.76 (-3.84, 2.73)	-0.002 (-0.095, 0.091)
Low SDI	Democratic Republic of the Congo	87670444 (61748630, 112590350)	127.18	961772 (795822, 1127736)	138.88 (136.2, 141.19)	1097.03 (907.74, 1286.34)	5.15 (3.97, 6.17)	1008.8 (858.6, 1161.17)	-0.51 (-0.64, -0.39)	-0.019 (-0.115, 0.077)
Low SDI	Djibouti	1202797 (1050469, 1362651)	147.48	9367 (7873, 10819)	149.81 (138.65, 162.74)	778.74 (654.53, 899.5)	0.94 (-3.57, 6.16)	737.19 (623.51, 848.92)	0.51 (0.28, 0.76)	-0.005 (-0.112, 0.102)
Low SDI	Eritrea	6711213 (4780648, 8595693)	123.6	54544 (45171, 63511)	132.42 (126.2, 137.63)	812.72 (673.07, 946.34)	3.94 (1.16, 6.27)	746.88 (631.34, 857.86)	-0.43 (-0.58, -0.31)	-0.026 (-0.132, 0.079)
Low SDI	Ethiopia	107591164 (92024285, 122776186)	109.36	794458 (680384, 906219)	124.91 (117.31, 131.89)	738.4 (632.38, 842.28)	7.43 (3.8, 10.76)	692.59 (605.2, 776.68)	1.62 (-0.43, 4.02)	0.094 (-0.015, 0.203)
Low SDI	Gambia	2245866 (2028000, 2476236)	126.41	29740 (24514, 35192)	136.5 (131.09, 141.16)	1324.21 (1091.51, 1566.96)	4.46 (2.07, 6.51)	1182.72 (1009.41, 1370.73)	0.36 (0.23, 0.51)	0.046 (-0.047, 0.139)
Low SDI	Guinea	12643149 (11365936, 13951889)	104.37	164161 (135643, 194962)	119.12 (117.28, 120.93)	1298.42 (1072.86, 1542.04)	7.22 (6.32, 8.1)	1189.12 (1015.61, 1377.39)	0.2 (0.14, 0.26)	0.003 (-0.089, 0.096)
Low SDI	Guinea-Bissau	1901191 (1666339, 2146164)	88.74	25209 (20810, 29807)	95.14 (90.8, 99.27)	1325.98 (1094.55, 1567.78)	3.39 (1.09, 5.58)	1185.83 (1013.03, 1373.57)	-0.28 (-0.35, -0.22)	0.001 (-0.091, 0.093)
Low SDI	Haiti	12402099 (10373558, 14713362)	95.13	147862 (124909, 172189)	96.45 (90.38, 102.6)	1192.24 (1007.16, 1388.38)	0.67 (-2.44, 3.83)	1130.05 (960.24, 1300.2)	-0.07 (-0.23, 0.12)	0.024 (-0.075, 0.123)

Table 2 (Continued)

SDI		Population		Incidence		All-age incidence rate		Age-standardized incidence rate		Net drift of incidence rate
Quintil	Country	Number in 2019	Percent change 1990-2019, %	Number in 2019	Percent change 1990-2019, %	Rate in 2019	Percent change 1990-2019, %	Rate in 2019	Percent change 1990-2019, %	from APC model, % per year
Low SDI	Liberia	4789907 (4131045, 5420863)	143.86	63422 (52686, 74695)	153.98 (143.84, 163.7)	1324.07 (1099.93, 1559.43)	4.15 (-0.01, 8.14)	1177.18 (1004.14, 1364.23)	-0.75 (-0.96, -0.55)	-0.012 (-0.105, 0.081)
Low SDI	Madagascar	26690344 (20373798, 32844324)	123.32	214595 (177265, 250714)	132.56 (128.63, 136.31)	804.02 (664.15, 939.34)	4.14 (2.38, 5.81)	749.15 (633.47, 861.1)	-0.1 (-0.16, -0.06)	0.007 (-0.099, 0.113)
Low SDI	Malawi	18442238 (17149774, 19745167)	93	153236 (125625, 179544)	107.72 (104.86, 110.7)	830.9 (681.18, 973.55)	7.63 (6.15, 9.17)	1106.03 (937.78, 1286.69)	0.16 (0.08, 0.23)	0.021 (-0.084, 0.127)
Low SDI	Mali	21917467 (19126101, 24868428)	152.72	281007 (231165, 335036)	163.69 (160.24, 167.19)	1282.12 (1054.71, 1528.63)	4.34 (2.98, 5.73)	1181.96 (1008.65, 1369.39)	-0.27 (-0.36, -0.19)	-0.018 (-0.111, 0.074)
Low SDI	Mozambique	29528037 (27057907, 31808337)	125.9	235786 (192457, 278047)	131.49 (128.38, 134.84)	798.52 (651.78, 941.64)	2.47 (1.1, 3.96)	757.63 (641.18, 870.89)	-0.36 (-0.47, -0.24)	0 (-0.105, 0.105)
Low SDI	Nepal	30416382 (26611408, 34238807)	55.69	407499 (342010, 471369)	59.15 (50.16, 67.42)	1339.74 (1124.43, 1549.72)	2.23 (-3.55, 7.54)	1253.67 (1066.81, 1436.99)	1.2 (-2.12, 4.75)	0.057 (-0.033, 0.148)
Low SDI	Niger	23295353 (20797660, 25931631)	190.36	297024 (241865, 356953)	194.41 (190.66, 197.86)	1275.04 (1038.26, 1532.29)	1.4 (0.1, 2.58)	1181.74 (1009.63, 1369.54)	-0.06 (-0.19, 0.1)	0.027 (-0.066, 0.12)
Low SDI	Pakistan	224062847 (207077327, 241657356)	98.57	2901753 (2510393, 3283346)	106.44 (98.75, 114.98)	1295.06 (1120.4, 1465.37)	3.96 (0.09, 8.26)	1192.42 (1048.13, 1336.61)	-0.5 (-3.42, 2.48)	0.005 (-0.085, 0.096)
Low SDI	Papua New Guinea	9866614 (8688195, 10952388)	141.39	117845 (99266, 137101)	140.14 (135.7, 145)	1194.38 (1006.08, 1389.54)	-0.52 (-2.35, 1.5)	1131.86 (962.13, 1302.63)	0.16 (0.07, 0.26)	-0.006 (-0.097, 0.085)
Low SDI	Rwanda	12688117 (11344258, 14076210)	76.89	103630 (86047, 120538)	86.69 (80.72, 91.69)	816.75 (678.17, 950.01)	5.54 (2.16, 8.36)	753.53 (637.23, 865.87)	-0.11 (-0.14, -0.07)	0.008 (-0.097, 0.114)
Low SDI	Senegal	15134067 (13503992, 16852509)	98.59	196529 (162405, 231444)	102.93 (97.29, 107.88)	1298.59 (1073.11, 1529.29)	2.18 (-0.65, 4.68)	1177.82 (1006.17, 1363.88)	-0.74 (-0.9, -0.6)	-0.001 (-0.094, 0.091)
Low SDI	Sierra Leone	8284755 (7526348, 9080218)	126.85	108308 (89662, 127870)	138.2 (133.14, 142.5)	1307.31 (1082.26, 1543.44)	5 (2.77, 6.9)	1180.77 (1007.34, 1368.73)	-0.45 (-0.53, -0.37)	-0.021 (-0.113, 0.072)
Low SDI	Solomon Islands	655632 (570724, 742583)	92.58	7948 (6658, 9278)	95.63 (90.44, 101.72)	1212.29 (1015.45, 1415.17)	1.58 (-1.11, 4.74)	1136.15 (965.86, 1307.2)	0.4 (0.31, 0.51)	0.046 (-0.045, 0.137)
Low SDI	Somalia	20343112 (15201445, 25703627)	184.62	159031 (130261, 187287)	185.57 (179.08, 192.99)	781.74 (640.32, 920.64)	0.33 (-1.94, 2.94)	745.17 (629.94, 856.02)	0 (-0.21, 0.21)	0.012 (-0.093, 0.118)
Low SDI	South Sudan	9282963 (8050826, 10613472)	58.43	73685 (60432, 86571)	61.52 (57.39, 65.11)	793.77 (651, 932.58)	1.95 (-0.65, 4.22)	752.33 (636.7, 865.13)	2.09 (1.71, 2.43)	0.081 (-0.026, 0.188)
Low SDI	Togo	7921527 (6943022, 8897862)	116.26	103010 (85542, 121445)	117.19 (110.94, 123.47)	1300.38 (1079.86, 1533.1)	0.43 (-2.46, 3.34)	1182.98 (1010.58, 1369.3)	-0.35 (-0.45, -0.25)	0.008 (-0.084, 0.1)
Low SDI	Uganda	41117856 (37023696, 44955511)	137.46	329979 (269096, 389011)	150.65 (149.26, 152.15)	802.52 (654.45, 946.09)	5.55 (4.97, 6.18)	753.82 (638.02, 866.67)	0.2 (0.07, 0.35)	0.028 (-0.078, 0.133)
Low SDI	United Republic of Tanzania	56736116 (50495485, 63230443)	119.05	458427 (376954, 538404)	134 (122.03, 146.11)	808 (664.4, 948.96)	6.82 (1.36, 12.36)	768.16 (651.85, 888.12)	4.15 (-0.42, 8.44)	0.151 (0.045, 0.258)
Low SDI	Vanuatu	294550 (267953, 320614)	94.57	3583 (3007, 4176)	98.53 (95.04, 101.73)	1216.4 (1020.85, 1417.9)	2.04 (0.24, 3.68)	753.91 (637.66, 866.65)	0.38 (0.32, 0.44)	0.033 (-0.058, 0.124)
Low SDI	Yemen	31502896 (26596895, 36775107)	129.46	441953 (365086, 521903)	136.56 (126.06, 146.87)	1402.9 (1158.9, 1656.68)	3.09 (-1.48, 7.58)	1236.13 (1049.12, 1424.27)	-0.12 (-0.28, 0.04)	-0.001 (-0.091, 0.09)
Low SDI	Zimbabwe	15010852 (13317204, 16650709)	45.2	166585 (138904, 193820)	46.43 (42.28, 50.5)	1109.76 (925.36, 1291.2)	0.85 (-2.01, 3.65)	1018.65 (866.54, 1173.46)	0.25 (0.16, 0.35)	0.039 (-0.057, 0.135)
Low-middle SDI	Belize	410094 (358840, 459148)	120.62	4881 (4116, 5663)	117.09 (105.75, 128.92)	1190.32 (1003.78, 1380.86)	-1.6 (-6.74, 3.76)	1089.06 (922.4, 1257.84)	0.75 (0.6, 0.92)	0.033 (-0.067, 0.133)
Low-middle SDI	Bolivia (Plurinational State of)	12011750 (10641659, 13418191)	87.07	107389 (89923, 125836)	76.43 (70.05, 83.1)	894.03 (748.62, 1047.6)	-5.69 (-9.1, -2.12)	871.55 (731.82, 1017.05)	-0.67 (-0.76, -0.58)	-0.028 (-0.135, 0.079)
Low-middle SDI	Cabo Verde	563563 (494550, 632144)	60.28	6988 (5970, 8116)	57.18 (46.92, 67.5)	1239.95 (1059.27, 1440.19)	-1.93 (-8.33, 4.5)	1172.82 (1000.77, 1359.45)	-1.83 (-2.28, -1.41)	-0.087 (-0.179, 0.004)
Low-middle SDI	Cameroon	29101868 (24782960, 33604810)	180.07	385380 (317602, 456052)	195.22 (189.43, 200.29)	1324.25 (1091.35, 1567.09)	5.41 (3.34, 7.22)	1179.41 (1006.74, 1366.53)	-0.33 (-0.44, -0.23)	-0.01 (-0.102, 0.083)
Low-middle SDI	Congo	5265846 (4507068, 6008799)	115.39	57537 (48274, 66664)	119.04 (113.08, 125.87)	1092.65 (916.74, 1265.98)	1.7 (-1.07, 4.87)	1012.08 (861.27, 1165.36)	-0.27 (-0.42, -0.15)	0 (-0.107, 0.107)
Low-middle SDI	Democratic People's Republic of Korea	26232861 (22628875, 29910477)	24.59	249057 (213957, 286635)	20.77 (16.63, 25.07)	949.41 (815.61, 1092.65)	-3.06 (-6.39, 0.38)	968.22 (827.28, 1112.27)	-1.99 (-2.22, -1.74)	-0.087 (-0.179, 0.005)
Low-middle SDI	Dominican Republic	10881855 (9629751, 12279753)	51.07	120172 (102234, 138630)	39.94 (35.62, 45.26)	1104.34 (939.49, 1273.95)	-7.37 (-10.23, -3.85)	1082.09 (917.1, 1250.02)	-1.18 (-1.33, -1.02)	-0.045 (-0.144, 0.055)
Low-middle SDI	El Salvador	6256143 (5393104, 7102867)	18.77	71051 (60262, 82589)	10.32 (5.06, 15.7)	1135.71 (963.25, 1320.12)	-7.11 (-11.54, -2.58)	1113.04 (944.32, 1295.84)	0.67 (0.41, 0.98)	0.068 (-0.03, 0.166)
Low-middle SDI	Eswatini	1142109 (1051093, 1230315)	41.58	12714 (10662, 14795)	43.23 (36.53, 49.35)	1113.2 (933.54, 1295.4)	1.17 (-3.57, 5.49)	1013.61 (862.16, 1167.24)	-1.07 (-1.26, -0.89)	-0.006 (-0.102, 0.089)
Low-middle SDI	Ghana	31536232 (27445271, 35185758)	110.01	410296 (344415, 480953)	114.42 (106.92, 121.13)	1301.03 (1092.13, 1525.08)	2.1 (-1.47, 5.3)	1184.34 (1011.94, 1371.49)	0.28 (0.19, 0.39)	0.034 (-0.058, 0.126)
Low-middle SDI	Guatemala	17776490 (14652057, 20925960)	123.14	213621 (178445, 250476)	125.09 (113.2, 136.06)	1201.7 (1003.82, 1409.03)	0.87 (-4.45, 5.79)	1104.94 (936.75, 1285.48)	0.37 (0.1, 0.7)	0.06 (-0.039, 0.158)
Low-middle SDI	Guyana	770705 (683792, 857060)	0.08	8661 (7361, 10003)	-6.94 (-9.97, -3.5)	1123.83 (955.07, 1297.9)	-7.02 (-10.04, -3.58)	1088.12 (921.72, 1256.67)	-0.18 (-0.29, -0.07)	0.009 (-0.09, 0.108)
Low-middle SDI	Honduras	9814396 (8823027, 10833410)	108.44	118110 (98574, 138580)	105.02 (93.62, 115.35)	1203.43 (1004.39, 1412.01)	-1.64 (-7.11, 3.32)	961.69 (845.91, 1079.18)	0.61 (0.44, 0.81)	0.042 (-0.057, 0.141)
Low-middle SDI	India	1390706968 (1237773390, 1558771721)	62.54	17931771 (15751991, 20086848)	60.81 (53.99, 67.38)	1289.4 (1132.66, 1444.36)	-1.07 (-5.26, 2.97)	1216.95 (1065.76, 1361.03)	-0.18 (-2.98, 2.39)	-0.042 (-0.132, 0.048)
Low-middle SDI	Kenya	50227709 (43651058, 56751223)	116.56	425027 (365111, 484425)	128.07 (121.79, 133.84)	846.2 (726.91, 964.46)	5.32 (2.42, 7.98)	773.71 (678.11, 872.13)	-0.13 (-0.2, -0.06)	0.004 (-0.1, 0.108)
Low-middle SDI	Kiribati	118621 (107352, 128869)	60.16	1447 (1223, 1680)	63.36 (60.86, 65.98)	1220.12 (1031.3, 1415.9)	2 (0.44, 3.63)	1145.13 (973.8, 1317.43)	0.2 (0.14, 0.28)	0.014 (-0.076, 0.103)
Low-middle SDI	Kyrgyzstan	6535459 (5697805, 7315212)	46.46	73282 (62122, 84593)	46.4 (42.73, 50.19)	1121.3 (950.54, 1294.36)	-0.04 (-2.55, 2.54)	1081.5 (922.74, 1246.79)	-0.2 (-0.25, -0.15)	-0.006 (-0.098, 0.087)
Low-middle SDI	Lao People's Democratic Republic	7158250 (6469590, 7826130)	72.42	96495 (81491, 112487)	74.3 (65.64, 81.77)	1348.03 (1138.43, 1571.43)	1.09 (-3.93, 5.42)	1257.76 (1069.15, 1443.92)	-0.71 (-0.83, -0.59)	-0.022 (-0.11, 0.065)
Low-middle SDI	Lesotho	2091588 (1910348, 2273274)	15.73	23106 (19464, 26848)	17.68 (12.25, 22.49)	1104.73 (930.6, 1283.63)	1.68 (-3.01, 5.84)	1009.28 (859.06, 1161.43)	-0.49 (-0.66, -0.33)	0.006 (-0.089, 0.102)
Low-middle SDI	Maldives	498414 (448540, 546426)	124.59	6406 (5459, 7429)	119.16 (97.05, 143.61)	1285.31 (1095.23, 1490.45)	-2.42 (-12.26, 8.47)	1212.39 (1031.19, 1394.62)	-3.33 (-4.07, -2.66)	-0.067 (-0.155, 0.022)
Low-middle SDI	Marshall Islands	56842 (49772, 63374)	24.3	694 (586, 805)	23.03 (15.64, 30.11)	1221.75 (1031.02, 1415.4)	-1.03 (-6.97, 4.67)	1134.06 (963.66, 1305.32)	0.08 (-0.04, 0.21)	-0.01 (-0.101, 0.081)
Low-middle SDI	Mauritania	4014273 (3561887, 4441999)	94.26	53061 (43701, 62545)	105.77 (102.74, 108.37)	1321.8 (1088.64, 1558.07)	5.93 (4.36, 7.26)	1183.65 (1010.84, 1371.6)	0.18 (0.14, 0.22)	-0.001 (-0.093, 0.091)
Low-middle SDI	Micronesia (Federated States of)	102116 (89819, 114354)	-2.08	1245 (1049, 1444)	-1.9 (-6.88, 2.47)	1219.32 (1026.83, 1413.85)	0.18 (-4.9, 4.64)	1134.32 (964.19, 1305.37)	0.12 (0, 0.26)	0.016 (-0.075, 0.106)
Low-middle SDI	Mongolia	3387589 (2977541, 3795389)	57.29	37328 (31734, 43012)	49.95 (40.96, 60.21)	1101.89 (936.78, 1269.7)	-4.67 (-10.38, 1.86)	1081.99 (923.12, 1247.54)	0.08 (-0.01, 0.18)	0.018 (-0.075, 0.111)
Low-middle SDI	Morocco	35952186 (32338676, 39427377)	42.12	456426 (389533, 524531)	32.82 (26.85, 39.01)	1269.54 (1083.48, 1458.97)	-6.55 (-10.75, -2.19)	1236.42 (1048.92, 1424.6)	-0.35 (-0.41, -0.29)	-0.007 (-0.098, 0.083)
Low-middle SDI	Myanmar	54676901 (48907818, 60236349)	33.02	715867 (608923, 823166)	29.71 (25.04, 34.71)	1309.27 (1113.68, 1505.51)	-2.49 (-6, 1.27)	1266.5 (1077.04, 1453.01)	0.36 (0.29, 0.45)	0.028 (-0.059, 0.115)
Low-middle SDI	Namibia	2403124 (2112720, 2685060)	70.47	26193 (22111, 30455)	71.3 (66.08, 76.41)	1089.97 (920.09, 1267.29)	0.48 (-2.58, 3.48)	1015.15 (863.77, 1169.34)	0.01 (-0.05, 0.08)	0.015 (-0.081, 0.111)
Low-middle SDI	Nauru	10551 (9292, 11846)	2.89	131 (110, 153)	8.29 (5.99, 10.04)	1245.71 (1044.6, 1450.43)	5.25 (3.01, 6.95)	1137.57 (967.48, 1308.67)	0.42 (0.24, 0.65)	0.055 (-0.035, 0.146)
Low-middle SDI	Nicaragua	6510365 (5514376, 7574280)	67.49	76629 (64481, 89561)	58.77 (49.58, 67.95)	1177.04 (990.44, 1375.67)	-5.2 (-10.69, 0.28)	1095.46 (928.81, 1273.75)	-0.8 (-0.98, -0.61)	0.004 (-0.095, 0.102)
Low-middle SDI	Nigeria	214823786 (193132521, 236573636)	138.2	2919834 (2509925, 3314180)	148.57 (139.29, 157.86)	1359.18 (1168.36, 1542.74)	4.35 (0.46, 8.25)	1231.12 (1078.12, 1373.75)	0.07 (-3.06, 3.17)	0.085 (-0.005, 0.176)

Table 2 (Continued)

SDI		Population		Incidence		All-age incidence rate		Age-standardized incidence rate		Net drift of incidence rate from APC model, % per year
Quintil	Country	Number in 2019	Percent change 1990-2019, %	Number in 2019	Percent change 1990-2019, %	Rate in 2019	Percent change 1990-2019, %	Rate in 2019	Percent change 1990-2019, %	
Low-middle SDI	Palestine	4956597 (4561835, 5329689)	139.45	68357 (56868, 80265)	148.55 (140.25, 156.8)	1379.12 (1147.31, 1619.36)	3.8 (0.34, 7.25)	1232.3 (1044.95, 1420.96)	-0.31 (-0.51, -0.16)	-0.023 (-0.113, 0.068)
Low-middle SDI	Philippines	112142764 (101580880, 121868835)	77.19	1528529 (1340029, 1709330)	72.7 (69.07, 76.49)	1363.02 (1194.93, 1524.24)	-2.54 (-4.59, -0.39)	1095.16 (930.24, 1266.01)	-0.34 (-0.38, -0.29)	0.005 (-0.081, 0.09)
Low-middle SDI	Sao Tome and Principe	205385 (182424, 229091)	69	2723 (2270, 3208)	74.7 (66.96, 81.88)	1325.68 (1105.38, 1561.89)	3.37 (-1.21, 7.63)	1176.89 (1004.19, 1364.2)	-0.46 (-0.63, -0.31)	-0.027 (-0.119, 0.065)
Low-middle SDI	Sudan	40808425 (35356109, 46009715)	102.03	566587 (470251, 665008)	109.96 (105.66, 113.95)	1388.41 (1152.34, 1629.59)	3.92 (1.8, 5.9)	1239.96 (1052.06, 1428.37)	-0.25 (-0.42, -0.09)	-0.003 (-0.093, 0.088)
Low-middle SDI	Syrian Arab Republic	14491247 (12173713, 16795620)	12.38	191070 (160765, 221406)	8.03 (0.09, 15.94)	1318.52 (1109.4, 1527.86)	-3.87 (-10.94, 3.17)	1261.87 (1074.43, 1450.52)	2.38 (1.87, 2.94)	0.063 (-0.028, 0.154)
Low-middle SDI	Tajikistan	9492414 (8213913, 10674835)	76.58	108744 (91641, 125450)	80.91 (74.93, 87.26)	1145.59 (965.41, 1321.58)	2.45 (-0.93, 6.05)	1077.7 (919.36, 1242.64)	-0.4 (-0.5, -0.29)	-0.012 (-0.105, 0.081)
Low-middle SDI	Timor-Leste	1334823 (1208832, 1447383)	70.48	18008 (14981, 20976)	76.11 (71.61, 80.99)	1349.07 (1122.31, 1571.47)	3.3 (0.66, 6.16)	1256.81 (1068.77, 1443.48)	0.62 (0.53, 0.72)	0.016 (-0.072, 0.104)
Low-middle SDI	Tuvalu	11798 (10462, 13230)	26.24	139 (117, 161)	29.03 (26.65, 31.06)	1175.14 (992.9, 1360.57)	2.21 (0.33, 3.82)	1125.95 (956.44, 1296.62)	-2.21 (-2.67, -1.8)	-0.099 (-0.189, -0.009)
Low-middle SDI	Venezuela (Bolivarian Republic of)	28068986 (24769383, 31423834)	49.07	305459 (261152, 353162)	37.36 (29.55, 45.93)	1088.24 (930.39, 1258.19)	-7.86 (-13.09, -2.11)	1097.44 (929.72, 1266.76)	2.18 (-2.45, 6.88)	0.042 (-0.057, 0.141)
Low-middle SDI	Viet Nam	96372928 (83066414, 109007012)	41.85	1225994 (1055033, 1411665)	32.56 (24.36, 41.51)	1272.14 (1094.74, 1464.79)	-6.54 (-12.33, -0.23)	1255.85 (1067.63, 1441.07)	-0.95 (-1.08, -0.82)	-0.034 (-0.121, 0.053)
Low-middle SDI	Zambia	18237683 (15885855, 20473278)	129.61	155988 (128721, 183552)	132.77 (122.71, 142.01)	855.3 (705.8, 1006.44)	1.38 (-3, 5.4)	786.87 (665.93, 909.31)	-2.1 (-5.06, 1.02)	-0.046 (-0.15, 0.058)
Middle SDI	Albania	38277536 (26161826, 50468583)	235.21	26462 (23058, 30168)	-29.77 (-33.55, -25.79)	972.74 (847.62, 1108.97)	-14.55 (-19.15, -9.7)	1069.3 (916.23, 1222.5)	0.16 (-0.16, 0.52)	0.034 (-0.059, 0.127)
Middle SDI	Algeria	2720353 (2418308, 3021834)	-17.81	526992 (447986, 606761)	50.67 (41.41, 60.68)	1259.32 (1070.53, 1449.94)	-8.97 (-14.56, -2.92)	1234.59 (1047.1, 1422.73)	0.06 (-0.01, 0.14)	-0.002 (-0.092, 0.089)
Middle SDI	Armenia	3019674 (2651852, 3385871)	-11.57	30633 (26330, 35169)	-19.44 (-22.47, -15.86)	1014.45 (871.95, 1164.66)	-8.9 (-12.33, -4.86)	1078.69 (921.19, 1243.76)	-0.49 (-0.62, -0.36)	-0.004 (-0.096, 0.089)
Middle SDI	Azerbaijan	10278674 (8953532, 11640072)	40.21	112437 (96076, 129358)	35 (29.17, 41.72)	1093.88 (934.71, 1258.51)	-3.71 (-7.87, 1.08)	1074.24 (916.94, 1238.73)	-1.08 (-1.19, -0.97)	-0.034 (-0.127, 0.058)
Middle SDI	Botswana	2338721 (2084722, 2607764)	79.67	25705 (21837, 29750)	75.95 (65.67, 86.18)	1099.12 (933.73, 1272.08)	-2.07 (-7.79, 3.62)	1011.48 (860.71, 1164.28)	-0.98 (-1.13, -0.84)	-0.014 (-0.11, 0.082)
Middle SDI	Brazil	216664814 (189879232, 242502434)	45.57	3023237 (2663324, 3393364)	27.36 (22.23, 33.36)	1395.35 (1229.24, 1566.18)	-12.51 (-16.03, -8.39)	1489.15 (1290.61, 1698.33)	5.68 (2.25, 9.06)	-0.092 (-0.257, 0.074)
Middle SDI	China	1422350422 (1239302360, 1597063538)	20.16	12939765 (11463449, 14485073)	14.4 (7.28, 21.55)	909.75 (805.95, 1018.39)	-4.79 (-10.72, 1.15)	1213.34 (1027.56, 1402.06)	7.05 (4.35, 10.24)	0.259 (0.165, 0.353)
Middle SDI	Colombia	47776679 (44174337, 51539501)	46.79	513211 (436586, 590651)	30.67 (22.03, 37.97)	1074.19 (913.81, 1236.28)	-10.98 (-16.87, -6.01)	1104.33 (931.72, 1284)	0.02 (-4.08, 4.25)	0.035 (-0.064, 0.133)
Middle SDI	Costa Rica	4716744 (4164759, 5271508)	55.16	51062 (43688, 59209)	41.04 (33.6, 48.6)	1082.57 (926.23, 1255.28)	-9.1 (-13.9, -4.23)	1101.2 (933.7, 1280.75)	0.67 (0.56, 0.79)	0.043 (-0.056, 0.142)
Middle SDI	Cuba	1313477 (1162096, 1476217)	68.83	15701 (13516, 18040)	49.92 (43.29, 57.5)	1195.4 (1029.05, 1373.49)	-11.2 (-15.13, -6.71)	1352.33 (1155.54, 1559.01)	0.39 (0.27, 0.55)	-0.002 (-0.102, 0.097)
Middle SDI	Ecuador	17588392 (15403894, 19749863)	75.42	171899 (144779, 200217)	64.39 (53.34, 73.97)	977.34 (823.15, 1138.34)	-6.29 (-12.59, -0.83)	949.72 (797.99, 1107.58)	0.57 (-4.41, 5.95)	0.018 (-0.087, 0.123)
Middle SDI	Egypt	99069551 (90571861, 107515771)	77.87	1383829 (1182631, 1579436)	85.21 (73.58, 98.14)	1396.83 (1193.74, 1594.27)	4.13 (-2.41, 11.4)	1285.08 (1107.4, 1462.88)	3.3 (-2.11, 9.29)	-0.083 (-0.174, 0.008)
Middle SDI	Equatorial Guinea	1419839 (1290598, 1552892)	229.93	16060 (13294, 18752)	265.69 (252.72, 277.18)	1131.1 (936.33, 1320.71)	10.84 (6.91, 14.32)	993.26 (845.09, 1144.71)	-3 (-3.5, -2.57)	-0.063 (-0.158, 0.033)
Middle SDI	Fiji	911248 (838786, 984189)	20.01	10666 (9045, 12323)	12.91 (9.71, 16.58)	1170.52 (992.64, 1352.28)	-5.92 (-8.58, -2.86)	1134.97 (964.59, 1306.21)	-0.13 (-0.18, -0.08)	0.001 (-0.089, 0.091)
Middle SDI	Gabon	1750038 (1566262, 1939240)	76.5	19155 (16202, 22244)	85.41 (79.08, 91.06)	1094.54 (925.81, 1271.07)	5.05 (1.46, 8.25)	1017.96 (866.13, 1172.09)	0.89 (0.68, 1.13)	0.014 (-0.082, 0.11)
Middle SDI	Georgia	3664752 (3306198, 4043348)	-33.47	35136 (30259, 40352)	-40.33 (-41.95, -38.42)	958.75 (825.68, 1101.08)	-10.31 (-12.74, -7.44)	1086.99 (920.71, 1255.63)	-1.07 (-1.17, -0.96)	-0.032 (-0.124, 0.061)
Middle SDI	Grenada	103215 (90673, 115504)	20.48	1085 (925, 1246)	9.2 (2.39, 16.61)	1050.93 (895.84, 1207.01)	-9.36 (-15.01, -3.21)	1074.56 (910.93, 1242.07)	-1.05 (-1.19, -0.92)	-0.062 (-0.161, 0.037)
Middle SDI	Indonesia	259465835 (226843397, 291997879)	39.97	3546988 (3120944, 3962866)	34.31 (29.9, 38.72)	1367.03 (1202.83, 1527.32)	-4.04 (-7.19, -0.89)	1300.63 (1142.06, 1450.74)	-0.39 (-0.47, -0.31)	-0.009 (-0.095, 0.076)
Middle SDI	Iran (Islamic Republic of)	84297882 (77330567, 91935910)	44	1093330 (967519, 1223899)	31.41 (21.87, 41.67)	1296.98 (1147.74, 1451.87)	-8.74 (-15.37, -1.62)	1274.66 (1119.7, 1426.98)	-0.71 (-3.35, 2.34)	0.055 (-0.033, 0.143)
Middle SDI	Iraq	42119490 (31429294, 52981880)	139.36	569281 (479195, 662177)	140 (127.91, 151.86)	1351.58 (1137.7, 1572.14)	0.27 (-4.79, 5.22)	1229.44 (1042.54, 1418.39)	-0.12 (-0.17, -0.08)	0.007 (-0.084, 0.098)
Middle SDI	Jamaica	2810754 (2482603, 3132031)	18.91	30689 (26269, 35314)	10.08 (4.7, 15.68)	1091.83 (934.61, 1256.38)	-7.43 (-11.95, -2.72)	1298.91 (1140.91, 1448.85)	-0.39 (-0.52, -0.26)	-0.015 (-0.114, 0.084)
Middle SDI	Mauritius	1276663 (1113655, 1442942)	16.05	15182 (13113, 17458)	0.58 (-4.41, 5.99)	1189.2 (1027.11, 1367.5)	-13.34 (-17.63, -8.67)	1258.33 (1069.62, 1444.5)	0.01 (-0.02, 0.05)	-0.006 (-0.093, 0.082)
Middle SDI	Mexico	124940175 (108607132, 140630348)	46.15	1418315 (1246727, 1594501)	36.33 (29.95, 43.49)	1135.2 (997.86, 1276.21)	-6.72 (-11.09, -1.82)	1111.52 (975.04, 1254.11)	1.33 (-1.51, 4.66)	0.078 (-0.019, 0.174)
Middle SDI	Panama	4160457 (3659255, 4679229)	74.19	45455 (38523, 52831)	60.27 (56.1, 65.11)	1092.55 (925.94, 1269.84)	-7.99 (-10.38, -5.21)	1088.73 (922.29, 1265.57)	-0.06 (-0.13, 0.01)	0.015 (-0.084, 0.115)
Middle SDI	Paraguay	6930455 (5699198, 8110997)	71.31	99499 (83936, 117442)	55.33 (47.06, 63.75)	1435.68 (1211.11, 1694.58)	-9.33 (-14.16, -4.41)	1370.79 (1149.48, 1616.67)	-0.29 (-0.34, -0.23)	-0.007 (-0.104, 0.091)
Middle SDI	Peru	33995397 (31120105, 36626226)	56.44	273699 (232092, 318951)	56.27 (45.6, 67.04)	805.11 (682.72, 938.22)	-0.11 (-6.93, 6.77)	794.83 (674.56, 926.59)	7.28 (1.11, 13.95)	0.329 (0.217, 0.442)
Middle SDI	Republic of Moldova	3688191 (3095661, 4327374)	-17.05	33987 (29386, 38902)	-24.31 (-27.67, -20.92)	921.5 (796.77, 1054.78)	-8.75 (-12.8, -4.66)	998.62 (850.63, 1152.64)	-0.95 (-1.08, -0.83)	-0.032 (-0.126, 0.061)
Middle SDI	Saint Lucia	174626 (153612, 195041)	27.09	1812 (1559, 2076)	10.88 (2.7, 19.99)	1037.38 (892.86, 1188.8)	-12.76 (-19.19, -5.59)	1083.95 (918.47, 1252.2)	-0.86 (-0.96, -0.78)	-0.043 (-0.142, 0.056)
Middle SDI	Saint Vincent and the Grenadines	113144 (101218, 125637)	2.8	1180 (1011, 1352)	-11.17 (-16.46, -5.06)	1042.74 (893.77, 1194.62)	-13.59 (-18.74, -7.65)	1080.25 (915.47, 1248.41)	-0.33 (-0.44, -0.22)	-0.047 (-0.146, 0.053)
Middle SDI	Samoa	211354 (193000, 229266)	29.16	2598 (2174, 3030)	27.84 (25.48, 30.63)	1229.35 (1028.68, 1433.49)	-1.02 (-2.85, 1.13)	1132.18 (961.94, 1302.71)	0.41 (0.26, 0.56)	-0.002 (-0.092, 0.089)
Middle SDI	South Africa	55588425 (49169682, 62724694)	50.93	606270 (529331, 680997)	44.62 (40.1, 49.45)	1090.64 (952.23, 1225.07)	-4.19 (-7.18, -0.99)	1042.74 (911.08, 1167.69)	-0.59 (-0.68, -0.51)	-0.011 (-0.105, 0.082)
Middle SDI	Sri Lanka	21854452 (19445064, 24139979)	26.9	271668 (232572, 312007)	15.33 (11.59, 19.51)	1243.08 (1064.19, 1427.66)	-9.12 (-12.06, -5.83)	1264.15 (1074.99, 1450.73)	0.45 (0.36, 0.54)	0.041 (-0.046, 0.129)
Middle SDI	Suriname	575888 (512164, 648630)	48.96	6228 (5290, 7168)	38.17 (33.61, 43.74)	1081.42 (918.53, 1244.67)	-7.24 (-10.3, -3.51)	1085.03 (919.04, 1252.28)	0.17 (-0.02, 0.41)	0.016 (-0.083, 0.116)
Middle SDI	Thailand	70111586 (61328965, 78909536)	23.27	857152 (748179, 985196)	-1.89 (-8.29, 4.94)	1222.55 (1067.13, 1405.18)	-20.41 (-25.6, -14.87)	1336.04 (1138.33, 1541.84)	-4.13 (-7.79, -0.21)	-0.006 (-0.093, 0.081)
Middle SDI	Tokelau	1411 (1283, 1529)	-16.38	16 (13, 18)	-15.71 (-18, -13.27)	1105.96 (937.72, 1281.06)	0.81 (-1.93, 3.72)	1130.94 (961.96, 1303.34)	-0.98 (-1.32, -0.69)	-0.074 (-0.164, 0.016)
Middle SDI	Tonga	102350 (93644, 111195)	5.75	1212 (1016, 1409)	2.69 (0.29, 5.49)	1184.43 (992.72, 1376.71)	-2.88 (-5.16, -0.24)	1141.97 (970.86, 1313.47)	0.04 (-0.08, 0.15)	0.004 (-0.087, 0.094)
Middle SDI	Tunisia	11571604 (10423733, 12757923)	37.12	139776 (120065, 160031)	23.2 (15.97, 31.05)	1207.92 (1037.59, 1382.96)	-10.15 (-15.42, -4.43)	1239.07 (1051.9, 1427.29)	0.22 (0.12, 0.33)	0.017 (-0.074, 0.107)
Middle SDI	Turkmenistan	5083080 (4614049, 5544853)	37.19	55628 (47483, 64141)	31.52 (26.63, 37.35)	1094.37 (934.14, 1261.86)	-4.13 (-7.7, 0.12)	1068.31 (911.23, 1231.92)	-1.45 (-1.67, -1.23)	-0.045 (-0.138, 0.048)
Middle SDI	Uruguay	3436137 (3031175, 3876964)	9.45	30012 (25585, 34490)	4.52 (2.27, 6.77)	873.44 (744.58, 1003.74)	-4.5 (-6.56, -2.45)	944.17 (796.29, 1087.54)	0 (-0.04, 0.03)	0.006 (-0.1, 0.112)
Middle SDI	Uzbekistan	33677096 (25411001, 42319371)	60.77	384695 (327573, 444019)	62.42 (56.14, 69.14)	1142.3 (972.69, 1318.46)	1.03 (-2.88, 5.21)	1080.06 (921.56, 1245.25)	-0.25 (-0.33, -0.17)	-0.001 (-0.093, 0.092)

Notes:

All-age incidence rate: crude incidence rate.

Age-standardized incidence rate is computed by direct standardization with global standard population in GBD 2019.

Net drifts are estimates derived from the age-period-cohort model and denotes overall annual percentage change in incidence rate, which captures the contribution of the effects from calendar time and successive birth cohorts.

Parentheses for all GBD health estimate indicate 95% uncertainty intervals; parentheses for net drift indicate 95% confidence intervals.

SDI: Socio-demographic Index; APC: age-period-cohort.
